# Utilization of mental health services during the first year of the COVID-19 pandemic – a systematic review and meta-analysis

**DOI:** 10.1192/j.eurpsy.2025.10119

**Published:** 2026-01-13

**Authors:** Miriam Glock, Antranik Erdekian, Mike Rueb, Francesca Uhl, Ronja Husemann, Jutta Stoffers-Winterling, Saskia Lindner, Oliver Tüscher, Lars Peer Hölzel, Klaus Lieb, Kristina Adorjan, Hauke Felix Wiegand

**Affiliations:** 1 Department of Psychiatry, Psychotherapy and Psychosomatics, University Medicine Halle, Halle (Saale), Germany; 2 Department of Psychiatry and Psychotherapy, Charité University Medicine Berlin, Berlin, Germany; 3 Department of Psychiatry and Psychotherapy, University Medicine Mainz, Mainz, Germany; 4 Leibniz Institute for Resilience Research (LIR), Mainz, Germany; 5 Department of Psychiatry and Psychotherapy, University of Bern, Switzerland

**Keywords:** COVID-19, mental health services, meta-analysis, service utilization, systematic review

## Abstract

**Background:**

The COVID-19 pandemic presented significant challenges to infectious disease management and mental health services (MHS). Service demand and delivery changed due to fear of infection, economic hardships, and the psychological effects of protective measures. This systematic review with meta-analysis aims to quantify these impacts on different mental health service settings.

**Methods:**

Comprehensive searches were conducted in PubMed, Embase, and PsycINFO, focusing on studies published from the initial outbreak of COVID-19, starting in November 2019. Studies were included comparing the utilization of mental health inpatient, emergency department (ED), and outpatient services (including telemedicine and medication prescriptions) before and during the COVID-19 pandemic. A random-effects model was employed to estimate pooled effects, with study quality assessed using a modified Newcastle-Ottawa Scale.

**Results:**

Among 128 studies, significant decreases in utilization were observed during the initial phase of the pandemic for inpatient services (RR: 0.75, 95% CI: 0.67 to 0.85) and ED visits (RR: 0.87, 95% CI: 0.69 to 1.10). Outpatient services showed a similar decline (RR: 0.78, 95% CI: 0.66 to 0.92), while no significant change was found in psychotropic medication prescriptions (RR: 0.90, CI: 0.77 to 1.05). In contrast, telemedicine utilization increased significantly (RR: 7.57, 95% CI: 3.63 to 15.77).

**Conclusions:**

The findings reveal substantial shifts in mental health service utilization during the pandemic, with the largest reductions in inpatient services and significant increases in telemedicine use. These results emphasize the need for flexible healthcare models. Further research is essential to evaluate the consequences of reduced MHS utilization.

## Introduction

The COVID-19 pandemic posed a challenge not only for infectiology and intensive care but also for other essential healthcare services [1], such as mental health services (MHS). Fears of infection, post-COVID syndromes, the psychological effects of infection control measures, and economic hardships may have led to changes in the demand for MHS [2–4]. Similarly, infection control measures, resource reallocations, a focus on somatic medicine, and changes in incentive structures may have—among other factors—altered the provision of mental healthcare services [5–7].

Several studies have reviewed the effects of the COVID-19 pandemic on MHS utilization. In a systematic review, Duden et al. [8] narratively synthesized the evidence regarding challenges and changes in global MHS during the pandemic. They reported reductions in demand, access, referrals, admissions, and caseloads during the initial phases of the pandemic, followed by normalizations or even increases later on. They interpreted their results as evidence that community MHS were quite adaptable and resilient to the challenges posed by the pandemic. Another global trend was the introduction of telemedicine services [8]. Steeg et al. [9] examined presentations to MHS following self-harm. They reported a reduction in the first month of the pandemic in 2020 but a trend toward normalization in 2021 and even an increase in service use among adolescent girls. However, these latter results were based on a limited number of studies [9]. Wan Mohd Yunus et al. systematically reviewed studies on service use in children, adolescents, and young adults aged 0 to 24 years. They found decreases in service use during the early phases of the pandemic. They interpreted these findings as potentially indicative of delayed treatment and unmet needs [10]. In their systematic review of MHS use, Ahmed et al. reported decreases in inpatient admissions by 11–43%, presentations to emergency departments and walk-in services by 14–58%, and community mental health and outpatient services by 24–75%, alongside a shift from in-person to telemedicine contacts [11]. Inpatient services remained below pre-pandemic levels in late 2020 and 2021, whereas community mental health and outpatient services reported higher-than-pre-pandemic utilization [11].

These existing reviews have some limitations in estimating the effect of the pandemic on MHS utilization. They were either restricted to defined syndromes [9] or services [10] or provided only narrative syntheses [8, 11]. Furthermore, it is important to account for the considerable heterogeneity in the level of observation of the studies on MHS utilization, which ranged from single emergency departments to entire countries. Therefore, we conducted a systematic review and meta-analysis of the global literature on MHS utilization. Such a synthesis of study results is of high relevance for discussions on the evaluation of pandemic response measures in the context of their impacts on other areas of care, and for learning from a comprehensive picture of changes in order to be better prepared for future crisis situations.

## Methods

### Inclusion and exclusion criteria

We included studies that examined the utilization of the mental healthcare system and compared the period before the COVID-19 pandemic with the period during the COVID-19 pandemic, to quantify changes to pre-pandemic utilization levels. We excluded studies that only compared periods during the pandemic with intervals after the pandemic, because of assumed changes in offerings and utilization patterns in some areas of MHS after the pandemic. We only focused on studies employing quantitative research methodologies, including longitudinal studies (prospective and retrospective), cohort studies, and analyses of routine data, while excluding qualitative research. Results were categorized into three service types: inpatient, emergency department (ED), and outpatient services. Additionally, outpatient telemedicine services and outpatient medication prescriptions were examined separately. Inpatient services included planned inpatient hospitalizations, emergency inpatient admissions, or admissions resulting from visits to emergency departments. Outpatient services encompassed general practitioner visits, mental health specialist visits, outpatient telemedicine services (e.g., video calls, phone calls), individual or group psychotherapy, and outpatient prescriptions for psychotropic medications. A detailed overview of the settings for each study is provided in the supplementary material (Supplementary Material Figure S6). Only studies involving populations with a known or newly diagnosed mental disorder according to ICD-10 or DSM-5 were included. Participants had to be 18 years of age or older. In cases where the population age was not clearly stated or where there were mixed adult and adolescent populations, the authors were contacted to confirm that the study either did not include or only minimally (cutoff <15% of patients) included individuals under 18 years of age. Studies that focused on suicide or suicide attempts but not diagnosed mental disorders or utilization, as well as case reports, qualitative surveys, intervention studies, commentaries, and discussion papers, were excluded.

### Search strategy and screening process

The methods of this systematic review were predefined and registered in the International Prospective Register of Systematic Reviews (PROSPERO, registration number CRD42022334792) [12] and were conducted according to the PRISMA guidelines [13]. We searched Embase for English-, French-, and German-language sources published from November 2019—the time when the COVID-19 disease first emerged and later developed into a pandemic—up to July 2022, and PubMed and PsycINFO from November 2019 until 30.03.2025.

The search strategy combined terms related to COVID-19 (e.g., “COVID-19,” “SARS-CoV-2,” “2019-nCoV”) and mental health (e.g., “mental health,” “mental disorders,” “psychiatric disorders”) using both free-text terms and controlled vocabulary (MeSH terms). The Supplementary Material (Supplementary Material Figure S9) presents the complete search strategy for the three databases. Using Endnote [14], the studies identified were imported into Covidence [15] for title/abstract and full-text screening. To identify additional references, we manually searched the reference lists of the identified reviews. After duplicates were removed, the reviewers performed the title, abstract, and full-text screenings in tandems of two.

### Data extraction

We extracted the following data from each study: authors, study characteristics (aim of study, study design, country, setting, time periods considered), population details (age, gender), the number of visits or consultations in the respective setting, and psychiatric diagnoses according to ICD-10 or DSM-5 (for details, see Table 1). Two independent reviewers conducted the data extraction process, resolving discrepancies through discussion or consensus within the review group.

**Table 1.** Characteristics of the included studies

	Study	Country	Setting	Selected data collection periods		Study aim	Number of study participants (*n*)		Diagnostic groups according to ICD-10, when subdivided into F-diagnoses
				Before COVID-19	During COVID-19		BeforeCOVID-19	During COVID-19	
1	Abe et al. 2025	Japan	Inpatient, outpatient	April to December 2019	April to December 2020	Examine whether access to outpatient and inpatient care for psychiatric conditions was maintained in Japan during the pandemic	Total participants: 82467 RR values were taken directly from the original study		F0-F4
2	Adorjan et al. 2021	Germany	Inpatient	April to May 2019	April to May 2020	Average number of inpatient and day-care psychiatric treatments in 38 clinics	6840	4218	total
3	Ahmedani et al. 2024	USA	Outpatient, telemedicine	June 2019 to March 2020	March to December 2020	Examine population-level disruption in psychotherapy before and after the rapid shift to virtual mental health care in the United States	28,4826	270,794	total
4	Akkaoui et al. 2025	France	ED	January to December 2019	January to December 2020	description of the trends in the number of visits to the largest psychiatric emergency department in France	Total participants: 69,764. percentage reduction reported. 23.9% decrease in 2020		total
5	Alves et al. 2021	Portugal	ED, inpatient	March to May 2019	March to May 2020	visits to the psychiatric ED and hospitalizations of a hospital center	349	158	F1-F7
6	Ambrosetti et al. 2021	Switzerland	ED, inpatient	April to May 2016	April to May 2020	presentations to the adult psychiatric ED of the University Hospital of Geneva, admissions in consequence of visits	702	579	F0, F1, F3, F4, F5
7	Anderson et al. 2022	USA	ED	February to March 2019	February to March 2021	Mental health related ED visits from more than 3600 facilities from 49 states and District of Columbia	265,682	242,258	F2-F6
8	Andersson et al. 2022	Sweden	Outpatient	January to December 2019 March to May 2019	January to December 2020 March to May 2020	Number of alcohol-attributable admissions at an addiction-specialized treatment facility	1803	1675	total
9	Bakolis et al. 2021	United Kingdom	Inpatient, outpatient	January to March 2020	March to June 2020	Changes in inpatient care and community services from 10 UK providers	65,807	61,301	total
10	Balestrieri et al. 2021	Italy	ED, inpatient	March to May 2019	March to May 2020	Psychiatric consultations in 9 Italian hospital EDs, consultations that led to admissions	1649	1075	F0-F4
11	Baum et al. 2024	Germany	Inpatient, outpatient	March 2019 to February 2020	March to May 2020	Changes in utilization in the German mental health care inpatient and outpatient mental health care system	Total participants: 2,294,091 RR values were taken directly from the original study		F1-F4
12	Beghi et al. 2022	Italy	ED	May to August 2019	May to August 2020	Emergency room psychiatric consultations at all 7 public hospitals of AUSL Romagna	1220	1358	F0-F6
13	Berardelli et al. 2021	Italy	Inpatient	May 2019 to March 2020	March to December 2020	Adult psychiatric inpatients consecutively admitted to the psychiatric unit of Sant ‘Andrea Hospital	315	317	F2-F6
14	Bhagavathula et al. 2024	USA	Telemedicine	January to December 2019	January to December 2020	Impact of the COVID–19 pandemic on utilization of telehealth services for SUDs and MHC in ND and MN	25,237	1,181,248	F1
15	Boldrini et al. 2021	Italy	Inpatient	March to June 2018/2019	March to June 2020	Psychiatric admissions in 12 general psychiatric wards	3270	1280	F2, F3, F6
16	Bonello et al. 2021	Malta	Inpatient	March to June 2019	March to June 2020	Admissions to the only national mental health facility in Malta	262	172	F1-F4
17	Bruckner et al. 2023	USA	ED	January 2018 to March 2020	March to May 2020	COVID–19-related perturbations in psychiatric care in the largest safety-net hospital in Los Angeles	74,743	4414	total
18	Cafaro et al. 2022	Italy	ED, inpatient	March to May 2019	March to May 2020	Impact of COVID–19 on the EDs access of psychiatric patients in two of the main hospitals of Milan	1023	539	total
19	Capuzzi et al. 2020	Italy	ED	February to May 2019	February to May 2020	Patients receiving psychiatric consultations in two psychiatric emergency services	360	203	F1, F2, F3, F4, F6, F7
20	Caselli et al. 2023	Italy	Outpatient	January 2019 to February 2020	March 2020 to May 2021	Psychiatric consultation before and after the COVID–19 pandemic at the psychiatric outpatient services of Varese	395	346	F0-F4
21	Carr et al. 2021	United Kingdom	Outpatient, medication prescriptions	January 2010 to February 2020	March to September 2020, March to April 2020	Patients registered from 1697 UK general practices, referrals to mental health services, medication prescriptions (expected versus observed)	128,871	99,888	F3-F4
22	Chen et al. 2020a	United Kingdom	Outpatient	January to May 2019	January to May 2020	Referrals to secondary mental health care services from Cambridgeshire and Peterborough NHS Foundation Trust (CPFT)	386	328	F0, F1, F5, F6, F8
23	Chow et al. 2021	Netherlands	Outpatient, telemedicine	April to June 2019	April to June 2020	Number of care contacts of patients with mental health care professionals from a large Dutch mental healthcare institute	66,387	69,430	F1, F2, F3, F4, F5, F6, F8
24	Chu et al. 2024	Canada	Inpatient, outpatient, ED, medication	June 2018 to June 2019	June 2021 to June 2022	Describe and compare the characteristics of people with SUD and their use of healthcare services	259,497	276,459	total
25	Clerici et al. 2020	Italy	Inpatient	January to March 2019	January to March 2020	Hospital admissions in seven General Hospital Psychiatric Wards	695	648	F1, F2, F3, F6
26	Correia et al. 2024	Canada	Outpatient, inpatient	January to March 2019	January to March 2021	Incidence of mental health (MH) among perinatal people in three different COVID–19 phases	Total population: 72,242. antenatal MH diagnoses, 2019 versus 2021: aRR = 1.32 (CI = 1.20–1.46). postpartum MH diagnoses: 2019 versus 2021: aRR = 1.16 (CI = 1.08–1.25). study not included in meta-analysis due to highly specific population.		
27	Davies et al. 2021	United Kingdom	Inpatient	March to August 2019	March to August 2020	In-patient psychiatric admissions to Kent and Medway NHS and Social Care Partnership Trust	1537	1457	F0-F8
28	deDiegoRuiz et al. 2023	Spain	Inpatient	March to May 2019	March to May 2020	Sociodemographic and clinical profile of psychiatric patients admitted to Gregorio Marañón Hospital during lockdown	194	204	F2-F6
29	Der et al. 2023	USA	Mixed (ED, inpatient)	January 2016 to March 2020	April 2020 to March 2021	Proportion of hospital and emergency department encounters for MH/SUD diagnoses in the United States	45,141	10,433	Total
30	Di Lorenzo et al. 2021a	Italy	Outpatient, telemedicine	March to August 2019	March to August 2020	Urgent psychiatric consultations performed at the outpatient Mental health Center of Modena	425	488	F0-F7
31	Di Lorenzo et al. 2021b	Italy	ED, inpatient	March to August 2019	March to August 2020	Urgent psychiatric consultations in ED of two Modena General Hospitals, admissions from the ED	602	476	F2-F4
32	Di Valerio et al. 2024	Italy	Medication dispensing	January to December 2019	January to December 2020	Changes in antidepressant drug use post-COVID–19 to understand the pandemic’s effect on mental health.	341,891	336,596	Antidepressants
33	Dindar et al. 2024	Turkey	Inpatient	March to September 2019 March 2019 to September 2020	March to September 2021 March 2020 to September 2021	Effect on the clinical conditions of the patients with bipolar disorder and schizophrenia spectrum disorders in a community mental health center (CMHC)	68	60	F2, F3
34	Engels et al. 2022	Germany	Inpatient, medication prescription	January to December 2019, March to May 2019	January to December 2020, March to May 2020	A secondary data analysis based on AOK insurance data	427,811	368,543	F0-F8
35	Ettman et al. 2024	USA	Outpatient, telemedicine	November 2017 to March 2020	March 2020 to October 2022	Differences in appointment completion rates between telepsychiatry and in-person outpatient psychiatric care	294,103	292,163	Total
36	Fasshauer et al. 2021a	Germany	Inpatient	March to May 2019	March to May 2020	Number of admissions for psychiatric disorders and length of stay from 67 hospitals	4775	3327	F0-F6
37	Fasshauer et al. 2021b	Germany	Inpatient	2019	2020	Inpatient admissions for psychiatric diagnoses from 13 hospitals	22,251	19,691	F0-F9
38	Fasshauer et al. 2022	Germany	Inpatient	January to December 2019, April to June 2018/2019	January to December 2020, April to June 2020	Number of emergency admissions and length of stay from several psychiatric hospitals	25,415	22,469	F0-F6
39	Fellinger et al. 2023	Austria	Inpatient	January 2018 to December 2019	January to December 2020	Examine the association between COVID–19 lockdowns and involuntary psychiatric admissions in Austria	44,630	21,486	Total
40	Flament et al. 2021	Belgium	ED	May 2019	May 2020	Number of psychiatric visits to the ED of a university hospital	55	50	Total
41	Flodin et al. 2023	Norway Latvia Sweden Netherlands	outpatient	January 2015 to December 2021	March to December 2020	Characterize the impact of the pandemic on common mental disorders recorded prevalence in primary care	Total population: 7905 person-months. RR values were taken directly from the original study		Total
42	Fuster-Casanovas et al. 2024	Spain	Outpatient, telemedicine	January 2017 to December 2022	January to December 2020	Analyze the approach to depressive episodes and the role of eHealth in the Catalan health care system from 2017 to 2022	24,004	23,778	F3
43	Fstkchian et al. 2023	USA	Medication prescription	November to December 2019	March to April 2020	Number of patients with OUD who received a prescription	7	8	F1
44	Fu et al. 2024	United Kingdom	Medication prescription	March 2018 to March 2020	March 2020 to July 2020	Impact of pandemics on primary care before, during, and after lockdowns in England, UK	Total population: 19,356. data reported as average monthly number of prescriptions		Total
45	Gajdics et al. 2023	Hungary	Inpatient	March 2017 to March 2020	March 2020 to May 2022	Impact of the COVID–19 pandemic on patients diagnosed with AUD	753	329	F1
46	Giménez-Palomo et al. 2024	Spain	ED	January to December 2019	January to December 2020	Consultation trends of adults to the Psychiatric ED to one of the main third-level hospitals of Barcelona	4814	4007	F1-F9
47	Gómez-Ramiro et al. 2021	Spain	ED, inpatient	December 2019 to March 2020	March to June 2020	Psychiatric emergency attendances to the Hospital Clinic of Barcelona, admissions from the ED	1208	750	F0-F9
48	Goldschmidt et al. 2023	Germany	ED, inpatient	March to May 2019	March to May 2020	Psychiatric emergency presentations and admissions during the first wave of COVID–19 at a Berlin psychiatric hospital	894	813	F2, F3, F6
49	Golubovic et al. 2022	Serbia	Inpatient	May to August 2019	May to August 2020	Adult psychiatric inpatients admitted at University Clinic Center	96	103	F0, F2, F3, F4, F6, F7
50	Goncalves-Pinho et al. 2020	Portugal	ED	March to May 2019	March to May 2020	Visits to the ED of the University Hospital Center	1633	780	F2, F3, F4, F6, F9
51	Hamlin et al. 2022	Sweden	ED, inpatient	March to June 2018 and 2019	March to June 2020	Changes in the rate and pattern of visits and hospital admissions for psychiatric disorders at a large Swedish hospital	330	290	F1, F2, F3, F4, F6
52	Hansen et al. 2024	New Zealand	Inpatient	January to December 2019	January to December 2020	All hospitalizations in New Zealand with a primary psychiatric diagnosis before and during the pandemic	27,916	27,808	F1-F6
53	Hakansson et al. 2021	Sweden	ED	January to December 2019	March to December 2020	Number of patients seen in two emergency psychiatric facilities	5816	5127	total
54	Holland et al. 2021	USA	ED	March to October 2019	March to October 2020	Changes in US ED visits for mental health conditions from more than 3500 EDs	40,492	40,230	total
55	Irigoyen-Otiñano et al. 2024a	Spain	ED, inpatient, outpatient	January to March 2020	March to June 2020	Differences in emergency psychiatric visits before and during the COVID–19 lockdown in Lleida, Spain	697	902	F1-F5
56	Irigoyen-Otiñano et al. 2024b	Spain	ED, inpatient	January to March 2020	March to June 2020	Emergency care and continuity of care for patients with substance use disorders at the Hospital Universitario Santa María de Lérida	206	317	F1
57	Jagadheesan et al. 2021 a	Australia	Inpatient	March to September 2019	March to September 2020	Patients admitted to inpatients units of a large mental health network in Melbourne	1487	1307	F0, F1, F2, F3, F4, F6
58	Jagadheesan et al. 2021 b	Australia	ED	March to September 2019	March to September 2020	The rate of ED presentations at a public mental health service of the Royal Melbourne Hospital	467	451	F2-F3
59	Jahlan et al. 2022	Saudi-Arabia	Outpatient	March to June 2019	March to June 2019	Effects of COVID–19 pandemic lockdown on the utilization of mental health services in King Abdulaziz Medical City in Jeddah	141	69	total
60	Jones et al. 2023	USA	Telemedicine medication prescription	September 2018 to February 2020	September 2019 to February 2021	Examine the association of the Receipt of telehealth services and medications for OUD with fatal drug overdoses before and during the pandemic	11,894	22,635	F1
61	Jones et al. 2024	Australia	ED	January 2018 to March 2020	March to June 2020	Effect of the pandemic on mental health ED presentations by comparing observed presentation numbers to predictions from pre-pandemic data	98,995	14,983	F2-F5
62	Joo et al. 2022	South Korea	Inpatient, outpatient	February to August 2019	February to August 2020	Investigate changes in the use of psychiatric services during the early phase of the COVID–19 pandemic at the nationwide level	10,792,854	10,449,486	F2-F4
63	Kim et al. 2023b	USA	Inpatient	March to July 2019	March to July 2020	Characterize and compare inpatient psychiatric admissions in West Texas before and during the initial months of the pandemic	674	718	total
64	Kim et al. 2023a	South Korea	Inpatient, outpatient	January to December 2019	January to December 2020	Impact of the coronavirus disease 2019 (COVID–19) pandemic on mental health service utilization	232,858	196,911	total
65	Lee et al. 2020	China	Inpatient, outpatient, telemedicine	November 2019 to January 2020	February to April 2020	Psychogeriatric admissions, number of outpatient services in a major psychiatric unit in Hong Kong	1632	1685	F0, F1, F2, F3, F4, F7
66	Lee et al. 2022	Korea	ED	January to June 2019	January to June 2020	Psychiatric patients visiting 402 nationwide EDs	88,520	73,281	F2-F3
67	Lee et al. 2023	USA	Outpatient	January to December 2019	January to December 2020	Care delivery and patient characteristics in outpatient mental health clinics within an academic health system	57,629	61,766	total
68	Lee et al. 2024	USA	Outpatient	March to December 2019	March to December 2020	Mental healthcare utilization patterns among individuals with pre-existing mental disorder	130,749	133,272	total
69	Leonhardt et al. 2024	Norway	Mixed (inpatient, outpatient)	January to December 2019	January to December 2020	Frequency of substance-induced psychosis (SIP) during COVID–19 among individuals	621	545	F1
70	Li et al. 2023	China	Outpatient	January to December 2019	January to December 2020	Effect of the COVID–19 pandemic on the use of serious mental illness (SMI)-related outpatient services in Ningbo, China	235,667	237,889	F2-F3
71	Lieber et al. 2024	Sweden	Inpatient, outpatient, medication prescriptions	January to December 2019	January to December 2020	Impact of the COVID–19 pandemic on mental health in Sweden in terms of mental health service utilization	57930	57,308	F1-F4
72	Lin et al. 2023	USA	Outpatient	March 2019 to February 2020	March 2020 to February 2021	Patients enrolled in a large academic hospital’s outpatient psychiatry programs	8030	8224	total
73	Luo et al. 2024	France Germany Italy United Kingdom South Korea USA	Medication prescriptions	April to December 2019	April to December 2020	Examine the changes in psychotropic drug prescribing during the pandemic among people with depressive and anxiety disorders in six countries	omitted for clarity due to multiple countries		total
74	Ludwig et al. 2022	Germany	Medication prescription	January to December 2019	January to December 2020	Verordnungen von Psychopharmaka aus dem Arzneiverordnungs-Report, Verodnungsvolumen in DDD	2,069 Mrd.	2,141 Mrd.	total
75	Mangiapane et al. 2022	Germany	Outpatient	January to May 2019	January to May 2020	Changes in utilization in psychiatry – data from the German Trend Report	3,896,892	3796335	total
76	McAndrew et al. 2021	Ireland	ED	March to May 2019	March to May 2020	Number of psychiatry presentations to the ED of a large academic teaching hospital	176	139	F0-F6
77	McDowell et al. 2021	USA	ED	March to May 2019	March to May 2020	ED psychiatric presentation in a large tertiary care hospital	561	358	total
78	McKee et al. 2021	Canada	Medication prescription	March to May 2019	March to May 2020	Long-acting injectable antipsychotics prescribing practices during the pandemic from Canadian retail pharmacies	4303	4518	total
79	Mehrabadi et al. 2024	USA	Inpatient	March to October 2019	March to October 2020	Influence of the COVID–19 pandemic, including public health measures, on new onset mental health diagnoses	32,647	32825	total
80	Minian et al. 2021	Canada	Outpatient	January 2017 to March 2020	March to December 2020	Pandemic related changes in enrollments in a smoking cessation program (nicotine dependence)	51,275	9098	F17.2
81	Molina et al. 2022	USA	ED	January 2012 to December 2019	January to December 2020	Examine natural trends in ED during an extended period and assess whether these trends differed during COVID–19	Total population: 8967 RR values were taken directly from the original study		total
82	Montalbani et al. 2021	Italy	ED, inpatient	January to March 2020	March to May 2020	Psychiatric consultation in the ED of an Italian hospital, admissions from the ED	133	58	F2, F3, F4, F6
83	Moreno-Martos et al. 2024	Norway Sweden	Inpatient, medication prescription	January 2018 to February 2020	March 2020 March to December 2020	Impact of the early COVID–19 pandemic on mental health-related care in Norway and Sweden	Total population: 4,232,459 (Norway) 8 180 542 (Sweden) RR values were taken directly from the original study		F2-F4
84	Mukadam et al. 2021	United Kingdom	ED	January to December 2019	January to December 2020	Acute mental health presentations to three mental health liaison teams and two mental healthcare centers	9665	8296	total
85	Muştucu et al. 2023	Turkey	ED	March to September 2019	March to September 2020	Number and characteristics of emergency psychiatric consultations to Emergency Department of Bursa Uludağ University	357	367	F0-F4
86	Nejati et al. 2021	Canada	Inpatient	January to March 2020	March to June 2020	Admissions to an urban acute care psychiatric centre	190	185	total
87	Palzes et al. 2022	USA	Mixed (inpatient, outpatient, telemedicine)	March to December 2019	March to December 2020	Impact of the COVID– 19 pandemic on alcohol treatment utilization from Kaiser Permanente of Northern California	15606	13302	F1
88	Panariello et al. 2021	Italy	Inpatient	February to April 2019	February to April 2020	Variation in psychiatric hospitalization rates at the “Maggiore” Hospital in Bologna	81	47	total
89	Patel et al. 2021	United Kingdom	Outpatient, telemedicine	March 2019 to March 2020	March to May 2020	Clinical contacts with mental healthcare professionals (South London and Maudsley NHS Foundation Trust)	216878	207530	total
90	Perozziello et al. 2023	France	Inpatient, ED	January to December 2019	January to December 2020	The medium and long-term impact of the COVID–19 pandemic on the use of mental health services	13586	11189	F1-F6
91	Pignon et al. 2020	France	ED, inpatient	March to April 2019	March to April 2020	Number and characteristics of psychiatric consultations in three emergency services, admissions from the ED	1224	553	F1, F2, F3, F4, F6
92	Pikkel Igal et al. 2021	Israel	ED	March to April 2018 and 2019	March to April 2020	Psychiatric visits to the ED in a Health Care Center	1308	462	F1, F2, F3, F4, F6
93	Qamruddin et al. 2022	United Arab Emirates	Inpatient	March to June 2019	March to June 2020	Effect of the pandemic on the socio-demographic and clinical profiles of patients who were admitted to a tertiary psychiatric hospital in the UAE	189	337	F1-F3
94	Rachamin et al. 2023	Switzerland	Inpatient, outpatient, telemedicine, medication	January to December 2019	January to December 2020	Impact of the COVID–19 pandemic on the utilization of inpatient and outpatient mental healthcare in Switzerland	8446571	8513022	total
95	Romer et al. 2021	Denmark	Inpatient	January to December 2019	January to December 2020	Number of psychiatric admissions (records from hospitals and Emergency Medical Services)	14021	13749	F0-F9
96	Ramadan et al. 2022	Saudi-arabia	ED	January 2018 to December 2019	January 2020 to March 2021	Trends in mental health disorder ED visits – multi center data	298	705	F2-F4
97	Raventos et al. 2022	Spain	Outpatient	March 2018 to February 2020	March to June 2020	Incidence of anxiety and depressive disorder – primary care records in Catalonia	153450	27030	F3-F4
98	Rice et al. 2025	USA	Outpatient, telemedicine, medication prescription	March 2019 to March 2020	March to December 2020	Mental health care delivered to rural and urban Department of Veterans Affairs patients across the COVID–19 pandemic	2720029	2772922	total
99	Ross et al. 2023	USA	inpatient	October 2019 to February 2020	March to October 2020	Trends concerning admissions due to psychosis in the ED before and after the beginning of the COVID–19 pandemic	90	277	total
100	Rugova et al. 2024	Kosova	Medication prescription	January to December 2019	January to December 2020	Mental health situation in Kosovo and the impact of the COVID–19 pandemic	16,7 per 1000 inhabitants	27,7 per 1000 inhabitants	antidepressants anxiolytics
101	Russolillo et al. 2024	Canada	Inpatient	March to December 2019	March to December 2020	Admissions for psychiatric-related hospitalizations before and during the COVID–19 pandemic in Vancouver	472	467	F1-F4, F6
102	Salamah et al. 2024	United Arab Emirates	Inpatient	January to December 2019	January to December 2020	Impact of COVID–19 on sociodemographic trends and diagnostic profiles of outpatient attendees at Rashid Hospital, Dubai	627	468	F2-F4
103	Sanchez-Guarnido et al. 2022	Spain	Outpatient, telemedicine	January to March 2020	March to May 2020	Records of 270 service users of fifteen outpatient mental health services across Spain			Total
104	Savić et al. 2022	Croatia	Inpatient, outpatient, ED	January to December 2019	January to December 2020	Changes in out- and in-patient services utilization in the largest Croatian psychiatric institution	18721	15899	Total
105	Seifert et al. 2021	Germany	ED, inpatient	March to May 2019	March to May 2020	Psychiatric ED visits in an academic teaching hospital, admissions from the ED	476	374	F1, F2, F3, F4, F6
106	Seo et al. 2021	South Korea	Outpatient	October to December 2019	March to May 2020	Mental health service use in a tertiary hospital	14053	12119	F0, F2, F3, F4
107	Silva-Valencia et al. 2024	Argentina Australia Canada China Norway Peru Singapore, USA Sweden	Outpatient, telemedicine	January 2018 to February 2020	April 2020 to February 2021	Primary care visit trends related to mental health conditions in Argentina, Australia, Canada, China, Norway, Peru, Singapore, Sweden, and the USA	omitted for clarity due to multiple countries		Total
108	Simkin et al. 2022	United Kingdom	Outpatient	March to July 2019	March to July 2020	Analyzing data on routine referrals to mental health services for older adults services of a large mental health trust	1455	536	F0-F3
109	Simpson et al. 2021	USA	ED	January to December 2019	January to December 2020	Change in hospitalization rates among three psychiatric emergency services	23012	21552	Total
110	Sobetzko et al. 2021	Germany	ED, inpatient	March to May 2019	March to May 2020	Presentations to the ED in a hospital – single center data, admissions from the ED	374	387	F1, F2, F3, F4, F6
111	Stein et al. 2020	Italy	ED	January to May 2019	January to May 2020	ED visits for mental health-related conditions at a University Hospital	819	625	Total
112	Sweet et al. 2022	USA	Outpatient, telemedicine	November 2019 to February 2020	March 2020 to February 2021	Use of tele-mental health services using a large health system database	6283729	22338880	F3-F4
113	Villarreal-Zegarra et al. 2023	Peru	Outpatient	March 2019	March 2020	number of users receiving care during a given month at all Community Mental Health Centres	23660	20915	F2, F3, F4
114	Visser et al. 2025	Netherlands	Medication prescription	March 2019 to March 2020	March 2020 to March 2022	Trends and dynamics of out-patient prescribing of psychotropic medications during the COVID–19 pandemic in the Netherlands	482742474	1021813377	Total
115	Vukićević et al. 2025	Croatia	Inpatient	March 2019 to February 2020	March 2020 to March 2021	Number of psychiatric hospitalizations in a tertiary hospital in South Croatia	1501	1084	F0-F7
116	Vukojevic et al. 2021	Croatia	Inpatient	February to November 2019	February to November 2020	Number of emergency psychiatric admissions – single center study	3416	2371	F0, F1, F2, F3, F6, F7
117	Wang et al. 2024	USA	ED	May to December 2019	May to December 2020	Emergency department (ED) utilization from 20 EDs across a large Midwest	32320	38711	F1-F4
118	Warwicker et al. 2023	Malta	Inpatient	January to December 2019	January to December 2020	Impact of the pandemic on inpatient mental health, by reviewing the clinical parameters of all psychiatric admissions to Mount Carmel Hospital	1348	1378	F0-F7
119	Wettstein et al. 2022	South Africa	Inpatient, outpatient	March to May 2019	March to May 2020	Hospital admissions and outpatient consultations from a large private sector medical insurance scheme	475998	473985	F0-F4
120	Williams et al. 2020	United Kingdom	Outpatient, medication prescription	March to May 2019	March to May 2020	Primary care data on common mental health problems and SSRI medication prescriptions from 47 general practices	2885	1522	Total
121	Wullschleger et al. 2023	Switzerland	Inpatient	January to December 2019	January to December 2020	Patients admitted to the Department of Psychiatry of the Geneva University Hospital	15263	13533	Total
122	Yalcin et al. 2021	Turkey	ED	April to June 2019	March to May 2020	Psychiatric ED visits in a mental health epicenter	3201	2638	F0-F9
123	Yang et al. 2022	China	Inpatient	January to April 2019	January to April 2020	Changes in hospitalization in a tertiary teaching hospital (mental and behavioral disorders)	200	29	Total
124	Ying et al. 2023	Canada	Medication dispensing	December 2018 to January 2020	February 2020 to March 2021	changes in dispensing patterns of mental health medications in Alberta, Canada	712151	776301	Total
125	Zaki et al. 2022	Australia	Inpatient, medication prescription	January to December 2019	January to December 2020	Investigate whether COVID–19 has led to increased usage of benzodiazepines in acute psychiatric settings	16816	22242	F1-F4, F6 benzodiazepines
126	Zhang et al. 2022	USA	Outpatient, telemedicine, medication prescriptions	March to May 2019	March to May 2020	Trends in patients treated for mental health disorders and adverse events during Pandemic-related health care transformation	891830	1289338	Total
127	Zhang et al. 2023	China	ED	January to December 2019	January to December 2020	Changes in the frequency or patients’ demographics of visiting the PED in the largest psychiatric hospital in China	136	241	F4
128	Zielasek et al. 2021	Germany	Inpatient	March to May 2019	March to May 2020	Routine data of all inpatient and day-patient admission cases in nine psychiatric hospitals of the Landschaftsverband Rheinland	14067	10545	F1, F2, F3, F4, F6

### Study observation periods

We selected 2019 as the reference year for studies comparing multiple years with 2020 to ensure consistency and enhance comparability across studies. However, studies with different comparison periods were also included. Whenever possible, we compared the same periods before and during the pandemic to minimize the influence of seasonal variations on service utilization. An overview of the observation periods for each study can be found in the Supplementary Material in Table S5. For analysis we separated studies that examined the initial phase of the pandemic outbreak in 2020 (short term), using a cut-off of 8 months, from those that investigated longer periods, such as the entire year 2020 or subsequent years (long term).

### COVID-19 containment and health index

To show the country-specific degree of COVID-19 containment measures for the respective periods of the included studies, we added the COVID-19 Containment and Health Index (CCHI) [16] to Supplementary Table 2. This index “is a composite measure based on 13 policy response indicators, including school closures, workplace closures, travel bans, testing policy, contact tracing, face coverings, and vaccine policy, rescaled to a value from 0 to 100 (100 = strictest)” [16].

### Quality assessment

The quality of the included studies was assessed using a modified version of the Newcastle-Ottawa Scale (NOS) for cohort studies [17]. This scale evaluates studies across three main categories—Selection, Comparability, and Outcome—comprising eight subcategories in total. A maximum of seven stars could be awarded, with a star (“☆”) indicating that the criterion was met. If a criterion was not met, it was marked with a “/” symbol (for details, see Table S1 and S2 in the Supplementary Materials).

### Level of observation

To estimate the quantitative changes in service utilization during the pandemic more reliably, we categorized the studies into three groups based on the varying data foundations:

- **Category A studies:** Complete or nearly complete surveys of a larger region, state, or country (e.g., regional health register data, health insurance data, or healthcare data from main regional community health providers).
- **Category B studies:** Samples covering several departments or clinics that do not represent the main or only healthcare provider within a defined larger region or cover the complete or nearly complete population of such a region (e.g., “13 Germany-based hospitals”).
- **Category C studies:** Data from individual clinics or departments (e.g., “Geneva University Medical Center”).

### Data analysis

In our meta-analysis, the natural logarithm of the rate ratio (ln(RR)) was used as the effect size for statistical computations. Random-effects models were employed to estimate summary effect sizes, accounting for both within-study and between-study variability. After conducting the analyses, the ln(RR) values were exponentiated to obtain the rate ratios (RR), which are presented in all figures and tables for ease of interpretation. All analyses were performed using RStudio (Version 2023.09.1) with R (Version 4.4.1). Heterogeneity was assessed using the *I* ^2^ statistic from Cochran’s *Q* and *τ* ^2^ calculated with the restricted maximum likelihood (REML) method. To assess publication bias, funnel plots were generated for each setting (Supplementary Figure S2a–e).

## Results

**Figure 1.**
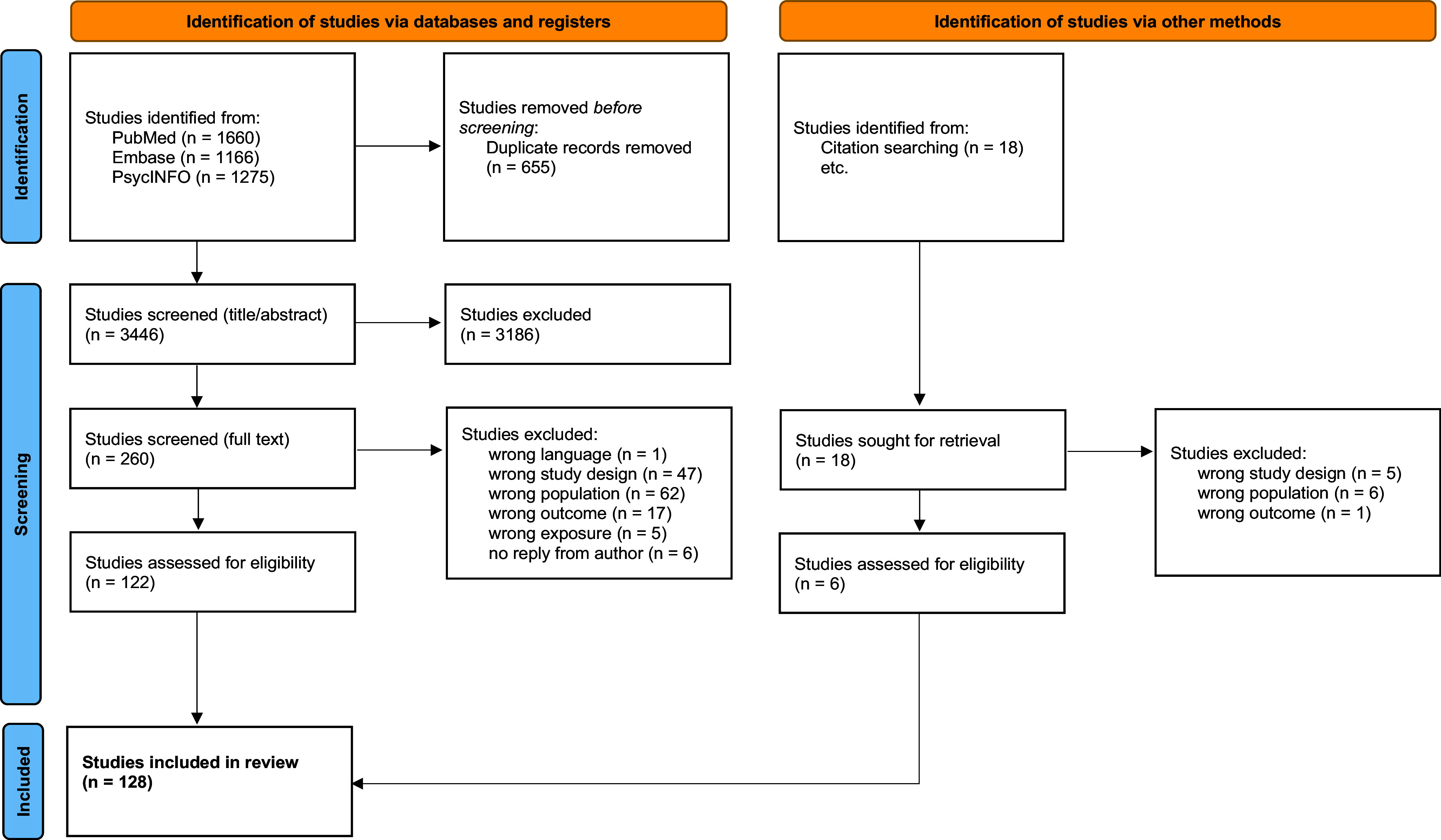
PRISMA flow diagram of study selection.

Initially, 4101 records were retrieved and 655 duplicates were excluded. After title/abstract screening, 260 studies remained for full-text screening. Following the full-text screening, 122 studies from the database search were included. The interrater-agreement showed a Cohen’s Kappa of 0.74. Citation screening identified 18 additional studies, of which 6 were selected for inclusion, resulting in a total of 128 studies included in the review [13]. Figure 1 illustrates the PRISMA flow diagram, outlining the steps involved in the screening and selection process.

Four studies [18–21] report results from multiple countries. The following countries are represented in our review: the United States [19, 21–43] with 25 studies, Italy [19, 44–57] with 15 studies, Germany [19, 58–69] with 13 studies, the United Kingdom [19, 70–78] with 10 studies, Spain [79–86] with 8 studies, Canada [21, 87–93] and Sweden [18, 20, 21, 94–97] with 7 studies each, South Korea [19, 98–101], Australia [21, 102–105] and China [21, 106–109] with 5 studies each, France [19, 110–112] with 4 studies, Croatia [113–115], Netherlands [18, 116, 117], Switzerland [118–120] and Turkey [121–123] with 3 studies each, Malta [124, 125], Portugal [126, 127] and Saudi Arabia [128, 129] with 2 studies each. Additionally, one study originated from each of the following countries: Belgium [130], Denmark [131], Ireland [132], Israel [133], Serbia [134], South Africa [135], Argentina [21], Austria [136], Hungary [137], Japan [138], Kosovo [139], Latvia [18], New Zealand [140] and Singapore [21] (Supplementary Table S3). All continents were represented. However, the majority of studies came from the European Region (86 studies), followed by 35 studies from the Americas, 18 from the Western Pacific, 4 from the Eastern Mediterranean Region, and 1 from the Africa Region (Supplementary Figure S1), according to World Health Organization (WHO) specifications [141]. According to the World Bank classification, only high-income and upper-middle-income economies were represented (Supplementary Table S4). Detailed data are provided in Table 1. Furthermore, Supplementary Table 2 presents the individual index values of the COVID-19 Containment and Health Index for each studies region and the corresponding comparison period during the COVID-19 pandemic.

Most studies included in our analysis compared periods from 2019 with similar periods during the pandemic. The length of comparison periods varied, with some studies examining only a few months, mainly during COVID-19 high-incidence or lockdown periods, while others covered an entire year. Some studies also compared non-equivalent periods within the same year (e.g., the end of 2019 to the beginning of 2020) (Supplementary Table S5). Supplementary Table 2 outlines the distribution of the studies across different settings and the respective periods during the pandemic. In some cases (e.g. 58, 59, 94, 122), it was possible to extract and analyze data from both the short-term and long-term comparison periods and data for these extended time periods.

Sixty-four studies examined inpatient services. Forty-three studies addressed ED services, and 43 studies covered outpatient services. Twenty studies focused on psychotropic medication and 16 studies on telemedicine services (Table 1).

Four studies, listed in Table 1 and Supplementary Table 2 were excluded from the meta-analysis either due to insufficient comparability [60, 91] or because the data combined multiple service settings, preventing a disaggregated analysis of individual settings [40, 142].

Supplementary Figure S7 shows the classification of studies into one of the three levels of observation categories, as described in the Methods section. We performed separate calculations for the meta-analysis, including only the most representative category A and B studies and all three categories (Supplementary Table 2). In the following, we outline results for studies that belong to level of observation categories A or B, and we report short-term (first 8 month of the pandemic) separately from long-term comparison periods. The results for category C studies are presented in Supplementary Table 2. The corresponding forest plots for category C, as well as the forest plots for the long-term observations, can be found in the Supplementary Material (Figure S8a-d).

**Figure 2.**
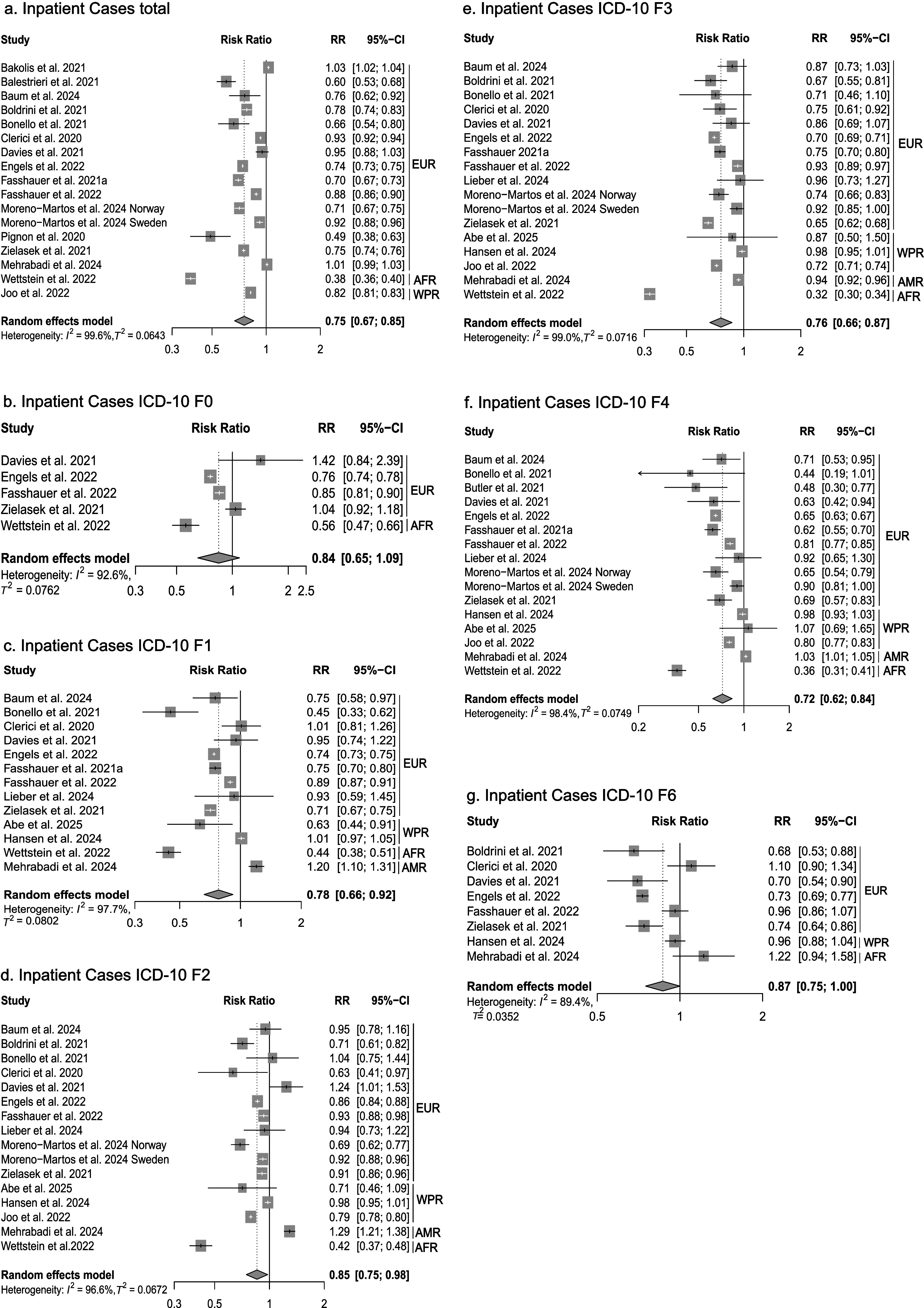
Forest plots of inpatient services utilization. AFR = African Region, AMR = Americas, EUR = European Region, WPR = Western Pacific Region.

**Figure 3.**
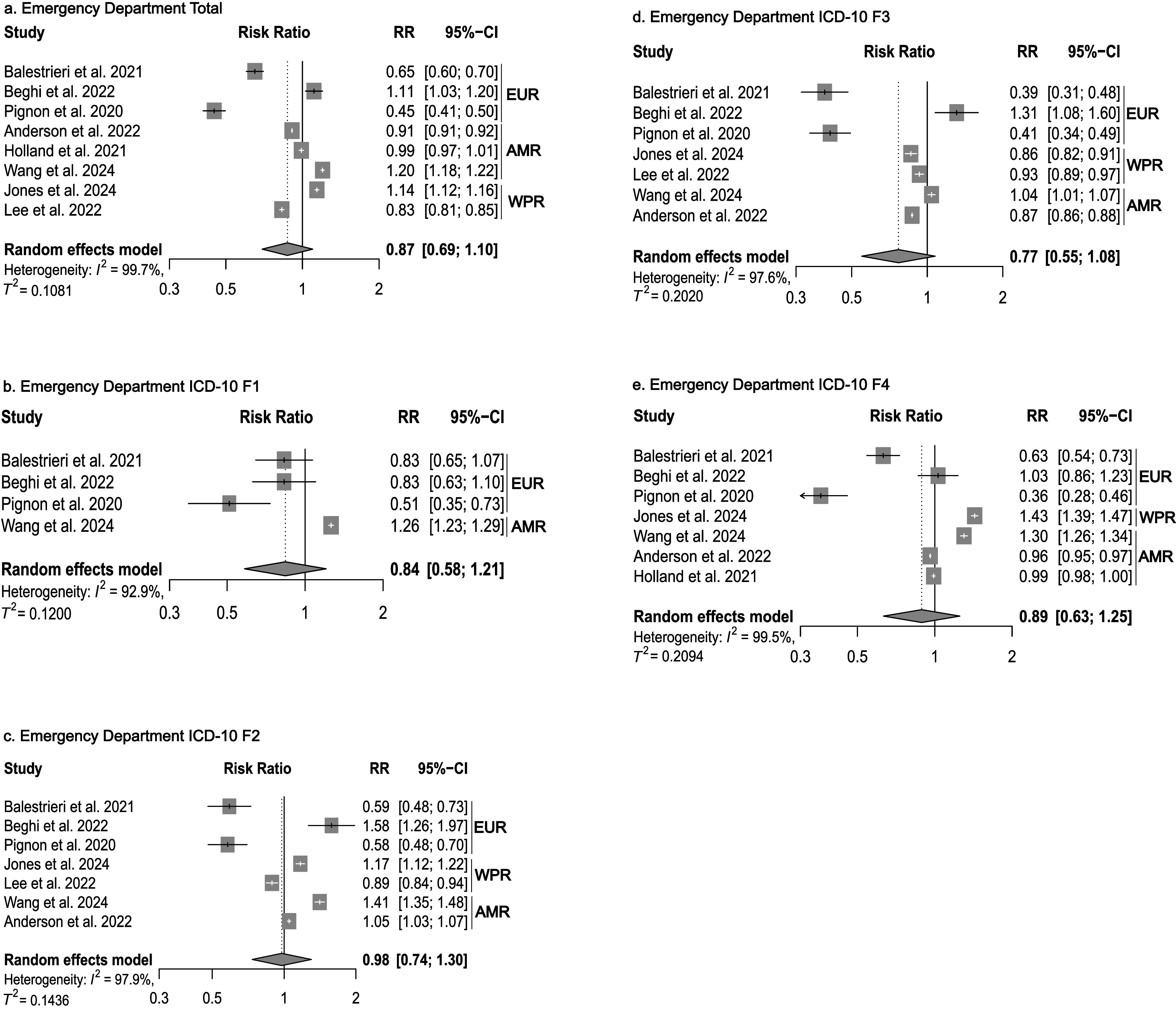
Forest plots of emergency department service utilization. AFR = African Region, AMR = Americas, EUR = European Region, WPR = Western Pacific Region.

**Figure 4.**
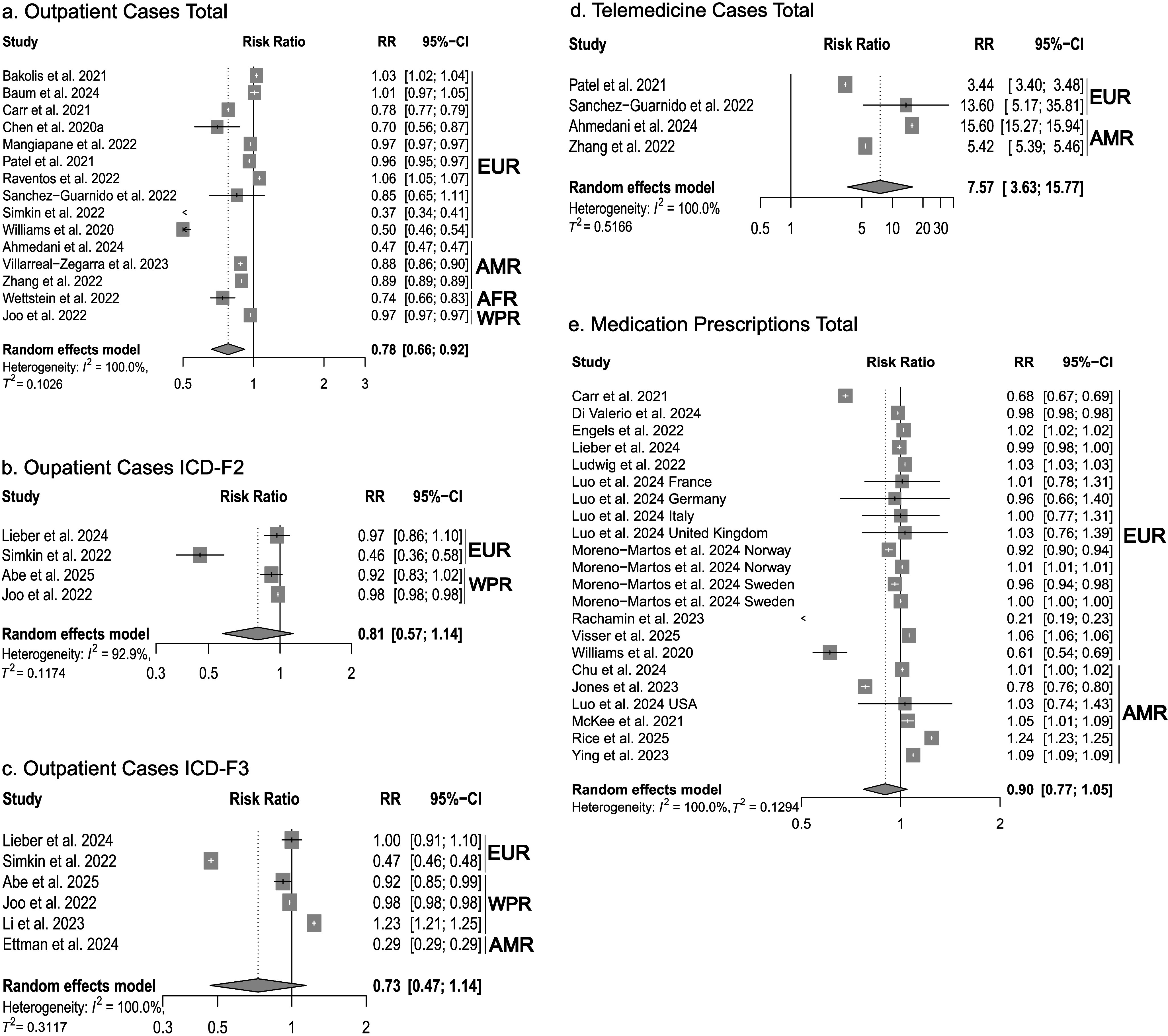
Forest plots of health care service utilization. (a–c) outpatient services, (d) telemedicine cases, (e) medication prescriptions. AFR = African Region, AMR = Americas, EUR = European Region, WPR = Western Pacific Region.

For the initial phase of the pandemic, all settings, except for telemedicine, showed a decrease in service utilization (Supplementary Table 2). High *τ* ^2^ values, particularly for telemedicine (*τ* ^2^: 0.51), reflect substantial between-study variability. We performed detailed subgroup analyses based on ICD-10 F-diagnosis disease categories for inpatient (Figure 2), emergency department (Figure 3), and partly outpatient service (Figure 4) as a sufficient number of Category A or B studies were only found for these settings.

### Inpatient services utilization

First, we analyzed changes in inpatient service utilization. During the initial phase of the pandemic, a significant decrease in utilization was observed across all diagnosis groups (RR: 0.75, 95% CI: 0.67 to 0.85, *n* = 16 studies, *I* ^2^ = 99.6%, Tau^2^ = 0.064) (Figure 2a). The analysis of diagnostic subgroups showed significant decreases in utilization for substance-related disorders (ICD-10 F1) (RR: 0.78, 95% CI: 0.66 to 0.92, *I* ^2^ = 97.7), schizophrenia, schizotypal, delusional, and other non-mood psychotic disorders (ICD-10 F2) (RR: 0.85, 95% CI: 0.75 to 0.98, *I* ^2^ = 96.6%), mood disorders (ICD-10 F3) (RR: 0.76, 95% CI: 0.66 to 0.87, *I* ^2^ = 99.0%), anxiety, dissociative, stress-related, and somatoform mental disorders (ICD-10 F4) (RR:0.72, 95% CI: 0.62 to 0.84, *I* ^2^ = 98.4%), and personality disorders (ICD-10 F6) (RR: 0.87, 95% CI: 0.75 to 1, *I* ^2^ = 89.4%) (Figure 2c–g). No significant changes were observed for organic mental disorders (ICD-10 F0) (Figure 2b). For the long-term comparison period, a less pronounced but statistically significant decline in inpatient utilization was observed (RR: 0.93, 95% CI: 0.89 to 0.98, *I* ^2^ = 99.8%) (Supplementary Table 2).

### Emergency department service utilization

Next, we examined ED utilization for mental disorders. The meta-analytic model showed a reduction of RR: 0.87 (95% CI: 0.69 to 1.10, *n* = 8 studies, *I* ^2^ = 99.7%, Tau^2^ = 0.108) across all diagnosis groups for the initial phase of the pandemic (Figure 3a). The analysis for diagnosis subgroups (ICD-10 F1, F2, F3, and F4) showed no significant change (Figure 3b–e). For the long-term comparison periods, a non-significant, slight increase in the utilization of mental health ED services was observed, with substantial heterogeneity in primary studies (RR: 1.18, 95% CI: 0.66 to 2.09) (Supplementary Table 2).

### Outpatient services, telemedicine, medication

Finally, we examined changes in outpatient and telemedicine service utilization and psychotropic medication prescriptions. Due to the small number of studies, no diagnostic subgroup analyses were performed for telemedicine and medication prescriptions. During the initial phase of the pandemic, we observed a significant decrease in outpatient service utilization (RR: 0.78, 95% CI: 0.66 to 0.92, *n* = 15 studies, *I* ^2^ = 100%, Tau^2^ = 0.102) (Figure 4a). The analysis for diagnosis subgroups (ICD-10 F2 and F3) in the outpatient setting showed no significant change, whereas meta-analysis showed a significant increase in the utilization of telemedicine (RR: 7.57, 95% CI: 3.63 to 15.77, *n* = 4 studies, *I* ^2^ = 100%, Tau^2^ = 0.516) (Figure 4d) and no significant change in psychotropic medication prescriptions (RR: 0.90, CI: 0.77 to 1.05, *n* = 15 studies, *I* ^2^ = 100%, Tau^2^ = 0.129) (Figure 4e).

For the long-term comparison periods, a significant decrease in the utilization of outpatient services (RR: 0.79, 95% CI: 0.65 to 0.97, *I* ^2^ = 100%), and telemedicine services (RR: 18.38, 95% CI: 3.63 to 93.08, *I* ^2^ = 100%) was observed, whereas medication prescriptions showed no significant change (RR: 0.91, 95% CI: 0.74 to 1.11, *I* ^2^ = 100%).

### Regional differences in service utilization

For some regions, meta-analysis of regional results were possible: For Europe inpatient psychiatric service utilization declined consistently across all ICD-10 *F* groups (*F*1 = RR: 0.78, 95% CI: 0.69 to 0.88, *F*2 = RR: 0.88, 95% CI: 0.80 to 0.97, *F*3 = RR: 0.78, 95% CI: 0.72 to 0.85, *F*4 = RR: 0.71, 95% CI: 0.64 to 0.78), while decreases in the Western Pacific Region were smaller and non-significant (*F*2 = RR: 0.86, 95% CI: 0.71 to 1.03, *F*3 = RR: 0.84, 95% CI: 0.66 to 1.07, *F*4 = RR: 0.90, 95% CI: 0.76 to 1.07). For the ED setting, Europe showed significant reductions only for organic mental disorders (*F*1 = RR: 0.72, 95% CI: 0.54–0.97). Studies from the Americas showed stable or slightly increased utilization (total = RR: 1.03, 95% CI: 0.87 to 1.21). Corresponding forest plots are in Supplementary Figure S3.

## Discussion

This systematic review and meta-analysis of MHS utilization demonstrates significant changes during the COVID-19 pandemic compared to the pre-pandemic period. The most prominent change is a significant decrease in inpatient service utilization during the first month of the pandemic. In contrast, reductions in ED, outpatient services, and psychotropic medication utilization were less pronounced. The analysis of ED studies showed the importance of relying on representative samples, as the Category C studies showed significant reductions, whereas the analysis of the more representative Category A and B studies showed (for all diagnostic groups together) no significant change, even for the initial month of the pandemic. For the long-term observations, reductions in MHS service utilization were smaller, which might reflect an adaptation of patients and systems seeking to balance infection protection with the need for services. The introduction of telemedicine and modifications to clinical practices likely contributed to this recovery. Overall, the reductions in the initial period seemed to be more pronounced in Europe, as meta-analyses of studies from other world regions, like the Western Pacific Region or the Americas, did not indicate significant changes.

For the initial period of the pandemic, the analyses showed differential effects depending on subgroups of mental disorders (according to ICD-10). Significant reductions in inpatient care were noted for substance use disorders (ICD-10 F1), affective disorders (F3), neurotic, stress-related, and somatoform disorders (F4), and personality disorders (F6), whereas for organic mental disorders (F0) and schizophrenia, schizotypal, and delusional disorders (F2), no significant reductions were observed. Regarding ED utilization, a reduction reaching statistical significance was only observed for substance use disorders (F1).

There are no indications that the prevalence or treatment needs for substance use disorders (ICD-10 F1), affective disorders (ICD-10 F3), and neurotic, stress-related, and somatoform disorders (ICD-10 F4) declined during the pandemic. It is more likely that disruptions in service availability and access, as well as patients’ fears of infection or general policy measures like curfews and so forth led to decrease in inpatient treatment utilization for these patients. As well, it is not known in which regions and to what degree existing flexible healthcare models like assertive community treatment or telemedicine were able to compensate for reduce inpatient or outpatient in-person offerings. In that sense, some mental healthcare systems might have been better equipped to deal with the challenges of the pandemic. However, to our knowledge, no large-scale systematic studies have investigated the effects of the reductions on treatment quality, treatment outcomes, or infection prevention. Therefore, these reductions and the potential harm from delayed treatment cannot be assessed. This underscores the need for more systematic monitoring of mental health system utilization and quality at national and international levels.

In this context, the regional and economic imbalance of the included studies is notable: most were from Europe, but even within the European Union, studies were from only 12 of the 27 member states. 27 countries were classified as high-income economies and seven as upper-middle-income economies. No countries from lower-middle-income or low-income economies were represented in our review. Within Europe, this calls for a harmonized mental health system utilization and quality indicators to become an essential part of the planned European Health Data Space, which would enable more comparable European health policies and their effects. This would allow us to learn from the most successful models.

The remarkable increase in telemedicine service utilization (RR: 7.57) can be seen as an essential adaptation of mental healthcare systems. It would be desirable for these service levels to be maintained beyond the pandemic, as they could help mitigate the shortage of qualified mental healthcare professionals, especially in rural areas, and provide low-barrier, low-stigma access options. However, in this context, the evidence base for telemedicine interventions, especially for long-term treatments for severely ill patients, needs further expansion, and access barriers to digital services need to be taken into consideration [81, 143].

Our findings are consistent with previous studies during times of health crisis and longer-term disasters like the SARS outbreaks in Taiwan and Toronto, the West African Ebola outbreak or following Hurricane Katrina, that were showing significant declines in healthcare utilization for outpatient, inpatient, and emergency services. Those reductions were due to fears of infection and restrictive measures [144, 145, 146] as well as infrastructure loss and system fragmentation [147].

This review has several limitations: It focuses only on the first year of the pandemic. Trends in service utilization may have evolved in subsequent years. Continued monitoring and analysis of service usage in the following years are needed to capture the long-term effects and recovery processes in mental health care. The aforementioned geographic and economic imbalance of the included studies limits the generalizability of the findings to global contexts. Future research should aim for studies from a broader, more representative range of regions to enhance the external validity of the findings. The review was limited to studies identified in three databases and restricted to publications in three languages, thus potentially impacting the comprehensiveness of the review. Furthermore, we analyzed only shifts in utilization, but we could not take into account differential access to services, e.g., to telemedicine due to lack of access to technology or digital literacy. In general, the restriction to quantitative studies limits the interpretation, e.g., with regard to the background of changes and experiences of those affected. This review included only studies on adult MHS. It should be repeated for child and adolescent MHS utilization, as those populations seemed to be especially burdened by the pandemic.

The heterogeneity of study results was high, and care must be taken when interpreting these results. This, in comparison to the meta-analysis of randomized-controlled trials, high heterogeneity was not unexpected, as healthcare system organization (see Supplementary Table S3), regional infection protection policies (see CCHI in Supplementary Table 2), and the impact of COVID-19 varied between countries and world regions. Another contributing factor was the heterogeneity of study designs, and sample sizes, ranging from studies with millions of participants to those with as few as 100. This variability may affect the robustness and comparability of the meta-analytic findings, potentially introducing bias and reducing the reliability of the overall conclusions. Therefore, the effects should not be evaluated in terms of their absolute value, but it should be emphasized that they were observed despite the great heterogeneity in MHS organization and protection measures. Consensus on further methodological standardization for studies of healthcare utilization should be pursued [9].

Overall, our analyses suggest that the COVID-19 pandemic led to substantial shifts in mental healthcare utilization, with increased reliance on telemedicine alongside reductions in inpatient and emergency services. It remains unclear to what degree telemedicine or other flexible care interventions were able to compensate for those reduced services, especially as they can also have significant access barriers. The reductions were likely to have left specific patient populations, such as people with substance use disorders, affective disorders, or neurotic, stress-related, and somatoform disorders, underserved. To prepare MHS better for future public health challenges, better internationally comparable longitudinal mental health system utilization and quality surveillance data are needed. Such data would allow us to learn which care models are able to maintain needs-oriented, high-quality care even during disruptive crises like the COVID-19 pandemic.

## Supporting information

10.1192/j.eurpsy.2025.10119.sm001 Glock et al. supplementary material 1 Glock et al. supplementary material

10.1192/j.eurpsy.2025.10119.sm002 Glock et al. supplementary material 2 Glock et al. supplementary material

## Data Availability

The extracted data will be made available on GitHub once the manuscript is published.

## Supplementary material

The supplementary material for this article can be found at http://doi.org/10.1192/j.eurpsy.2025.10119.
