# Supplementary material for: Utilization of mental health services during the first year of the COVID-19 pandemic – a systematic review and meta-analysis
Source: Eur Psychiatry. 2026 Jan 13;69(1):e10. doi: 10.1192/j.eurpsy.2025.10119 (PMC12816939; doi:10.1192/j.eurpsy.2025.10119)
Supplement: Glock et al. supplementary material 1 — Glock et al. supplementary material [file S0924933825101193sup001.docx]

**Supplementary Material – Table of Contents**

**Table S1:** The Newcastle-Ottawa Scale (NOS) for cohort study (modified)

**Table S2:** Quality assessment score – Assessment per study

**Table S3:** Basic MHS characteristics of countries, in which included studies were conducted

**Figure S1:** Countries of this study by WHO region

**Table S4:** Categorization of countries according to income level

**Table S5:** observation periods per study shown as a bar chart

**Figure S2 a-e:** funnel plots to assess risk of bias for each setting, categories of representativeness: A, B. short-term observation period (cut off: 8 months)

**Table S6:** Definitions of the settings 'inpatient', 'outpatient', 'emergency', 'telemedicine' and 'medication' of the included studies

**Figure S3 a-b:** Forest Plots of Regional differences in psychiatric service utilization during the pandemic

**Table S7:** Categories for Representativeness

**Figure S8 a-d -** Supplementary Forest Plots for short-term (cut-off: 8 months) and long-Term. Category of representativeness: C

**Figure S9:** Search strategies for the three databases

**Table S1** - The Newcastle-Ottawa Scale (NOS) for cohort study (modified)The Newcastle-Ottawa Scale (NOS) for cohort study (modified)

| **The Newcastle-Ottawa Scale (NOS) for cohort study (modified)**  **Website:** http://www.ohri.ca/programs/clinical_epidemiology/oxford.asp | | | |
| --- | --- | --- | --- |
| **Original items** | **Modifications** | **Reasons for modifications/omission** | **Response options** |
| **Selection** | | | |
| **1. Representativeness of the exposed cohort**  1) truly representative of the average _______________ (describe) in the community  2) somewhat representative of the average ______________ in the community  3) selected group of users eg nurses, volunteers  4) no description of the derivation of the cohort | **1. Representativeness of the exposed cohort**  1) truly representative of the review’s target group*  2) somewhat representative of the reviews target group  3) selected group of users eg patients frequenting one institution/emergency department  4) no description of the derivation of the cohort  *patients with pre-existing or newly diagnosed mental disorders (ICD-10, DSM-5), aged 18 years or older (i.e. adult mental healthcare system | not modified/omitted | ☆  ☆  /  / |
| **2. Selection of the non-exposed cohort**  1) drawn from the same community as the exposed cohort  2) drawn from a different source  3) no description of the derivation of the non exposed cohort | **2. Selection of the non-exposed cohort**  1) drawn from the same community/basic population as the exposed cohort (e.g. other time interval but same region/structure of population, groups have same eligibility criteria, the only difference is exposure/non-exposure/point of assessment)  2) drawn from a different source  3) no description of the derivation of the non exposed cohort | not modified/omitted | ☆  /  / |
| **3. Ascertainment of exposure**  1) secure record (eg surgical records)  2) structured interview  3) written self report  4) no description | omitted | The original question is not applicable since all participants of included studies are exposed to the pandemic. | - |
| **4. Demonstration that outcome of interest was not present at start of study**  1) yes  2) no | **4. Demonstration that outcome of interest was not confounded from start of study**  1) yes*  2) no  *****demonstrated if eligibility criteria do not differ except for observation periods | This question was modified to assess the risk of bias arising from differences in eligibility criteria in the pre-pandemic and pandemic periods. | /  ☆ |
| **Comparability** | | | |
| **5. Comparability of cohorts on the basis of the design or analysis**  1) study controls for _____________ (select the most important factor)  2) study controls for any additional factor (This criteria could be modified to indicate specific control for a second important factor.) | **5. Comparability of participants on the basis of the** design or analysis  1) study controls for exposure to Covid-pandemic  2) study controls for any additional factor: exposure to any other epidemic or pandemic infectious disease outbreaks as well as other macrostressors like natural or human-made disasters | The research question was modified to ensure that the comparison periods were as similar as possible in terms of time period or inclusion criteria such as age or gender. | ☆  ☆ |
| **Outcome** | | | |
| **6. Assessment of outcome**  1) independent blind assessment  2) record linkage  3) self-report  4) no description | **6. Assessment of outcome**  1) classified quantitative data (e.g, register or routine data)  2) ad-hoc surveys  3) no description | The question was modified in regard to our inclusion criteria (quantitative data) | ☆  /  / |
| **7. Was follow-up long enough for outcomes to occur**  1) yes (select an adequate follow up period for outcome of interest)  2) no | **7. Does the observation period include a local lockdown or high frequency phase?**  1) yes  2) no | modified | ☆  / |
| **8. Adequacy of follow up of cohorts**  1) complete follow up - all subjects accounted for  2) subjects lost to follow up unlikely to introduce bias - small number lost - >____ % (select an adequate %) follow up, or description provided of those lost)  3) follow up rate < ____% (select an adequate %) and no description of those lost  4) no statement | omitted |  | - |

**Table S2** - Quality assessment score – Assessment per study

| Quality assessment score | Selection | | | | Comparability | Outcome | | |  |  |
| --- | --- | --- | --- | --- | --- | --- | --- | --- | --- | --- |
|  | **1** | **2** | **3** | **4** | **5** | **6** | **7** | **8** | **total** | |
| maximum score a study can achieve | ✩ | ✩ | - | ✩ | ✩ ✩ | ✩ | ✩ | - | **7** | |
| Abe et al. 2025 | ✩ | ✩ | - | ✩ | ✩ | ✩ | ✩ | - | **6** | |
| Adorjan et al. 2021 | ✩ | ✩ | - | ✩ | ✩ | ✩ | ✩ | - | **6** | |
| Ahmedani et al. 2024 | ✩ | ✩ | - | ✩ | ✩ | ✩ | ✩ | - | **6** | |
| Akkaoui et al. 2025 | / | ✩ | - | ✩ | ✩ | ✩ | ✩ | - | **5** | |
| Alves et al. 2021 | / | ✩ | - | ✩ | ✩ | ✩ | ✩ | - | **5** | |
| Ambrosetti et al. 2021 | / | ✩ | - | ✩ | ✩ | ✩ | ✩ | - | **5** | |
| Anderson et al. 2022 | ✩ | ✩ | - | ✩ | ✩ | ✩ | ✩ | - | **6** | |
| Andersson et al. 2022 | / | ✩ | - | ✩ | ✩ | ✩ | ✩ | - | **5** | |
| Bakolis et al. 2021 | ✩ | ✩ | - | ✩ | ✩ | ✩ | ✩ | - | **6** | |
| Balestrieri et al. 2021 | ✩ | ✩ | - | ✩ | ✩ | ✩ | ✩ | - | **6** | |
| Baum et al. 2024 | ✩ | ✩ | - | ✩ | ✩ | ✩ | ✩ | - | **6** | |
| Beghi et al. 2022 | ✩ | ✩ | - | ✩ | ✩ | ✩ | / | - | **5** | |
| Berardelli et al. 2021 | / | ✩ | - | ✩ | ✩ | ✩ | ✩ | - | **5** | |
| Bhagavathula et al. 2024 | ✩ | ✩ | - | ✩ | ✩ | ✩ | ✩ | - | **6** | |
| Boldrini et al. 2021 | ✩ | ✩ | - | ✩ | ✩ | ✩ | ✩ | - | **6** | |
| Bonello et al. 2021 | ✩ | ✩ | - | ✩ | ✩ | ✩ | ✩ | - | **6** | |
| Bruckner et al. 2023 | / | ✩ | - | ✩ | ✩ | ✩ | ✩ | - | **5** | |
| Cafaro et al. 2022 | / | ✩ | - | ✩ | ✩ | ✩ | ✩ | - | **5** | |
| Capuzzi et al. 2020 | / | ✩ | - | ✩ | ✩ | ✩ | ✩ | - | **5** | |
| Carr et al. 2021 | ✩ | ✩ | - | ✩ | ✩ | ✩ | ✩ | - | **6** | |
| Caselli et al. 2023 | / | ✩ | - | ✩ | ✩ | ✩ | ✩ | - | **5** | |
| Chen et al. 2020a | ✩ | ✩ | - | ✩ | ✩ | ✩ | ✩ | - | **6** | |
| Chow et al. 2021 | / | ✩ | - | ✩ | ✩ | ✩ | ✩ | - | **5** | |
| Chu et al. 2024 | ✩ | ✩ | - | ✩ | ✩ | ✩ | ✩ | - | **6** | |
| Clerici et al. 2020 | ✩ | ✩ | - | ✩ | ✩ | ✩ | ✩ | - | **6** | |
| Davies et al. 2021 | ✩ | ✩ | - | ✩ | ✩ | ✩ | ✩ | - | **6** | |
| deDiegoRuiz et al. 2023 | / | ✩ | - | ✩ | ✩ | ✩ | ✩ | - | **5** | |
| Der et al. 2023 | ✩ | ✩ | - | ✩ | ✩ | ✩ | ✩ | - | **6** | |
| Di Lorenzo et al. 2021a | / | ✩ | - | ✩ | ✩ | ✩ | ✩ | - | **5** | |
| Di Lorenzo et al. 2021b | / | ✩ | - | ✩ | ✩ | ✩ | ✩ | - | **5** | |
| Di Valerio et al. 2024 | ✩ | ✩ | - | ✩ | ✩ | ✩ | ✩ | - | **6** | |
| Dindar et al. 2024 | / | ✩ | - | ✩ | ✩ | ✩ | ✩ | - | **5** | |
| Engels et al. 2022 | ✩ | ✩ | - | ✩ | ✩ | ✩ | ✩ | - | **6** | |
| Ettman et al. 2024 | ✩ | ✩ | - | ✩ | ✩ | ✩ | ✩ | - | **6** | |
| Fasshauer et al. 2021a | ✩ | ✩ | - | ✩ | ✩ | ✩ | ✩ | - | **6** | |
| Fasshauer et al. 2021b | ✩ | ✩ | - | ✩ | ✩ | ✩ | ✩ | - | **6** | |
| Fasshauer et al. 2022 | ✩ | ✩ | - | ✩ | ✩ | ✩ | ✩ | - | **6** | |
| Fellinger et al. 2023 | ✩ | ✩ | - | ✩ | ✩ | ✩ | ✩ | - | **6** | |
| Flament et al. 2021 | / | ✩ | - | ✩ | ✩ | ✩ | / | - | **4** | |
| Flodin et al. 2023 | ✩ | ✩ | - | ✩ | ✩ | ✩ | ✩ | - | **6** | |
| Fstkchian et al. 2023 | / | ✩ | - | ✩ | ✩ | ✩ | ✩ | - | **5** | |
| Fu et al. 2024 | / | ✩ | - | ✩ | ✩ | ✩ | ✩ | - | **5** | |
| Fuster-Casanovas et al. 2024 | ✩ | ✩ | - | ✩ | ✩ | ✩ | ✩ | - | **6** | |
| Gajdics et al. 2023 | / | ✩ | - | ✩ | ✩ | ✩ | ✩ | - | **5** | |
| Giménez-Palomo et al. 2024 | / | ✩ | - | ✩ | ✩ | ✩ | ✩ | - | **5** | |
| Goldschmidt et al. 2023 | / | ✩ | - | ✩ | ✩ | ✩ | ✩ | - | **5** | |
| Golubovic et al. 2022 | / | ✩ | - | ✩ | ✩ | ✩ | ✩ | - | **5** | |
| Gómez-Ramiro et al. 2021 | / | ✩ | - | ✩ | ✩ | ✩ | ✩ | - | **5** | |
| Goncalves-Pinho et al. 2020 | / | ✩ | - | ✩ | ✩ | ✩ | ✩ | - | **5** | |
| Hakansson et al. 2021 | / | ✩ | - | ✩ | ✩ | ✩ | ✩ | - | **5** | |
| Hamlin et al. 2022 | / | ✩ | - | ✩ | ✩ | ✩ | ✩ | - | **5** | |
| Hansen et al. 2024 | ✩ | ✩ | - | ✩ | ✩ | ✩ | ✩ | - | **6** | |
| Holland et al. 2021 | ✩ | ✩ | - | ✩ | ✩ | ✩ | ✩ | - | **6** | |
| Irigoyen-Otiñano et al. 2024a | / | ✩ | - | ✩ | ✩ | ✩ | ✩ | - | **5** | |
| Irigoyen-Otiñano et al. 2024b | / | ✩ | - | ✩ | ✩ | ✩ | ✩ | - | **5** | |
| Jagadheesan et al. 2021 a | / | ✩ | - | ✩ | ✩ | ✩ | ✩ | - | **5** | |
| Jagadheesan et al. 2021 b | / | ✩ | - | ✩ | ✩ | ✩ | ✩ | - | **5** | |
| Jahlan et al. 2022 | / | ✩ | - | ✩ | ✩ | ✩ | ✩ | - | **5** | |
| Jones et al. 2023 | ✩ | ✩ | - | ✩ | ✩ | ✩ | ✩ | - | **6** | |
| Jones et al. 2024 | ✩ | ✩ | - | ✩ | ✩ | ✩ | ✩ | - | **6** | |
| Joo et al. 2022 | ✩ | ✩ | - | ✩ | ✩ | ✩ | ✩ | - | **6** | |
| Kim et al. 2023a | ✩ | ✩ | - | ✩ | ✩ | ✩ | ✩ | - | **6** | |
| Kim et al. 2023b | / | ✩ | - | ✩ | ✩ | ✩ | ✩ | - | **5** | |
| Lee et al. 2020 | / | ✩ | - | ✩ | ✩ | ✩ | ✩ | - | **5** | |
| Lee et al. 2022 | ✩ | ✩ | - | ✩ | ✩ | ✩ | ✩ | - | **6** | |
| Lee et al. 2023 | / | ✩ | - | ✩ | ✩ | ✩ | ✩ | - | **5** | |
| Lee et al. 2024 | ✩ | ✩ | - | ✩ | ✩ | ✩ | ✩ | - | **6** | |
| Leonhardt et al. 2024 | ✩ | ✩ | - | ✩ | ✩ | ✩ | ✩ | - | **6** | |
| Li et al. 2023 | ✩ | ✩ | - | ✩ | ✩ | ✩ | ✩ | - | **6** | |
| Lieber et al. 2024 | ✩ | ✩ | - | ✩ | ✩ | ✩ | ✩ | - | **6** | |
| Lin et al. 2023 | / | ✩ | - | ✩ | ✩ | ✩ | ✩ | - | **5** | |
| Ludwig et al. 2022 | ✩ | ✩ | - | ✩ | ✩ | ✩ | ✩ | - | **6** | |
| Luo et al. 2024 (France, UK, Germany, Italy) | ✩ | ✩ | - | ✩ | ✩ | ✩ | ✩ | - | **6** | |
| Luo et al. 2024 (South Korea) | / | ✩ | - | ✩ | ✩ | ✩ | ✩ | - | **5** | |
| Luo et al. 2024 (USA) | ✩ | ✩ | - | ✩ | ✩ | ✩ | ✩ | - | **6** | |
| Mangiapane et al. 2022 | ✩ | ✩ | - | ✩ | ✩ | ✩ | ✩ | - | **6** | |
| McAndrew et al. 2021 | / | ✩ | - | ✩ | ✩ | ✩ | ✩ | - | **5** | |
| McDowell et al. 2021 | / | ✩ | - | ✩ | ✩ | ✩ | ✩ | - | **5** | |
| McKee et al. 2021 | ✩ | ✩ | - | ✩ | ✩ | ✩ | ✩ | - | **6** | |
| Mehrabadi et al. 2024 | ✩ | ✩ | - | ✩ | ✩ | ✩ | ✩ | - | **6** | |
| Minian et al. 2021 | / | ✩ | - | ✩ | ✩ | ✩ | ✩ | - | **5** | |
| Molina et al. 2022 | ✩ | ✩ | - | / | ✩ | ✩ | ✩ | - | **5** | |
| Montalbani et al. 2021 | / | ✩ | - | ✩ | ✩ | ✩ | ✩ | - | **5** | |
| Moreno-Martos et al. 2024 | ✩ | ✩ | - | ✩ | ✩ | ✩ | ✩ | - | **6** | |
| Mukadam et al. 2021 | ✩ | ✩ | - | ✩ | ✩ | ✩ | ✩ | - | **6** | |
| Muştucu et al. 2023 | / | ✩ | - | ✩ | ✩ | ✩ | ✩ | - | **5** | |
| Nejati et al. 2021 | / | ✩ | - | ✩ | ✩ | ✩ | ✩ | - | **5** | |
| Palzes et al. 2022 | ✩ | ✩ | - | ✩ | ✩ | ✩ | ✩ | - | **6** | |
| Panariello et al. 2021 | / | ✩ | - | ✩ | ✩ | ✩ | ✩ | - | **5** | |
| Patel et al. 2021 | ✩ | ✩ | - | ✩ | ✩ | ✩ | ✩ | - | **6** | |
| Perozziello et al. 2023 | / | ✩ | - | ✩ | ✩ | ✩ | ✩ | - | **5** | |
| Pignon et al. 2020 | ✩ | ✩ | - | ✩ | ✩ | ✩ | ✩ | - | **6** | |
| Pikkel Igal et al. 2021 | / | ✩ | - | ✩ | ✩ | ✩ | ✩ | - | **5** | |
| Qamruddin et al. 2022 | / | ✩ | - | ✩ | ✩ | ✩ | ✩ | - | **5** | |
| Rachamin et al. 2023 | ✩ | ✩ | - | ✩ | ✩ | ✩ | ✩ | - | **6** | |
| Ramadan et al. 2022 | ✩ | ✩ | - | ✩ | ✩ | ✩ | ✩ | - | **6** | |
| Raventos et al. 2022 | ✩ | ✩ | - | ✩ | ✩ | ✩ | ✩ | - | **6** | |
| Rice et al. 2025 | ✩ | ✩ | - | ✩ | ✩ | ✩ | ✩ | - | **6** | |
| Romer et al. 2021 | ✩ | ✩ | - | ✩ | ✩ | ✩ | ✩ | - | **6** | |
| Ross et al. 2023 | / | ✩ | - | ✩ | ✩ | ✩ | ✩ | - | **5** | |
| Rugova et al. 2024 | / | ✩ | - | ✩ | ✩ | ✩ | ✩ | - | **5** | |
| Russolillo et al. 2024 | / | ✩ | - | ✩ | ✩ | ✩ | ✩ | - | **5** | |
| Salamah et al. 2024 | / | ✩ | - | ✩ | ✩ | ✩ | ✩ | - | **5** | |
| Sanchez-Guarnido et al. 2022 | ✩ | ✩ | - | ✩ | ✩ | ✩ | ✩ | - | **6** | |
| Savić et al. 2022 | / | ✩ | - | ✩ | ✩ | ✩ | ✩ | - | **5** | |
| Seifert et al. 2021 | / | ✩ | - | ✩ | ✩ | ✩ | ✩ | - | **5** | |
| Seo et al. 2021 | / | ✩ | - | ✩ | ✩ | ✩ | ✩ | - | **5** | |
| Silva-Valencia et al. 2024 | ✩ | ✩ | - | ✩ | ✩ | ✩ | ✩ | - | **6** | |
| Simkin et al. 2022 | ✩ | ✩ | - | ✩ | ✩ | ✩ | ✩ | - | **6** | |
| Simpson et al. 2021 | ✩ | ✩ | - | ✩ | ✩ | ✩ | ✩ | - | **6** | |
| Sobetzko et al. 2021 | / | ✩ | - | ✩ | ✩ | ✩ | ✩ | - | **6** | |
| Stein et al. 2020 | / | ✩ | - | ✩ | ✩ | ✩ | ✩ | - | **6** | |
| Sweet et al. 2022 | ✩ | ✩ | - | ✩ | ✩ | ✩ | ✩ | - | **5** | |
| Villarreal-Zegarra et al. 2023 | ✩ | ✩ | - | ✩ | ✩ | ✩ | ✩ | - | **6** | |
| Visser et al. 2025 | ✩ | ✩ | - | ✩ | ✩ | ✩ | ✩ | - | **6** | |
| Vukićević et al. 2025 | / | ✩ | - | ✩ | ✩ | ✩ | ✩ | - | **5** | |
| Vukojevic et al. 2021 | / | ✩ | - | ✩ | ✩ | ✩ | ✩ | - | **6** | |
| Wang et al. 2024 | ✩ | ✩ | - | ✩ | ✩ | ✩ | ✩ | - | **6** | |
| Warwicker et al. 2023 | / | ✩ | - | ✩ | ✩ | ✩ | ✩ | - | **5** | |
| Wettstein et al. 2022 | ✩ | ✩ | - | ✩ | ✩ | ✩ | ✩ | - | **6** | |
| Williams et al. 2020 | ✩ | ✩ | - | ✩ | ✩ | ✩ | ✩ | - | **6** | |
| Wullschleger et al. 2023 | / | ✩ | - | ✩ | ✩ | ✩ | ✩ | - | **5** | |
| Yalcin et al. 2021 | / | ✩ | - | ✩ | ✩ | ✩ | ✩ | - | **5** | |
| Yang et al. 2022 | / | ✩ | - | ✩ | ✩ | ✩ | ✩ | - | **5** | |
| Ying et al. 2023 | ✩ | ✩ | - | ✩ | ✩ | ✩ | ✩ | - | **6** | |
| Zaki et al. 2022 | / | ✩ | - | ✩ | ✩ | ✩ | ✩ | - | **5** | |
| Zhang et al. 2022 | / | ✩ | - | ✩ | ✩ | ✩ | ✩ | - | **5** | |
| Zhang et al. 2023 | / | ✩ | - | ✩ | ✩ | ✩ | ✩ | - | **5** | |
| Zielasek et al. 2021 | ✩ | ✩ | - | ✩ | ✩ | ✩ | ✩ | - | **6** | |

**Table S3:** Basic MHS characteristics of countries, in which included studies were conducted

| country | studies / results from the respective country (n)* | Mental health: beds in mental hospitals (per 100,000) (year) | Mental health: beds for mental health in general hospitals (per 100,000) **  Last updated:  2019-04-25 | Mental health outpatient facilities (per 100 000) **  Last updated:  2019-04-25 |
| --- | --- | --- | --- | --- |
| Argentina | 1 | 40.566 (2016) | 2.718 | 1.720 |
| Australia | 5 | 7.214 (2015) | 21.761 | No WHO data available |
| Austria | 1 | 26.087 (2015) | 19.485 | 3.457 |
| Belgium | 1 | 98.884 (2013) | 22.706 | No WHO data available |
| Canada | 7 | 11. 092 (2017) | 14.497 | 0.330 |
| China | 5 | 24.288 (2015) | No WHO data available | No WHO data available |
| Croatia | 3 | 75.354 (2016) | 16.501 | 3.093 |
| Denmark | 1 | 51.594 (2016) | No WHO data available | No WHO data available |
| France | 4 | 6.981 (2017) | 22.340 | No WHO data available |
| Germany | 13 | 55.697 (2015) | 80.640 | 0.077 |
| Hungary | 1 | No WHO data available | 85.671 | 8.269 |
| Ireland | 1 | 21.659 (2016) | 21.659 | 3.830 |
| Israel | 1 | 35.228 (2016) | 4.179 | No WHO data available |
| Italy | 15 | No WHO data available | 8.957 | 3.581 |
| Japan | 1 | 196.625 (2016) | 66.147 | 7.223 |
| Kosovo | 1 | No WHO data available | No WHO data available | No WHO data available |
| Latvia | 1 | 102.526 (2015) | No WHO data available | 20.475 |
| Malta | 2 | No WHO data available | No WHO data available | No WHO data available |
| Netherlands | 3 | No WHO data available | No WHO data available | No WHO data available |
| New Zealand | 1 | No WHO data available | 18.788 | No WHO data available |
| Norway | 4 | 74.329 (2016) | No WHO data available | 1.308 |
| Peru | 2 | 3.197 (2016) | 0.405 | 0.166 |
| Portugal | 2 | 5.701 (2016) | 11.825 | 0.701 |
| Saudi-Arabia | 2 | 17.115 (2016) | 0.317 | No WHO data available |
| Serbia | 1 | 41.406 (2017) | 18.325 | No WHO data available |
| Singapore | 1 | 34.867 (2016) | 1.228 | 0.217 |
| South-Africa | 1 | 16.556 (2016) | 4.330 | No WHO data available |
| South-Korea | 5 | 94.217 (2016) | 11.156 | 1.789 |
| Spain | 8 | 28.148 (2015) | 14.307 | 2.045 |
| Sweden | 7 | 31.095 (2016) | No WHO data available | No WHO data available |
| Switzerland | 3 | 75.603 (2015) | 13.810 | No WHO data available |
| Turkey | 3 | 5.158 (2016) | 4.673 | 0.190 |
| United Arab Emirates | 2 | 0.896 (2016) | 2.993 | No WHO data available |
| United Kingdom (UK) | 10 | 23.914 (2017) | 0.000 | No WHO data available |
| United States of America (USA) | 25 | 18.660 (2016) | 11.143 | 0.373 |

* Some studies present results from several countries, so the number of studies does not match the number of countries
** WHO, THE GLOBAL HEALTH OBSERVATORY – Explore a world of health data. [Mental health service availability](https://www.who.int/data/gho/data/themes/topics/GHO/mental-health-service-availability) (28.06.2025)

**Figure S1 -** Countries of this study by WHO region

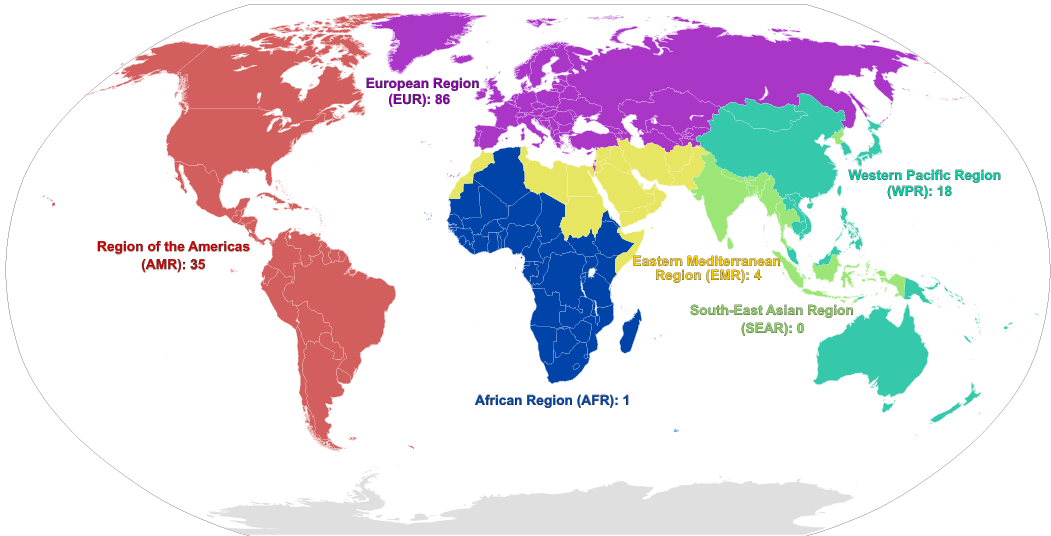


**Table S4** - Categorization of countries according to income level*

| LOW-INCOME ECONOMIES ($1,145 OR LESS) | LOWER-MIDDLE INCOME ECONOMIES ($1,146 TO $4,515) | UPPER-MIDDLE-INCOME ECONOMIES ($4,516 TO $14,005) | HIGH-INCOME ECONOMIES ($14,006 OR MORE) |
| --- | --- | --- | --- |
| - | - | Argentina, China, Kosovo, Peru, Serbia, South Africa, Turkey | Australia, Belgium, Canada, Croatia, Denmark, France, Germany, Hungary, Ireland, Israel, Italy, Japan, South Korea, Latvia, Malta, Netherlands, New Zealand, Norway, Portugal, Saudi Arabia, Singapore, Spain, Sweden, Switzerland, United Arab Emirates, United Kingdom, United States |

*Countries were classified into income groups according to the World Bank (World Bank, 2025)
Literatur: World Bank. (2025). *World Bank country and lending groups*. Retrieved June 22, 2025, from <https://datahelpdesk.worldbank.org/knowledgebase/articles/906519-world-bank-country-and-lending-groups>

**Table S5** - Analysed periods per study shown as a bar chart

= before pandemic

= during pandemic

|  | **2019** | | | | | | | | | | | | **2020** | | | | | | | | | | | | **2021** | | | | | | | | | | | |
| --- | --- | --- | --- | --- | --- | --- | --- | --- | --- | --- | --- | --- | --- | --- | --- | --- | --- | --- | --- | --- | --- | --- | --- | --- | --- | --- | --- | --- | --- | --- | --- | --- | --- | --- | --- | --- |
|  | J | F | M | A | M | J | J | A | S | O | N | D | J | F | M | A | M | J | J | A | S | O | N | D | J | F | M | A | M | J | J | A | S | O | N | D |
| Abe et al. 2025 |  |  |  |  |  |  |  |  |  |  |  |  |  |  |  |  |  |  |  |  |  |  |  |  |  |  |  |  |  |  |  |  |  |  |  |  |
| Adorjan et al. 2021 |  |  |  |  |  |  |  |  |  |  |  |  |  |  |  |  |  |  |  |  |  |  |  |  |  |  |  |  |  |  |  |  |  |  |  |  |
| Ahmedani et al. 2024 |  |  |  |  |  |  |  |  |  |  |  |  |  |  |  |  |  |  |  |  |  |  |  |  |  |  |  |  |  |  |  |  |  |  |  |  |
| Akkaoui et al. 2025 |  |  |  |  |  |  |  |  |  |  |  |  |  |  |  |  |  |  |  |  |  |  |  |  |  |  |  |  |  |  |  |  |  |  |  |  |
| Alves et al. 2021 |  |  |  |  |  |  |  |  |  |  |  |  |  |  |  |  |  |  |  |  |  |  |  |  |  |  |  |  |  |  |  |  |  |  |  |  |
| Ambrosetti et al. 2021 | April to May 2016 |  |  |  |  |  |  |  |  |  |  |  |  |  |  |  |  |  |  |  |  |  |  |  |  |  |  |  |  |  |  |  |  |  |  |  |
| Anderson et al. 2022 |  |  |  |  |  |  |  |  |  |  |  |  |  |  |  |  |  |  |  |  |  |  |  |  |  |  |  |  |  |  |  |  |  |  |  |  |
| Andersson et al. 2022 |  |  |  |  |  |  |  |  |  |  |  |  |  |  |  |  |  |  |  |  |  |  |  |  |  |  |  |  |  |  |  |  |  |  |  |  |
| Bakolis et al. 2021 |  |  |  |  |  |  |  |  |  |  |  |  |  |  |  |  |  |  |  |  |  |  |  |  |  |  |  |  |  |  |  |  |  |  |  |  |
| Balestrieri et al. 2021 |  |  |  |  |  |  |  |  |  |  |  |  |  |  |  |  |  |  |  |  |  |  |  |  |  |  |  |  |  |  |  |  |  |  |  |  |
| Baum et al. 2024 |  |  |  |  |  |  |  |  |  |  |  |  |  |  |  |  |  |  |  |  |  |  |  |  |  |  |  |  |  |  |  |  |  |  |  |  |
| Beghi et al. 2022 |  |  |  |  |  |  |  |  |  |  |  |  |  |  |  |  |  |  |  |  |  |  |  |  |  |  |  |  |  |  |  |  |  |  |  |  |
| Berardelli et al. 2021 |  |  |  |  |  |  |  |  |  |  |  |  |  |  |  |  |  |  |  |  |  |  |  |  |  |  |  |  |  |  |  |  |  |  |  |  |
| Bhagavathula et al. 2024 |  |  |  |  |  |  |  |  |  |  |  |  |  |  |  |  |  |  |  |  |  |  |  |  |  |  |  |  |  |  |  |  |  |  |  |  |
| Bonello et al. 2021 |  |  |  |  |  |  |  |  |  |  |  |  |  |  |  |  |  |  |  |  |  |  |  |  |  |  |  |  |  |  |  |  |  |  |  |  |
| Bruckner et al. 2023 | Jan 2018 |  |  |  |  |  |  |  |  |  |  |  |  |  |  |  |  |  |  |  |  |  |  |  |  |  |  |  |  |  |  |  |  |  |  |  |
| Cafaro et al. 2022 |  |  |  |  |  |  |  |  |  |  |  |  |  |  |  |  |  |  |  |  |  |  |  |  |  |  |  |  |  |  |  |  |  |  |  |  |
| Capuzzi et al. 2020 |  |  |  |  |  |  |  |  |  |  |  |  |  |  |  |  |  |  |  |  |  |  |  |  |  |  |  |  |  |  |  |  |  |  |  |  |
| Caselli et al. 2023 |  |  |  |  |  |  |  |  |  |  |  |  |  |  |  |  |  |  |  |  |  |  |  |  |  |  |  |  |  |  |  |  |  |  |  |  |
| Chen et al. 2020a |  |  |  |  |  |  |  |  |  |  |  |  |  |  |  |  |  |  |  |  |  |  |  |  |  |  |  |  |  |  |  |  |  |  |  |  |
| Chow et al. 2021 |  |  |  |  |  |  |  |  |  |  |  |  |  |  |  |  |  |  |  |  |  |  |  |  |  |  |  |  |  |  |  |  |  |  |  |  |
| Chu et al. 2024 | June 2018 |  |  |  |  |  |  |  |  |  |  |  |  |  |  |  |  |  |  |  |  |  |  |  |  |  |  |  |  |  |  |  |  |  |  | ⭢June 2022 |
| Clerici et al. 2020 |  |  |  |  |  |  |  |  |  |  |  |  |  |  |  |  |  |  |  |  |  |  |  |  |  |  |  |  |  |  |  |  |  |  |  |  |
| Davies et al. 2021 |  |  |  |  |  |  |  |  |  |  |  |  |  |  |  |  |  |  |  |  |  |  |  |  |  |  |  |  |  |  |  |  |  |  |  |  |
| deDiegoRuiz et al. 2023 |  |  |  |  |  |  |  |  |  |  |  |  |  |  |  |  |  |  |  |  |  |  |  |  |  |  |  |  |  |  |  |  |  |  |  |  |
| Der et al. 2023 | Jan 2016 |  |  |  |  |  |  |  |  |  |  |  |  |  |  |  |  |  |  |  |  |  |  |  |  |  |  |  |  |  |  |  |  |  |  |  |
| Di Lorenzo et al. 2021a |  |  |  |  |  |  |  |  |  |  |  |  |  |  |  |  |  |  |  |  |  |  |  |  |  |  |  |  |  |  |  |  |  |  |  |  |
| Di Lorenzo et al. 2021b |  |  |  |  |  |  |  |  |  |  |  |  |  |  |  |  |  |  |  |  |  |  |  |  |  |  |  |  |  |  |  |  |  |  |  |  |
| Di Valerio et al. 2024 |  |  |  |  |  |  |  |  |  |  |  |  |  |  |  |  |  |  |  |  |  |  |  |  |  |  |  |  |  |  |  |  |  |  |  |  |
| Dindar et al. 2024 |  |  |  |  |  |  |  |  |  |  |  |  |  |  |  |  |  |  |  |  |  |  |  |  |  |  |  |  |  |  |  |  |  |  |  |  |
| Engels et al. 2022 |  |  |  |  |  |  |  |  |  |  |  |  |  |  |  |  |  |  |  |  |  |  |  |  |  |  |  |  |  |  |  |  |  |  |  |  |
| Ettman et al. 2024 | Nov 2017 |  |  |  |  |  |  |  |  |  |  |  |  |  |  |  |  |  |  |  |  |  |  |  |  |  |  |  |  |  |  |  |  |  |  | ⭢Oct 2022 |
| Fasshauer et al. 2021a |  |  |  |  |  |  |  |  |  |  |  |  |  |  |  |  |  |  |  |  |  |  |  |  |  |  |  |  |  |  |  |  |  |  |  |  |
| Fasshauer et al. 2021b |  |  |  |  |  |  |  |  |  |  |  |  |  |  |  |  |  |  |  |  |  |  |  |  |  |  |  |  |  |  |  |  |  |  |  |  |
| Fellinger et al. 2023 | Jan 2018 |  |  |  |  |  |  |  |  |  |  |  |  |  |  |  |  |  |  |  |  |  |  |  |  |  |  |  |  |  |  |  |  |  |  |  |
| Flament et al. 2021 |  |  |  |  |  |  |  |  |  |  |  |  |  |  |  |  |  |  |  |  |  |  |  |  |  |  |  |  |  |  |  |  |  |  |  |  |
| Flodin et al. 2023 | Jan 2015 |  |  |  |  |  |  |  |  |  |  |  |  |  |  |  |  |  |  |  |  |  |  |  |  |  |  |  |  |  |  |  |  |  |  |  |
| Fstkchian et al. 2023 |  |  |  |  |  |  |  |  |  |  |  |  |  |  |  |  |  |  |  |  |  |  |  |  |  |  |  |  |  |  |  |  |  |  |  |  |
| Fu et al. 2024 | Mar 2018 |  |  |  |  |  |  |  |  |  |  |  |  |  |  |  |  |  |  |  |  |  |  |  |  |  |  |  |  |  |  |  |  |  |  |  |
| Fuster-Casanovas et al. 2024 | Jan 2017 |  |  |  |  |  |  |  |  |  |  |  |  |  |  |  |  |  |  |  |  |  |  |  |  |  |  |  |  |  |  |  |  |  |  |  |
| Gajdics et al. 2023 | Mar 2017 |  |  |  |  |  |  |  |  |  |  |  |  |  |  |  |  |  |  |  |  |  |  |  |  |  |  |  |  |  |  |  |  |  |  | ⭢May 2022 |
| Giménez-Palomo et al. 2024 |  |  |  |  |  |  |  |  |  |  |  |  |  |  |  |  |  |  |  |  |  |  |  |  |  |  |  |  |  |  |  |  |  |  |  |  |
| Goldschmidt et al. 2023 |  |  |  |  |  |  |  |  |  |  |  |  |  |  |  |  |  |  |  |  |  |  |  |  |  |  |  |  |  |  |  |  |  |  |  |  |
| Golubovic et al. 2022 |  |  |  |  |  |  |  |  |  |  |  |  |  |  |  |  |  |  |  |  |  |  |  |  |  |  |  |  |  |  |  |  |  |  |  |  |
| Gómez-Ramiro et al. 2021 |  |  |  |  |  |  |  |  |  |  |  |  |  |  |  |  |  |  |  |  |  |  |  |  |  |  |  |  |  |  |  |  |  |  |  |  |
| Goncalves-Pinho et al. 2020 |  |  |  |  |  |  |  |  |  |  |  |  |  |  |  |  |  |  |  |  |  |  |  |  |  |  |  |  |  |  |  |  |  |  |  |  |
| Hakansson et al. 2021 |  |  |  |  |  |  |  |  |  |  |  |  |  |  |  |  |  |  |  |  |  |  |  |  |  |  |  |  |  |  |  |  |  |  |  |  |
| Hamlin et al. 2022 |  |  |  |  |  |  |  |  |  |  |  |  |  |  |  |  |  |  |  |  |  |  |  |  |  |  |  |  |  |  |  |  |  |  |  |  |
| Hansen et al. 2024 |  |  |  |  |  |  |  |  |  |  |  |  |  |  |  |  |  |  |  |  |  |  |  |  |  |  |  |  |  |  |  |  |  |  |  |  |
| Holland et al. 2021 |  |  |  |  |  |  |  |  |  |  |  |  |  |  |  |  |  |  |  |  |  |  |  |  |  |  |  |  |  |  |  |  |  |  |  |  |
| Irigoyen-Otiñano et al. 2024a |  |  |  |  |  |  |  |  |  |  |  |  |  |  |  |  |  |  |  |  |  |  |  |  |  |  |  |  |  |  |  |  |  |  |  |  |
| Irigoyen-Otiñano et al. 2024b |  |  |  |  |  |  |  |  |  |  |  |  |  |  |  |  |  |  |  |  |  |  |  |  |  |  |  |  |  |  |  |  |  |  |  |  |
| Jagadheesan et al. 2021 a |  |  |  |  |  |  |  |  |  |  |  |  |  |  |  |  |  |  |  |  |  |  |  |  |  |  |  |  |  |  |  |  |  |  |  |  |
| Jagadheesan et al. 2021 b |  |  |  |  |  |  |  |  |  |  |  |  |  |  |  |  |  |  |  |  |  |  |  |  |  |  |  |  |  |  |  |  |  |  |  |  |
| Jahlan et al. 2022 |  |  |  |  |  |  |  |  |  |  |  |  |  |  |  |  |  |  |  |  |  |  |  |  |  |  |  |  |  |  |  |  |  |  |  |  |
| Jones et al. 2023 | Sep 2018 |  |  |  |  |  |  |  |  |  |  |  |  |  |  |  |  |  |  |  |  |  |  |  |  |  |  |  |  |  |  |  |  |  |  |  |
| Jones et al. 2024 | Jan 2018 |  |  |  |  |  |  |  |  |  |  |  |  |  |  |  |  |  |  |  |  |  |  |  |  |  |  |  |  |  |  |  |  |  |  |  |
| Joo et al. 2022 |  |  |  |  |  |  |  |  |  |  |  |  |  |  |  |  |  |  |  |  |  |  |  |  |  |  |  |  |  |  |  |  |  |  |  |  |
| Kim et al. 2023a |  |  |  |  |  |  |  |  |  |  |  |  |  |  |  |  |  |  |  |  |  |  |  |  |  |  |  |  |  |  |  |  |  |  |  |  |
| Kim et al. 2023b |  |  |  |  |  |  |  |  |  |  |  |  |  |  |  |  |  |  |  |  |  |  |  |  |  |  |  |  |  |  |  |  |  |  |  |  |
| Lee et al. 2020 |  |  |  |  |  |  |  |  |  |  |  |  |  |  |  |  |  |  |  |  |  |  |  |  |  |  |  |  |  |  |  |  |  |  |  |  |
| Lee et al. 2022 |  |  |  |  |  |  |  |  |  |  |  |  |  |  |  |  |  |  |  |  |  |  |  |  |  |  |  |  |  |  |  |  |  |  |  |  |
| Lee et al. 2023 |  |  |  |  |  |  |  |  |  |  |  |  |  |  |  |  |  |  |  |  |  |  |  |  |  |  |  |  |  |  |  |  |  |  |  |  |
| Lee et al. 2024 |  |  |  |  |  |  |  |  |  |  |  |  |  |  |  |  |  |  |  |  |  |  |  |  |  |  |  |  |  |  |  |  |  |  |  |  |
| Leonhardt et al. 2024 |  |  |  |  |  |  |  |  |  |  |  |  |  |  |  |  |  |  |  |  |  |  |  |  |  |  |  |  |  |  |  |  |  |  |  |  |
| Li et al. 2023 |  |  |  |  |  |  |  |  |  |  |  |  |  |  |  |  |  |  |  |  |  |  |  |  |  |  |  |  |  |  |  |  |  |  |  |  |
| Lieber et al. 2024 |  |  |  |  |  |  |  |  |  |  |  |  |  |  |  |  |  |  |  |  |  |  |  |  |  |  |  |  |  |  |  |  |  |  |  |  |
| Lin et al. 2023 |  |  |  |  |  |  |  |  |  |  |  |  |  |  |  |  |  |  |  |  |  |  |  |  |  |  |  |  |  |  |  |  |  |  |  |  |
| Ludwig et al. 2022 |  |  |  |  |  |  |  |  |  |  |  |  |  |  |  |  |  |  |  |  |  |  |  |  |  |  |  |  |  |  |  |  |  |  |  |  |

| Luo et al. 2024 |  |  |  |  |  |  |  |  |  |  |  |  |  |  |  |  |  |  |  |  |  |  |  |  |  |  |  |  |  |  |  |  |  |  |  |  |
| --- | --- | --- | --- | --- | --- | --- | --- | --- | --- | --- | --- | --- | --- | --- | --- | --- | --- | --- | --- | --- | --- | --- | --- | --- | --- | --- | --- | --- | --- | --- | --- | --- | --- | --- | --- | --- |
| Mangiapane et al. 2022 |  |  |  |  |  |  |  |  |  |  |  |  |  |  |  |  |  |  |  |  |  |  |  |  |  |  |  |  |  |  |  |  |  |  |  |  |
| McAndrew et al. 2021 |  |  |  |  |  |  |  |  |  |  |  |  |  |  |  |  |  |  |  |  |  |  |  |  |  |  |  |  |  |  |  |  |  |  |  |  |
| McDowell et al. 2021 |  |  |  |  |  |  |  |  |  |  |  |  |  |  |  |  |  |  |  |  |  |  |  |  |  |  |  |  |  |  |  |  |  |  |  |  |
| McKee et al. 2021 |  |  |  |  |  |  |  |  |  |  |  |  |  |  |  |  |  |  |  |  |  |  |  |  |  |  |  |  |  |  |  |  |  |  |  |  |
| Mehrabadi et al. 2024 |  |  |  |  |  |  |  |  |  |  |  |  |  |  |  |  |  |  |  |  |  |  |  |  |  |  |  |  |  |  |  |  |  |  |  |  |
| Minian et al. 2021 | Jan 2017 |  |  |  |  |  |  |  |  |  |  |  |  |  |  |  |  |  |  |  |  |  |  |  |  |  |  |  |  |  |  |  |  |  |  |  |
| Molina et al. 2022 |  |  |  |  |  |  |  |  |  |  |  |  |  |  |  |  |  |  |  |  |  |  |  |  |  |  |  |  |  |  |  |  |  |  |  |  |
| Montalbani et al. 2021 |  |  |  |  |  |  |  |  |  |  |  |  |  |  |  |  |  |  |  |  |  |  |  |  |  |  |  |  |  |  |  |  |  |  |  |  |
| Moreno-Martos et al. 2024 | Jan 2018 |  |  |  |  |  |  |  |  |  |  |  |  |  |  |  |  |  |  |  |  |  |  |  |  |  |  |  |  |  |  |  |  |  |  |  |
| Mukadam et al. 2021 |  |  |  |  |  |  |  |  |  |  |  |  |  |  |  |  |  |  |  |  |  |  |  |  |  |  |  |  |  |  |  |  |  |  |  |  |
| Muştucu et al. 2023 |  |  |  |  |  |  |  |  |  |  |  |  |  |  |  |  |  |  |  |  |  |  |  |  |  |  |  |  |  |  |  |  |  |  |  |  |
| Nejati et al. 2021 |  |  |  |  |  |  |  |  |  |  |  |  |  |  |  |  |  |  |  |  |  |  |  |  |  |  |  |  |  |  |  |  |  |  |  |  |
| Palzes et al. 2022 |  |  |  |  |  |  |  |  |  |  |  |  |  |  |  |  |  |  |  |  |  |  |  |  |  |  |  |  |  |  |  |  |  |  |  |  |
| Panariello et al. 2021 |  |  |  |  |  |  |  |  |  |  |  |  |  |  |  |  |  |  |  |  |  |  |  |  |  |  |  |  |  |  |  |  |  |  |  |  |
| Patel et al. 2021 |  |  |  |  |  |  |  |  |  |  |  |  |  |  |  |  |  |  |  |  |  |  |  |  |  |  |  |  |  |  |  |  |  |  |  |  |
| Perozziello et al. 2023 |  |  |  |  |  |  |  |  |  |  |  |  |  |  |  |  |  |  |  |  |  |  |  |  |  |  |  |  |  |  |  |  |  |  |  |  |
| Pignon et al. 2020 |  |  |  |  |  |  |  |  |  |  |  |  |  |  |  |  |  |  |  |  |  |  |  |  |  |  |  |  |  |  |  |  |  |  |  |  |
| Pikkel Igal et al. 2021 |  |  |  |  |  |  |  |  |  |  |  |  |  |  |  |  |  |  |  |  |  |  |  |  |  |  |  |  |  |  |  |  |  |  |  |  |
| Qamruddin et al. 2022 |  |  |  |  |  |  |  |  |  |  |  |  |  |  |  |  |  |  |  |  |  |  |  |  |  |  |  |  |  |  |  |  |  |  |  |  |
| Rachamin et al. 2023 |  |  |  |  |  |  |  |  |  |  |  |  |  |  |  |  |  |  |  |  |  |  |  |  |  |  |  |  |  |  |  |  |  |  |  |  |
| Ramadan et al. 2022 | Jan 2018 |  |  |  |  |  |  |  |  |  |  |  |  |  |  |  |  |  |  |  |  |  |  |  |  |  |  |  |  |  |  |  |  |  |  |  |
| Raventos et al. 2022 | Mar 2018 |  |  |  |  |  |  |  |  |  |  |  |  |  |  |  |  |  |  |  |  |  |  |  |  |  |  |  |  |  |  |  |  |  |  |  |
| Rice et al. 2025 |  |  |  |  |  |  |  |  |  |  |  |  |  |  |  |  |  |  |  |  |  |  |  |  |  |  |  |  |  |  |  |  |  |  |  |  |
| Romer et al. 2021 |  |  |  |  |  |  |  |  |  |  |  |  |  |  |  |  |  |  |  |  |  |  |  |  |  |  |  |  |  |  |  |  |  |  |  |  |
| Ross et al. 2023 |  |  |  |  |  |  |  |  |  |  |  |  |  |  |  |  |  |  |  |  |  |  |  |  |  |  |  |  |  |  |  |  |  |  |  |  |
| Rugova et al. 2024 |  |  |  |  |  |  |  |  |  |  |  |  |  |  |  |  |  |  |  |  |  |  |  |  |  |  |  |  |  |  |  |  |  |  |  |  |
| Russolillo et al. 2024 |  |  |  |  |  |  |  |  |  |  |  |  |  |  |  |  |  |  |  |  |  |  |  |  |  |  |  |  |  |  |  |  |  |  |  |  |
| Salamah et al. 2024 |  |  |  |  |  |  |  |  |  |  |  |  |  |  |  |  |  |  |  |  |  |  |  |  |  |  |  |  |  |  |  |  |  |  |  |  |
| Sanchez-Guarnido et al. 2022 |  |  |  |  |  |  |  |  |  |  |  |  |  |  |  |  |  |  |  |  |  |  |  |  |  |  |  |  |  |  |  |  |  |  |  |  |
| Savić et al. 2022 |  |  |  |  |  |  |  |  |  |  |  |  |  |  |  |  |  |  |  |  |  |  |  |  |  |  |  |  |  |  |  |  |  |  |  |  |
| Seifert et al. 2021 |  |  |  |  |  |  |  |  |  |  |  |  |  |  |  |  |  |  |  |  |  |  |  |  |  |  |  |  |  |  |  |  |  |  |  |  |
| Seo et al. 2021 |  |  |  |  |  |  |  |  |  |  |  |  |  |  |  |  |  |  |  |  |  |  |  |  |  |  |  |  |  |  |  |  |  |  |  |  |
| Silva-Valencia et al. 2024 | Jan 2018 |  |  |  |  |  |  |  |  |  |  |  |  |  |  |  |  |  |  |  |  |  |  |  |  |  |  |  |  |  |  |  |  |  |  |  |
| Simkin et al. 2022 |  |  |  |  |  |  |  |  |  |  |  |  |  |  |  |  |  |  |  |  |  |  |  |  |  |  |  |  |  |  |  |  |  |  |  |  |
| Simpson et al. 2021 |  |  |  |  |  |  |  |  |  |  |  |  |  |  |  |  |  |  |  |  |  |  |  |  |  |  |  |  |  |  |  |  |  |  |  |  |
| Sobetzko et al. 2021 |  |  |  |  |  |  |  |  |  |  |  |  |  |  |  |  |  |  |  |  |  |  |  |  |  |  |  |  |  |  |  |  |  |  |  |  |
| Stein et al. 2020 |  |  |  |  |  |  |  |  |  |  |  |  |  |  |  |  |  |  |  |  |  |  |  |  |  |  |  |  |  |  |  |  |  |  |  |  |
| Sweet et al. 2022 |  |  |  |  |  |  |  |  |  |  |  |  |  |  |  |  |  |  |  |  |  |  |  |  |  |  |  |  |  |  |  |  |  |  |  |  |
| Villarreal-Zegarra et al. 2023 |  |  |  |  |  |  |  |  |  |  |  |  |  |  |  |  |  |  |  |  |  |  |  |  |  |  |  |  |  |  |  |  |  |  |  |  |
| Visser et al. 2025 |  |  |  |  |  |  |  |  |  |  |  |  |  |  |  |  |  |  |  |  |  |  |  |  |  |  |  |  |  |  |  |  |  |  |  | ⭢March 2022 |
| Vukićević et al. 2025 |  |  |  |  |  |  |  |  |  |  |  |  |  |  |  |  |  |  |  |  |  |  |  |  |  |  |  |  |  |  |  |  |  |  |  |  |
| Vukojevic et al. 2021 |  |  |  |  |  |  |  |  |  |  |  |  |  |  |  |  |  |  |  |  |  |  |  |  |  |  |  |  |  |  |  |  |  |  |  |  |
| Wang et al. 2024 |  |  |  |  |  |  |  |  |  |  |  |  |  |  |  |  |  |  |  |  |  |  |  |  |  |  |  |  |  |  |  |  |  |  |  |  |
| Warwicker et al. 2023 |  |  |  |  |  |  |  |  |  |  |  |  |  |  |  |  |  |  |  |  |  |  |  |  |  |  |  |  |  |  |  |  |  |  |  |  |
| Wettstein et al. 2022 |  |  |  |  |  |  |  |  |  |  |  |  |  |  |  |  |  |  |  |  |  |  |  |  |  |  |  |  |  |  |  |  |  |  |  |  |
| Wullschleger et al. 2023 |  |  |  |  |  |  |  |  |  |  |  |  |  |  |  |  |  |  |  |  |  |  |  |  |  |  |  |  |  |  |  |  |  |  |  |  |
| Yalcin et al. 2021 |  |  |  |  |  |  |  |  |  |  |  |  |  |  |  |  |  |  |  |  |  |  |  |  |  |  |  |  |  |  |  |  |  |  |  |  |
| Yang et al. 2022 |  |  |  |  |  |  |  |  |  |  |  |  |  |  |  |  |  |  |  |  |  |  |  |  |  |  |  |  |  |  |  |  |  |  |  |  |
| Ying et al. 2023 | Dec 2018 |  |  |  |  |  |  |  |  |  |  |  |  |  |  |  |  |  |  |  |  |  |  |  |  |  |  |  |  |  |  |  |  |  |  |  |
| Zaki et al. 2022 |  |  |  |  |  |  |  |  |  |  |  |  |  |  |  |  |  |  |  |  |  |  |  |  |  |  |  |  |  |  |  |  |  |  |  |  |
| Zhang et al. 2022 |  |  |  |  |  |  |  |  |  |  |  |  |  |  |  |  |  |  |  |  |  |  |  |  |  |  |  |  |  |  |  |  |  |  |  |  |
| Zhang et al. 2023 |  |  |  |  |  |  |  |  |  |  |  |  |  |  |  |  |  |  |  |  |  |  |  |  |  |  |  |  |  |  |  |  |  |  |  |  |
| Zielasek et al. 2021 |  |  |  |  |  |  |  |  |  |  |  |  |  |  |  |  |  |  |  |  |  |  |  |  |  |  |  |  |  |  |  |  |  |  |  |  |
|  | J | F | M | A | M | J | J | A | S | O | N | D | J | F | M | A | M | J | J | A | S | O | N | D | J | F | M | A | M | J | J | A | S | O | N | D |
|  | **2019** | | | | | | | | | | | | **2020** | | | | | | | | | | | | **2021** | | | | | | | | | | | |

**Table S6 -** Definitions of the settings 'inpatient', 'outpatient', 'emergency', 'telemedicine' and 'medication' of the included studies

| study | inpatient | outpatient | Emergency department | telemedicine | medication |
| --- | --- | --- | --- | --- | --- |
| Abe et al. 2025 | hospitalizations due to mental disorders. data from 242 Japanese hospitals. | Number of new outpatient cases due to mental disorders before and during COVID-19 by sex and age, utilizing a difference-in-differences (DID) analysis with data from 242 Japanese hospitals. |  |  |  |
| Adorjan et al. 2021 | Survey data were collected over the course of one month in 38 out of 388 psychiatric hospitals contacted in Germany. |  |  |  |  |
| Ahmedani et al. 2024 |  | psychotherapy visits were defined as any visit greater than or equal to 30 minutes with a specialty mental healthprovider and with any CPT procedure code of 90785–90862, indicating either initial psychotherapy evaluation or indi-vidual psychotherapy |  | virtual care was identified byusing CPT codes with a GQ modifier for asynchronous tele-health video visits or with a GT modifier for synchronous andinteractive telehealth video visits, or was otherwise desig-nated as a telehealth video or telephone visit via local healthsystem records |  |
| Akkaoui et al. 2025 |  |  | consultation in the psychiatric Emergency Department |  |  |
| Alves et al. 2021 | Post-discharge destination: admitted to hospital (The Psychiatric Department of Médio Tejo Hospital Centre (CHMT) |  | visits to the psychiatric ED (CHMT) |  |  |
| Ambrosetti et al. 2021 | discharge decision made by the ED psychiatrist (nonvoluntary/voluntary hospitalization and returning home) |  | admissions to the adult division of the psychiatric ED of the University Hospital of Geneva |  |  |
| Anderson et al. 2022 |  |  | electronic medical record data on ED visits for adults collected via the National Syndromic Surveillance Program (NSSP) |  |  |
| Andersson et al. 2022 |  | addiction-specific psychiatric treatment facility: number of patient contacts |  |  |  |
| Bakolis et al. 2021 | MH inpatient services: number of new admissions; number of discharges and daily inpatient caseload | Community services |  |  |  |
| Balestrieri et al. 2021 | Admissions to General Hospital Psychiatric Units (GHPU) |  | consultations in hospital emergency department) (HED) |  |  |
| Baum et al. 2024 | Total Number of inpatient admissions, average length of inpatient stay, number of days in standard care and number of days in intensive (psychiatric) care | outpatient care: Total number of incident diagnoses, number of patients with at least one therapeutic session |  |  |  |
| Beghi et al. 2022 |  |  | emergency room admission |  |  |
| Berardelli et al. 2021 | clinical records of 632 adult psychiatric inpatients consecutively admitted to the psychiatric unit of Sant'Andrea University Hospital |  |  |  |  |
| Bhagavathula et al. 2024 |  |  |  | Medicaid telehealth claims data from the Medicaid and Children’s Health Insurance Program (CHIP) population database. The database comprised monthly counts and rates (per 1,000 beneficiaries) of behavioral health services across states. |  |
| Boldrini et al. 2021 | admissions records from 12 general hospital psychiatric wards in different Italian regions |  |  |  |  |
| Bonello et al. 2021 | all admissions to the only national mental health facility in Malta, using the electronic admissions register |  |  |  |  |
| Bruckner et al. 2023 |  |  | all psychiatric ED visits to the LAC + USC Medical Center. |  |  |
| Cafaro et al. 2022 | every access to the ED of Luigi Sacco Hospital and FBF Hospital in Milan. evaluation of mean of access to the ED (EMS and police intervention) and diagnosis at discharge from the ED (discharge, **psychiatric hospitalization**, non-psychiatric hospitalization, transfer to other psychiatric facility, ED abandonment, refusal of hospitalization) |  | every **access to the ED** of Luigi Sacco Hospital and FBF Hospital in Milan. evaluation of **mean of access to the ED** (EMS and police intervention) and **diagnosis at discharge from the ED** (discharge, psychiatric hospitalization, non-psychiatric hospitalization, transfer to other psychiatric facility, ED abandonment, refusal of hospitalization) |  |  |
| Capuzzi et al. 2020 |  |  | admissions to two [psychiatric emergency](https://www.sciencedirect.com/topics/pharmacology-toxicology-and-pharmaceutical-science/psychiatric-emergency) rooms of Department of Mental Health and Addiction (DMHA) offer psychiatric emergency care |  |  |
| Caselli et al. 2023 |  | collection of data relating to patients referring to the university psychiatric outpatient clinics of Varese, Azzate, and Arcisate (ASST, Azienda Socio-Sanitaria TerritorialeSetteLaghi, Varese, Italy) |  |  |  |
| Carr et al. 2021 |  | primary care electronic health records obtained from the Clinical Practice Research Datalink (CPRD) Aurum and GOLD databases: GP referrals to mental health services |  |  | primary care electronic health records obtained from the Clinical Practice Research Datalink (CPRD) Aurum and GOLD databases: prescriptions for antidepressants and benzodiazepines |
| Chen et al. 2020a |  | Daily referral numbers to mental health service teamsy |  |  |  |
| Chow et al. 2021 |  | all care contacts of patients with healthcare professionals (face-to-face out-patient contacts and home visits.) |  | video consultations or telephone contacts |  |
| Chu et al. 2024 | health administrative databases from Institute for Clinical Evaluative Sciences: data on all hospital and community-based ambulatory care (including emergency department visits) | health administrative databases from Institute for Clinical Evaluative Sciences: data on all physician claims for services provided to patients with healthcare coverage, all adults receiving mental health services in the province | health administrative databases from Institute for Clinical Evaluative Sciences: data on all hospital and community-based ambulatory care (including emergency department visits) |  | health administrative databases from Institute for Clinical Evaluative Sciences: records of prescription claims |
| Clerici et al. 2020 | The Lombardy Region has been equipped with an automated register since 1997: this register collects a variety of information concerning the regional NHS. Information includes data on patients in contact with public DMHAs, for example, sociodemographic data, ICD-10 diagnoses, treatments received, **hospital admissions and discharges, treatment settings**, use of day-hospital, and stay in residential facilities. |  |  |  |  |
| Correia et al. 2024 | hospitalizations (persons with live, in-hospital births in Ontario) | outpatient visits (identified through physician-reported diagnostic codes) |  |  |  |
| Davies et al. 2021 | anonymous data was collected for all in-patient admissions to Kent and Medway NHS and Social Care Partnership Trust (KMPT) |  |  |  |  |
| deDiegoRuiz et al. 2023 | Data from all psychiatric hospitalization admissions to the acute hospitalization unit |  |  |  |  |
| Der et al. 2023 | A mental health or substance use-related encounter was defined as an ED visit or **hospital admission** in which either the primary reason for ED visit or the final primary diagnosis, designated by International Classification of Diseases |  | A mental health or substance use-related encounter was defined as an ED visit or hospital admission in which either the primary reason for ED visit or the final primary diagnosis, designated by International Classification of Diseases |  |  |
| Di Lorenzo et al. 2021a |  | data were collected from the informative database (InfoClin) of Mental Health Center: referral to Urgent Psychiatric consultations |  | data were collected from the informative database (InfoClin) of Mental Health Center: telephonic consultation |  |
| Di Lorenzo et al. 2021b | Outcomes of Urgent Psychiatric Consultation: Voluntary or involuntary psychiatric hospitalization |  | Short-Stay observation in Emergency Room |  |  |
| Di Valerio et al. 2024 |  |  |  |  | Anonymized identifier attributed to each assisted resident, the individual consumption of AD drugs was identified through record-linkage between the registry of individuals assisted by the Regional Health Service and the regional databases of dispensed drugs |
| Dindar et al. 2024 | Data of the patients attending a community mental health center were analyzed: **emergency hospital admissions and hospitalization rate** |  |  |  |  |
| Engels et al. 2022 | effects of the Covid-19 pandemic on psychiatric care - a secondary data analysis based on AOK insurance data: inpatient cases |  |  |  | effect of the COVID-19 pandemic on psychiatric care – a secondary data analysis based on AOK insurance data: Daily Defined Doses (DDD) of psychotropic drugs |
| Ettman et al. 2024 |  | outpatient appointments in the Johns Hopkins Medicine Department of Psychiatry and Behavioral Sciences. Data on these mental health appointments were obtained from the electronic health records system |  | Appointments within the psychiatry department were coded as telepsychiatry when they were scheduled to be conducted via video or telephone |  |
| Fasshauer et al. 2021a | data from 67 Helios hospitals was performed: emergency admissions. Administrative data were extracted from QlikView (QlikTech, Radnor, Pennsylvania, USA). |  |  |  |  |
| Fasshauer et al. 2021b | daily psychiatric emergency admissions of several psychiatric hospitals in Germany |  |  |  |  |
| Fasshauer et al. 2022 | data from 13 Helios hospitals was performed: Inpatient admissions for psychiatric diagnoses. Administrative data were extracted from QlikView (QlikTech, Radnor, Pennsylvania, USA). |  |  |  |  |
| Fellinger et al. 2023 | data from the national patient advocacy VertretungsNetz: involuntarily admitted to a psychiatric hospital in Austria |  |  |  |  |
| Flament et al. 2021 |  |  | psychiatric visits to the ED of a university hospital. data were obtained by searching our medical records |  |  |
| Flodin et al. 2023 |  | records of primary care visits: Norway: the data set comprised 130.01 million primary care encounters Latvia: the database contained primary healthcare utilization records  Sweden: primary care data (encounters) from the four counties (Stockholm County, Västra Götaland County, Skåne County, and Östergötland County)  Netherlands: Dutch primary care data (encounters) |  |  |  |
| Fuster-Casanovas et al. 2024 |  | population of Catalonia that visited Primary care centers of the Catalan Institute of Health: face-to-face. The database was obtained through the Information System for the Development of Research in Primary Care |  | population of Catalonia that visited Primary Care centers of the Catalan Institute of Health: eHealth consultations (eConsulta, telephone, or video consultations). The database was obtained through the Information System for the Development of Research in Primary Care |  |
| Fstkchian et al. 2023 |  |  |  |  | change in morphine milligram equivalents (MME) defined as the difference between daily MME prescribed to the patient before the respective timeframe and the daily MME of the last prescription ordered in that same timeframe. |
| Fu et al. 2024 |  |  |  |  | data were obtained from the local primary care electronic patient records: antidepressant prescribing was quantified using the average monthly number of prescriptions |
| Gajdics et al. 2023 | medical charts of 697 inpatients (inpatient admissions). study analysed anonymous clinical data of the patients. |  |  |  |  |
| Giménez-Palomo et al. 2024 |  |  | records from patients visited the ED of the Hospital Clínic of Barcelona. The records included all the patients attending this service between 2019 and 2021. |  |  |
| Gómez-Ramiro et al. 2021 | data from electronic medical records of all patients admitted to the emergency psychiatric room of the Hospital Clínic of Barcelona |  | data from electronic medical records of all patients admitted to the psychiatric emergency room of the Hospital Clínic of Barcelona |  |  |
| Goldschmidt et al. 2023 | clinical documentation records of all admissions from the psychiatric emergency department at St. Hedwig Hospital (SHK) in Berlin |  | clinical documentation records of all presentations at the psychiatric emergency department at St. Hedwig Hospital (SHK) in Berlin |  |  |
| Golubovic et al. 2022 | clinical records from 104 adult psychiatric inpatients admitted at Psychiatric Clinic, University Clinic Center Niš, Serbia |  |  |  |  |
| Goncalves-Pinho et al. 2020 |  |  | administrative database that collects information of adult emergency department visits at the *Centro Hospitalar Universitário São João* (CHUSJ) in Portugal |  |  |
| Hamlin et al. 2022 | register based data collection was conducted using an administrative database for psychiatric emergency department visits and admission rates at the psychiatric units at Sahlgrenska University Hospital, Gothenburg, Sweden |  | register based data collection was conducted using an administrative database for psychiatric emergency department visits and admission rates at the psychiatric units at Sahlgrenska University Hospital, Gothenburg, Sweden |  |  |
| Hansen et al. 2024 | hospital discharge data for psychiatric presentations, using the National Minimum Dataset provided by the New Zealand Ministry of Health. This resource contains information on all New Zealand public and private hospital discharges, including emergency, medical, surgical, and psychiatric departments |  |  |  |  |
| Hakansson et al. 2021 |  |  | number of patients seen in emergency psychiatric facilities, one general psychiatric emergency unit and one addiction psychiatry emergency unit. Data described the total number of unique patients seen in the facility during each month |  |  |
| Holland et al. 2021 |  |  | Weekly emergency department visit counts and rates (electronic health data from ED) |  |  |
| Irigoyen-Otiñano et al. 2024a | digital medical records: number of visits to the emergency department (ED) for psychiatric reasons: admission after ED contact |  | digital medical records: number of visits to the emergency department (ED) for psychiatric reasons |  |  |
| Irigoyen-Otiñano et al. 2024b | digital medical records: number of visits to the emergency department (ED) for psychiatric reasons: admission after ED contact |  | digital medical records: number of visits to the emergency department (ED) for psychiatric reasons |  |  |
| Jagadheesan et al. 2021 a | patients who needed inpatient hospital treatment. This study was based on inpatients units four adult mental health services of the North Western Mental Health (NWMH) network of Melbourne Health |  |  |  |  |
| Jagadheesan et al. 2021 b |  |  | Adult patients of the North West Area Mental Health Service catchment area who visited two EDs were included. The hospital databases were sources of information |  |  |
| Jahlan et al. 2022 |  | any adult patient who have been referred to the psychiatric outpatient department or had psychiatric consultation during inpatient admission. data were collected from Health Information System (HIS) electronic patients’ records |  |  |  |
| Jones et al. 2023 |  |  |  | receipt of OUD-related telehealth services: Medicare Part A or B service claim with OUD diagnosis and tele health service code, | receipt of MOUD (MOUD from OTPs, buprenorphine in office-based settings, extended-release (ER) naltrex one in office-based settings: having a Part B claim with HCPCS codes corresponding to receipt of MOUD |
| Jones et al. 2024 |  |  | Routinely collected patient data, including demographics, ED presentation characteristics, and ED outcomes, were analyzed according to the time-period of presentation |  |  |
| Joo et al. 2022 | Health Insurance Review and Assessment database: monthly number or value of patients in psychiatric hospitalization, admissions to psychiatric hospitals | Health Insurance Review and Assessment database: monthly number or value of patients in psychiatric outpatients |  |  |  |
| Kim et al. 2023b | All voluntary and involuntary inpatient psychiatric admissions were eligible for the study. Data were collected from the electronic health records of inpatient psychiatric patients |  |  |  |  |
| Kim et al. 2023a | patients’ mental health service utilization by hospital admissions and admissions via the ED. Psychiatric patients’ data were extracted from the National Health Insurance Claims Database | patients’ mental health service utilization by outpatient visits. Psychiatric patients’ data were extracted from the National Health Insurance Claims Database |  |  |  |
| Lee et al. 2020 | All psychiatric admissions in a major gazette inpatient psychiatric unit in Hong Kong, ere anonymously reviewed | monthly utilization of outpatient and community psychogeriatric services: outpatient attendance |  | monthly utilization of outpatient and community psychogeriatric services: Telephone support |  |
| Lee et al. 2022 |  |  | National Emergency Department Information System (NEDIS) database: clinical and administrative information of patients who visit any of the 402 nationwide EDs |  |  |
| Lee et al. 2023 |  | outpatient psychiatric services including medication management, individual psychotherapy, group psychotherapy, interventional psychiatry |  |  |  |
| Lee et al. 2024 |  | outpatient mental health visits: data from patients seen in Massachusetts General Hospital |  |  |  |
| Leonhardt et al. 2024 | Norwegian Patient Register (NPR) with census data administered by Statistics Norway: data on specialist healthcare contacts and admissions, including referral dates, length of stay, diagnosis according to ICD-10, admission type, treatment codes |  |  |  |  |
| Li et al. 2023 |  | All outpatient treatments for SMI were collected retrospectively each month through the electronic health record (EHR) system |  |  |  |
| Lieber et al. 2024 | publicly available data from three registers, (a) the National Patient Register, (b) the National Prescribed Drug Register, and (c) the National Death Register: use of mental health services in terms of episodes of specialist psychiatric care | publicly available data from three registers, (a) the National Patient Register, (b) the National Prescribed Drug Register, and (c) the National Death Register: use of mental health services in terms of episodes of specialist psychiatric care |  |  | publicly available data from three registers, (a) the National Patient Register, (b) the National Prescribed Drug Register, and (c) the National Death Register: use of antidepressants and sedatives/anxiolytics |
| Lin et al. 203 |  | electronic medical record for patients enrolled in an outpatient psychiatry program |  |  |  |
| Luo et al. 2024 |  |  |  |  | changes in psychotropic drug prescribing rates: electronic health records and claims data from nine databases in France, Germany, Italy, the UK, South Korea, and the USA |
| Ludwig et al. 2022 |  |  |  |  | Prescription data for medicines for outpatients covered by statutory health insurance (GKV), Drug Prescription Report 2022 |
| Mangiapane et al. 2022 |  | Change in the use of services by statutory health insurance-accredited physicians during the COVID crisis, tabular trend report: psychotherapy |  |  |  |
| McAndrew et al. 2021 |  |  | Beaumont Hospital Liaison Psychiatry Department uses an Electronic Patient Record (EPR) to record clinical, demographic, and attendance details for all psychiatry presentations to the ED |  |  |
| McDowell et al. 2021 |  |  | data from electronic health records (EHR) of patients seen in the ED at Massachusetts General Hospital |  |  |
| McKee et al. 2021 |  |  |  |  | national and provincial patient-level longitudinal prescribing data from Canadian retail pharmacies: e.g. number of LAI new starts (i.e., occurrence of an LAI prescription, with no prior LAI prescription in the past 30-days) |
| Mehrabadi et al. 2024 | medical records within the University of California Healthcare System: patients admitted to the hospitals at the University of California Health’s five academic health centers |  |  |  |  |
| Minian et al. 2021 |  | enrolment and follow-up data from the Smoking Treatment for Ontario Patients (STOP) Program. STOP is an Ontario smoking cessation program |  |  |  |
| Molina et al. 2022 |  |  | Coordinated Care Management System (CCMS) is an SFDPH data platform: ED visits among high users of health and social services |  |  |
| Montalbani et al. 2021 |  |  | psychiatric consultation in the emergency department of an Italian hospital |  |  |
| Moreno-Martos et al. 2024 | Norway: National Patient Register (NPR): hospitalizations Sweden: the Swedish NPR collects data for specialist outpatient visits and hospitalizations. |  |  |  | Norway: Norwegian Prescription Database includes information on all prescribed medication irrespective of reimbursement dispensed to individuals in ambulatory care Sweden: Swedish National Prescribed Drug Register containing detailed data on all prescribed drugs dispensed at pharmacies in Sweden |
| Mukadam et al. 2021 |  |  | service-level data about numbers of presentations to three acute mental health liaison services (consultation psychiatry, detailed electronic clinical case records |  |  |
| Muştucu et al. 2023 |  |  | hospital database records of patients who were admitted in the emergency department and were referred to the psychiatry department for consultation in the Bursa Uludağ University Faculty of Medicine Hospital were analyzed |  |  |
| Nejati et al. 2021 | Data was collected for all patients admitted to inpatient acute care psychiatric services at the QEII Health Sciences Centre in Halifax, Nova Scotia |  |  |  |  |
| Palzes et al. 2022 | Using electronic health record (EHR) and claims data, we conducted a retrospective cohort study of adults having a nonremission alcohol use disorder documented at an outpatient, inpatient, emergency department, or telehealth encounter, or through a claim | Using electronic health record (EHR) and claims data, we conducted a retrospective cohort study of adults having a nonremission alcohol use disorder documented at an outpatient, inpatient, emergency department, or telehealth encounter, or through a claim |  | Using electronic health record (EHR) and claims data, we conducted a retrospective cohort study of adults having a nonremission alcohol use disorder documented at an outpatient, inpatient, emergency department, or telehealth encounter, or through a claim |  |
| Panariello et al. 2021 | For each patient hospitalized in the index periods, clinical data were collected, determining the diagnosis and the level of emergency/urgency at the time of admission based on the mental health triage by the Victorian Emergency Department |  |  |  |  |
| Patel et al. 2021 |  | In-person contacts: appointments attended by a patient and clinician recorded as a ‘Face To Face’ or ‘Group Contact’ Event Type. |  | Remote contacts: defined as appointments attended by a patient and clinician recorded as a ‘Phone’ or ‘Video (virtual) appointment’ Event Type |  |
| Perozziello et al. 2023 | data collected from the Groupe Hospitalier Universitaire medical and administrative information system, which includes inpatients’ discharge summaries and outpatients’ visits for all patients presenting at one of the GHU medical facilities | data collected from the Groupe Hospitalier Universitaire medical and administrative information system, which includes inpatients’ discharge summaries and outpatients’ visits for all patients presenting at one of the GHU medical facilities |  |  |  |
| Pignon et al. 2020 | number and characteristics of hospital admissions in three psychiatric emergency services. The data were extracted anonymously from registers |  | number and characteristics of emergency psychiatric consultations in three psychiatric emergency services. The data were extracted anonymously from registers |  |  |
| Pikkel Igal et al. 2021 |  |  | number of psychiatric visits to the emergency department in Rambam Health Care Campus. Medical records were retrieved using the MD Clone program by our hospital's computer team |  |  |
| Qamruddin et al. 2022 | The data was collected anonymously from the electronic medical records of the hospital: patients admitted to inpatient units at Al Amal Psychiatric Hospital |  |  |  |  |
| Rachamin et al. 2023 | Inpatient admission data was retrieved from the MedStat, an official database which collects inpatient data of all Swiss hospitals. Psychiatric admissions: all psychiatric admissions, admissions for affective disorders, neurotic disorders, and psychotic disorders. | Psychotherapy consultations: all psychotherapy consultations, and specific groups, namely face-to-face vs. teleconsultations, first vs. further consultations. |  |  | Psychotropic medication claims: all psychotropic medications, and specific groups, namely antidepressants, anxiolytics, and antipsychotics. |
| Romer et al. 2021 | number of hospitalized patients at psychiatric, in- patient clinics, using data from hospitals and EMS from The Capital Region of Denmark and Region Zealand |  |  |  |  |
| Ramadan et al. 2022 |  |  | Data was extracted from the administrative electronic health records system BestCare at King Abdulaziz Medical City (KAMC): trends in proportions of several mental health disorder at Emergency department (ED) visits |  |  |
| Raventos et al. 2022 |  | primary care records: pseudo-anonymised electronic health records: incidence of anxiety and depressive disorders |  |  |  |
| Rice et al. 2025 |  | VA electronic medical record data from the Corporate Data Warehouse: mental health visits |  | VA electronic medical record data from the Corporate Data Warehouse: mental health visits: phone and video mental health | VA electronic medical record data from the Corporate Data Warehouse: medication for OUD and AUD |
| Ross et al. 2023 | data from our electronic medical records: psychosis admissions |  |  |  |  |
| Rugova et al. 2024 |  |  |  |  | Data on marketed quantities of antidepressants (N06A) and anxiolytics (N05B) in Kosova |
| Russolillo et al. 2024 | health administrative data for adults with a psychiatric admission to a single urban hospital in Vancouver, BC |  |  |  |  |
| Salamah et al. 2024 | records of 5135 newly registered patients at the Rashid Hospital Psychiatry outpatient clinic over a five-year period, using the hospital’s electronic medical record system (CITRIX software – SALAMA). |  |  |  |  |
| Sanchez-Guarnido et al. 2022 |  | outcome: Use of psychological interventions (yes/no): in-person (individual/group) |  | outcome: Use of psychological interventions (yes/no): over the phone, videoconference (individual/group) |  |
| Savić et al. 2022 | number of patients seen in the same catchment area; at the University Psychiatric Hospital **admissions** data | number of patients seen in the same catchment area; at the University Psychiatric Hospital: data on regular visits through usual outpatient services | number of patients seen in the same catchment area; at the University Psychiatric Hospital: **emergency visits** data |  |  |
| Seifert et al. 2021 | Electronic patient documentation was used to extract relevant information: **inpatient treatment** |  | Electronic patient documentation was used to extract relevant information: **emergency consultation** |  |  |
| Seo et al. 2021 |  | Data for daily outpatient visits to the Department of Psychiatry at Severance Hospital, were collected retrospectively through the electronic health record (EHR) system |  |  |  |
| Silva-Valencia et al. 2024 |  | monthly mental health visit rates (per 1000 visits), aggregated by service modality (in-person) |  | monthly mental health visit rates (per 1000 visits), aggregated by service modality (virtual) |  |
| Simkin et al. 2022 |  | hospital liaison services: primary outcome variable was the presence of psychotic symptoms around the index date in the electronic health record |  |  |  |
| Simpson et al. 2021 |  |  | Change in Psychiatric Emergency Service Volume (number of patient encounters) |  |  |
| Sobetzko et al. 2021 | Need for admission (after ED contact) |  | Psychiatric emergency contacts in the interdisciplinary emergency department |  |  |
| Stein et al. 2020 |  |  | emergency department visits for mental-health-related conditions at the San Paolo University Hospital in Milan |  |  |
| Sweet et al. 2022 |  |  |  | use and type of tele-mental health services (medical vs. nonmedical) administered for the three mental health diagnosis |  |
| Villarreal-Zegarra et al. 2023 |  | mental health centers (CMHCs) in different areas of Peru. CMHCs are specialized mental health care centers that provide an array of health services, such as psychiatry, psychology, family medicine, nursing, social work, physical therapy, speech therapy, and rehabilitation |  |  |  |
| Visser et al. 2025 |  |  |  |  | pandemic-related changes in dispensed prescription trends, lockdown-related dispensed prescription dynamics |
| Vukićević et al. 2025 | psychiatric admissions at the University Hospital of Split, Croatia |  |  |  |  |
| Vukojevic et al. 2021 | Using the admission records, we extracted data on emergency department admissions in our institution |  |  |  |  |
| Wang et al. 2024 |  |  | electronic medical record (EMR) data to analyze Emergency Department visits from patients with MH disorders, SUDs, and SDOH |  |  |
| Warwicker et al. 2023 | All psychiatric inpatient admissions to Mount Carmel Hospital in the years 2019–2021 were reviewed in this retrospective longitudinal analysis. |  |  |  |  |
| Wettstein et al. 2022 | South Africa's private health sector: hospital claim data: beneficiaries admitted to a hospital | South Africa's private health sector: outpatient claim data: consulting outpatient care |  |  |  |
| Williams et al. 2020 |  | anonymized electronic health records: primary care data collected from 47 general practices in Salford |  |  | medication: Selective serotonin reuptake inhibitors |
| Wullschleger et al. 2023 | data were automatically retrieved from the clinic information system: number of psychiatric hospitalizations |  |  |  |  |
| Yalcin et al. 2021 |  |  | total number of patients admitted to the psychiatric emergency department |  |  |
| Yang et al. 2022 | All inpatients admitted after the onset of the COVID-19 were identified using the hospital’s information system (large tertiary teaching hospital in Shanghai) |  |  |  |  |
| Ying et al. 2023 |  |  |  |  | For each drug, the Pharmaceutical Information Network provided distinct monthly dispensing counts for the 26-month observation period of this study |
| Zaki et al. 2022 | characteristics of patients admitted to the two psychiatric units during this period and rates of local MH presentations were extracted from the health service database |  |  |  | usage rates of oral diazepam and lorazepam, and intramuscular midazolam (data was collected from acute adult psychiatric units in a major psychiatric hospital |
| Zhang et al. 2021b |  | Using data from the VHA Corporate Data Warehouse, we identified all veterans in the specified time-period receiving clinical encounters for core mental health disorders |  |  | Using data from the VHA Corporate Data Warehouse, we identified all veterans in the specified time-period receiving overdoses and/or filled psychopharma-cological medication prescriptions |
| Zhang et al. 2021a |  |  |  |  | Three prescription measures were derived by summing (a) weekly prescription fills, (b) number of patients with a specific medication on-hand, and (c) patients with new prescription |
| Zhang et al. 2023 |  |  | patients with anxiety-and stress-related mental illnesses who visited the Shanghai Mental Health Center (SMHC) psychiatric emergency department |  |  |
| Zielasek et al. 2021 | routine data of all inpatient and day-patient admission cases in all departments of the nine psychiatric hospitals of the LVR (general psychiatry, child and adolescent psychiatry, psychosomatic medicine, geriatric psychiatry, and addiction psychiatry) |  |  |  |  |

**Figure S2 a-e**funnel plots to assess risk of bias for each setting, categories of representativeness: A, B. short-term observation period (cut off: 8 months)

**2a. inpatient**

**
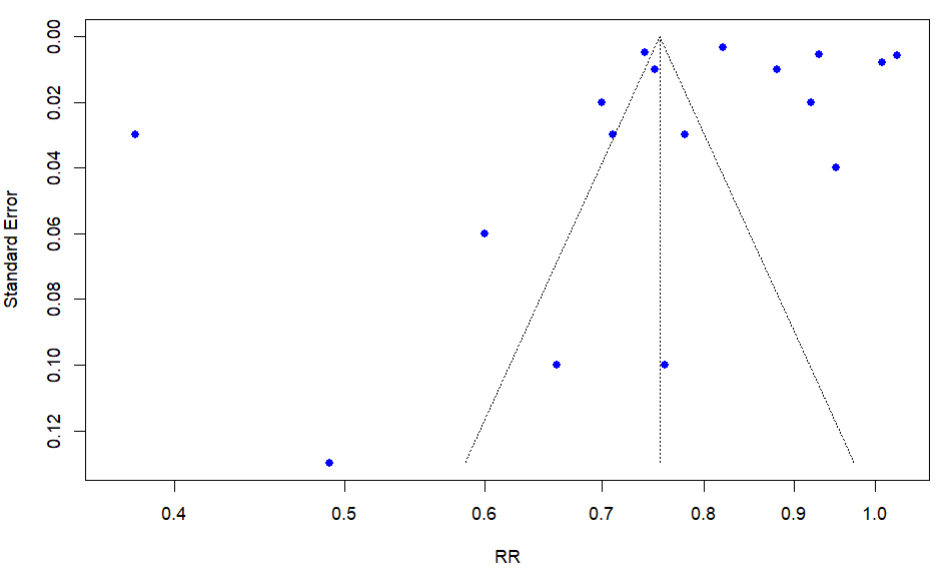
**

**2b. emergency department**

**
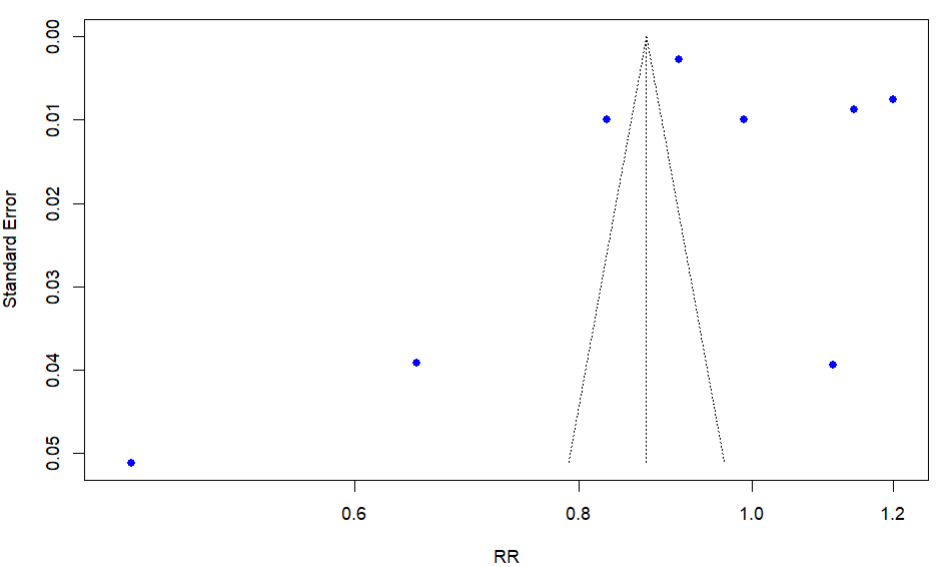
**

**2c. outpatient**

**
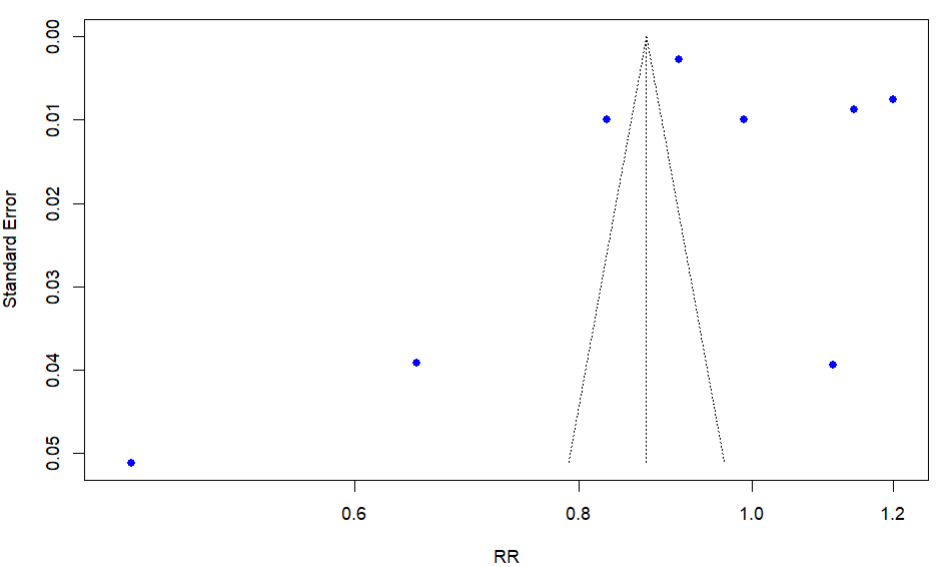
**

**2d. telemedicine**

**
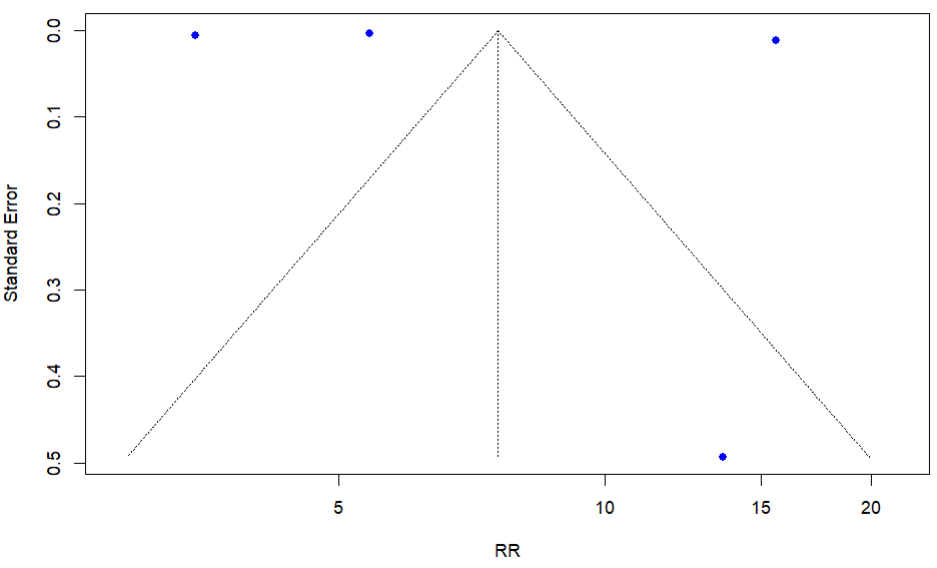
**

**2e. medication** (categories of representativeness: A, B)


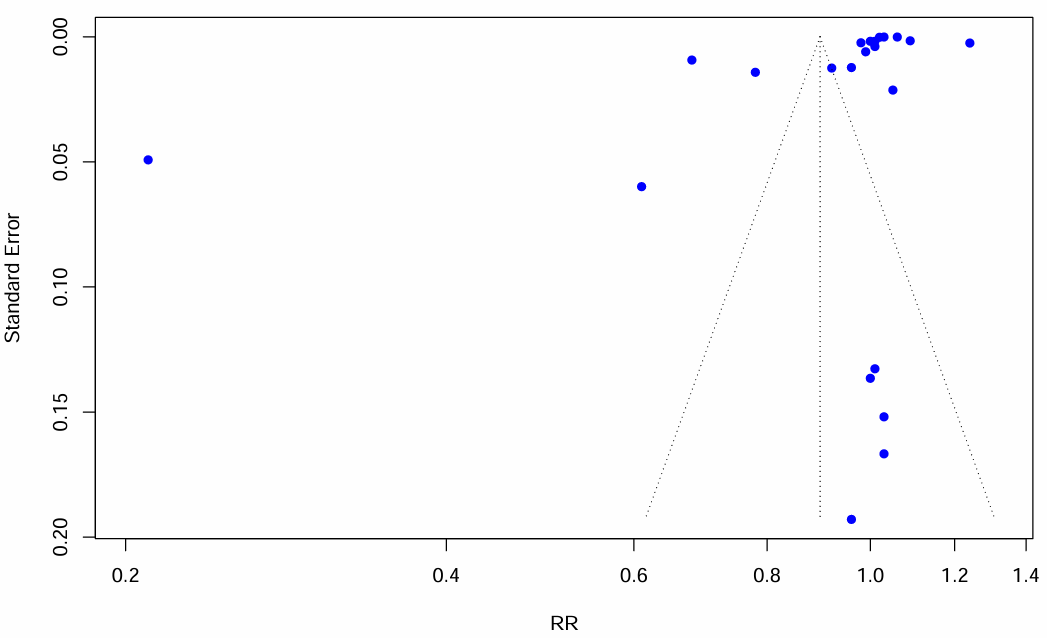


**Figure S3 a-b:** Forest Plots of Regional differences in psychiatric service utilization during the pandemic

**a. Inpatient Cases**


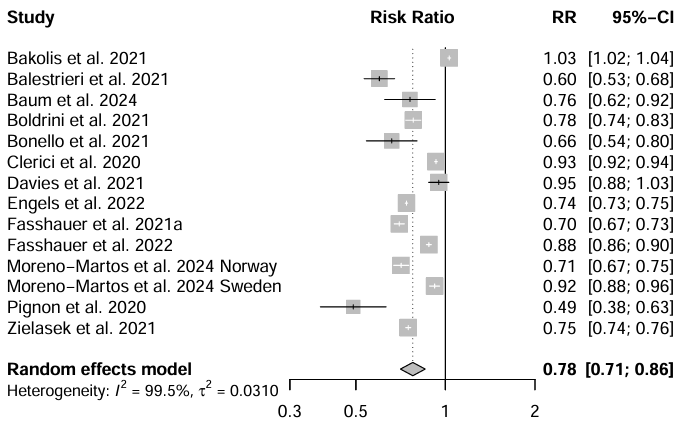
**Europe (EUR):** Inpatient Cases **Total** (A, B)


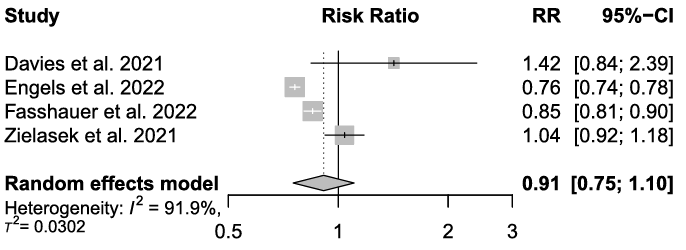
**Europe (EUR):** Inpatient Cases ICD-10 **F0 (A, B)**


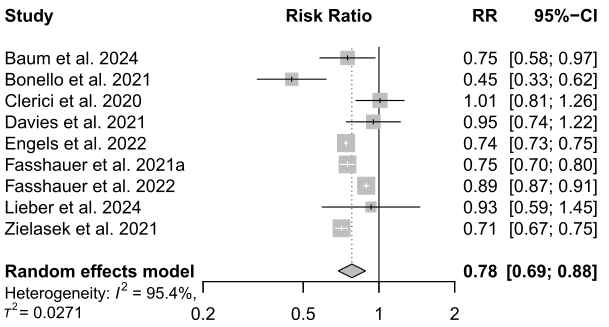
**Europe (EUR):** Inpatient Cases ICD-10 **F1** (A, B)


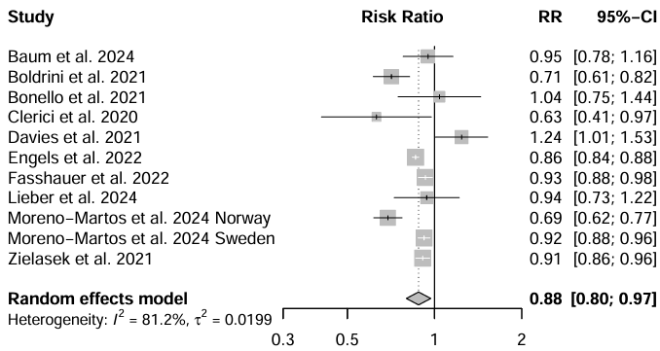
**Europe (EUR):** Inpatient Cases ICD-10 **F2** (A, B)

**Western Pacific Region (WPR):** Inpatient Cases ICD-10 **F2** (A, B)
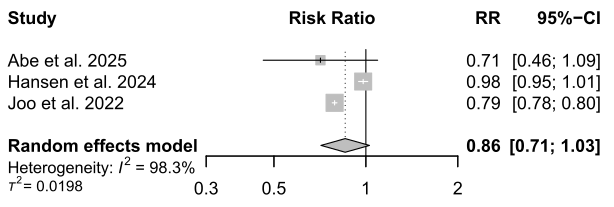


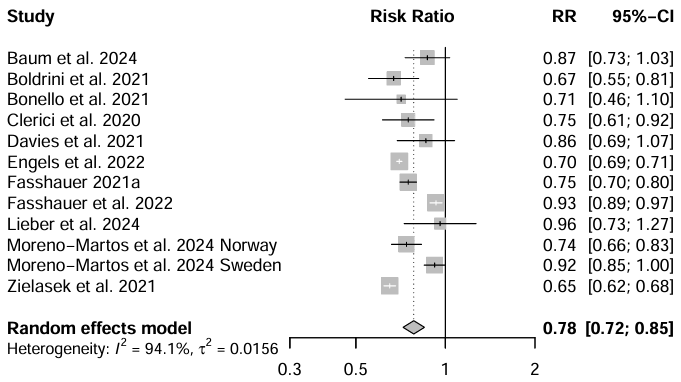
**Europe (EUR):** Inpatient Cases ICD-10 **F3** (A, B)

**Western Pacific Region (WPR):** Inpatient Cases ICD-10 **F3** (A, B)
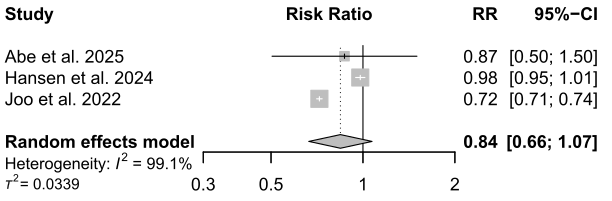


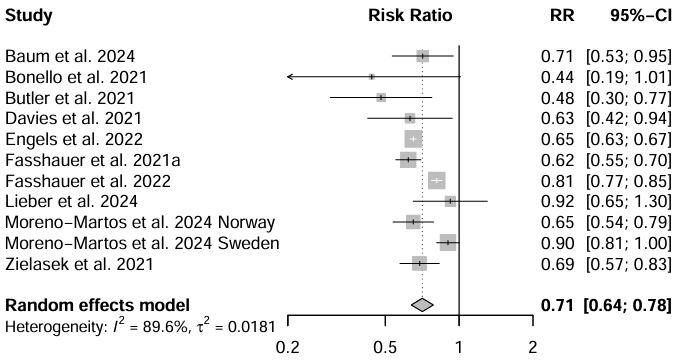
**Europe (EUR):** Inpatient Cases ICD-10 **F4** (A, B)

**Western Pacific Region (WPR):** Inpatient Cases ICD-10 **F4** (A, B)


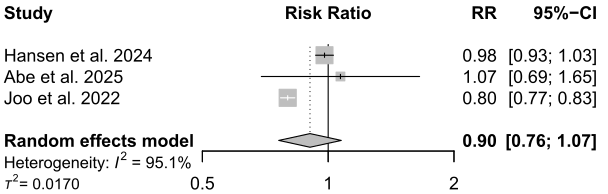


**Europe (EUR):** Inpatient Cases ICD-10 **F6** (A, B)


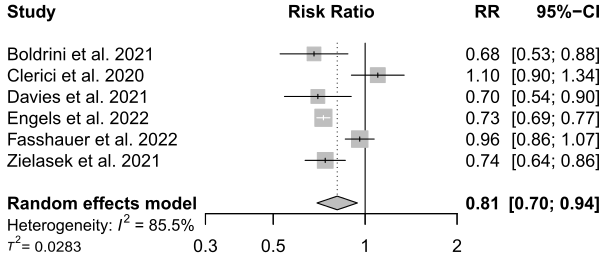


**b. Emergency Department Cases**

**Europe (EUR):** Emergency Department Cases **Total** (A, B)
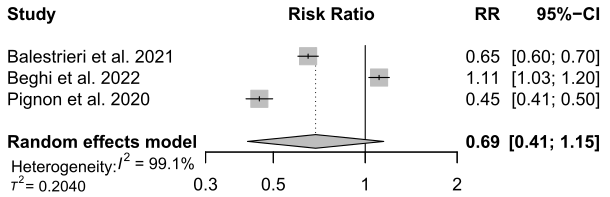


**American Region (AMR):** Emergency Department Cases **Total** (A, B)


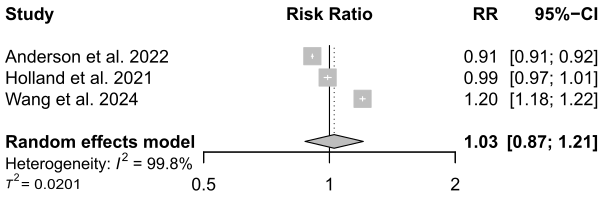


**Europe (EUR):** Emergency Department Cases **F1** (A, B)


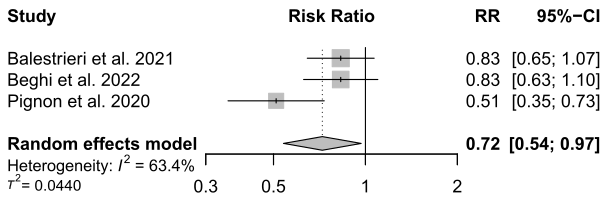


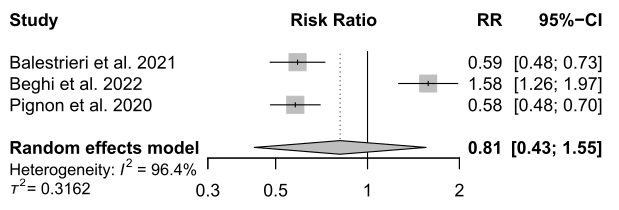
**Europe (EUR):** Emergency Department Cases **F2** (A, B)

**Europe (EUR):** Emergency Department Cases **F3** (A, B)


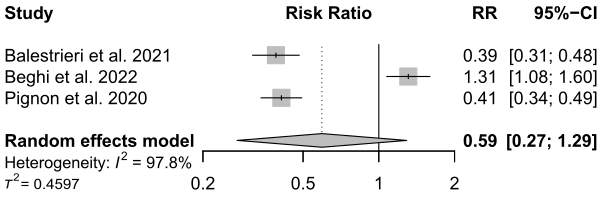


**Europe (EUR):** Emergency Department Cases **F4** (A, B)


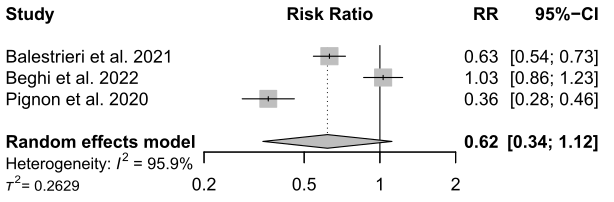


**American Region (AMR):** Emergency Department Cases **F4** (A, B)


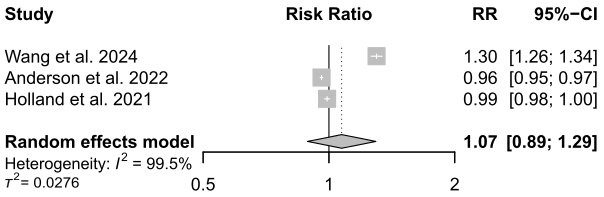


**Table S7 -** Categories for Representativeness

|  |  | **Category** |  |  |
| --- | --- | --- | --- | --- |
| **Study** | **Country** | **A** | **B** | **C** |
| Abe et al. 2025 | Japan |  | **242 acute-care hospitals in Japan, covering 11% of all hospital admissions in Japan** |  |
| Adorjan et al. 2021 | Germany |  | **38 clinics** |  |
| Ahmedani et al. 2024 | USA | **Data from the Mental Health Research Network - a consortium of several large healthcare systems in the USA with millions of insured patients** |  |  |
| Akkaoui et al. 2025 | France |  |  | **Single center data** |
| Alves et al. 2021 | Portugal |  |  | **a hospital centre** |
| Ambrosetti et al. 2021 | Switzerland |  |  | **University Hospital of Geneva** |
| Anderson et al. 2022 | USA | **ED visits from more than 3600 facilities from 49 states** |  |  |
| Andersson et al. 2022 | Sweden |  |  | **an addiction-specialized treatment facility** |
| Bakolis et al. 2021 | UK |  | **from 10 UK providers** |  |
| Balestrieri et al. 2021 | Italy |  | **9 Italian hospital EDs,** |  |
| Baum et al. 2024 | Germany | **Large, nationwide routine data from two statutory health insurance funds** |  |  |
| Beghi et al. 2022 | Italy | all seven public hospitals of AUSL Romagna |  |  |
| Berardelli et al. 2021 | Italy |  |  | **psychiatric unit of Sant ’Andrea Hospital** |
| Bhagavathula et al. 2024 | USA |  | **State-representative data from two states** |  |
| Boldrini et al. 2021 | Italy |  | **12 general psychiatric wards** |  |
| Bonello et al. 2021 | Malta | **national mental health facility in Malta** |  |  |
| Bruckner et al. 2023 | USA |  |  | **Single center data** |
| Butler et al. 2021 | UK |  |  | **a large acute teaching hospital** |
| Cafaro et al. 2022 | Italy |  |  | **two clinics in Mailand** |
| Capuzzi et al. 2020 | Italy |  |  | **in two psychiatric emergency services** |
| Carr et al. 2021 | UK | patient data from 1,697 general practices across the UK (England, Scotland, Wales, Northern Ireland) |  |  |
| Caselli et al. 2023 | Italy |  | **three university outpatient clinics** |  |
| Chen et al. 2020a | UK | **data from Cambridgeshire and Peterborough NHS Foundation Trust (CPFT), UK (catchment population ~0.86 million)** |  |  |
| Chow et al. 2021 | Netherlands |  |  | **a large dutch mental healthcare institute** |
| Chu et al. 2024 | Canada | **Population-wide routine data** |  |  |
| Clerici et al. 2020 | Italy |  | **seven General Hospital Psychiatric Wards** |  |
| Davies et al. 2021 | UK | **KMPT (Kent County, Medway Council Area): provide services to our local population of 1.8 million** |  |  |
| deDiegoRuiz et al. 2023 | Spain |  |  | **Single center data** |
| Der et al. 2023 | USA |  | **Regional data** |  |
| Di Lorenzo et al. 2021a | Italy |  |  | **outpatient Mental health Center of Modena** |
| Di Lorenzo et al. 2021b | Italy |  |  | **two Modena General Hospitals** |
| Di Valerio et al. 2024 | Italy | **complete regional medicines database of the Lazio region** |  |  |
| Dindar et al. 2024 | Turkey |  |  | **single Community Mental Health Centre (CMHC) in Istanbul** |
| Engels et al. 2022 | Germany | **AOK insurance data (2020 ca. 27 Mio. Versicherte, Marktanteil von ca. 37 %)** |  |  |
| Ettman et al. 2024 | USA |  | **one academic health system (not nationwide), multiple sites (outpatient), but no nationwide data coverage** |  |
| Fasshauer et al. 2021a | Germany |  | **from 67 hospitals** |  |
| Fasshauer et al. 2021b | Germany |  | **13 hospitals** |  |
| Fasshauer et al. 2022 | Germany |  | **several psychiatric hospitals** |  |
| Fellinger et al. 2023 | Austria | **nationwide data of Austria** |  |  |
| Flament et al. 2021 | Belgium |  |  | **a university hospital** |
| Flodin et al. 2023 | Norway, Latvia | **Norway: full national coverage of publicly funded primary care visits, Latvia: pertaining to the full Latvian population**  **Sweden: data from four major counties (Stockholm, Västra Götaland, Skåne, Östergötland)**  **Netherlands: Nivel Primary Care Database, covering primary care for 17.4 million inhabitants** |  |  |
| Fstkchian et al. 2023 | USA |  |  | **single academic medical centre in Southern California** |
| Fu et al. 2024 | United Kingdom |  |  | **Data from individual practices** |
| Fuster-Casanovas et al. 2024 | Spain |  | **80% of the PC teams in Catalonia** |  |
| Gajdics et al. 2023 | Hungary |  |  | **Single center data** |
| Giménez-Palomo et al. 2024 | Spain |  |  | **Single center data** |
| Goldschmidt et al. 2023 | Germany |  |  | **single clinic: the Psychiatric University Clinic of the Charité at St Hedwig Hospital** |
| Golubovic et al. 2022 | Serbia |  |  | **University Clinic Center** |
| Gómez-Ramiro et al. 2021 | Spain |  |  | **Hospital Clinic of Barcelona** |
| Goncalves-Pinho et al. 2020 | Portugal |  |  | **University Hospital Center** |
| Haggerty et al. 2022 | USA | **The PDMP database: data on substances that are dispensed by practitioners to West Virginia residents**  **Einschlusskriterien prüfen** |  |  |
| Hakansson et al. 2021 | Sweden |  |  | **two emergency psychiatric facilities** |
| Hamlin et al. 2022 | Sweden |  |  | **Single center data** |
| Hansen et al. 2024 | New Zealand | **National billing database (complete recording of all inpatient cases in New Zealand)** |  |  |
| Holland et al. 2021 | USA | approximately 70% of all US emergency departments (>3,500 EDs) across 48 states and Washington, D.C. |  |  |
| Irigoyen-Otiñano et al. 2024a | Spain |  |  | **single hospital (Santa María de Lleida University Hospital)** |
| Irigoyen-Otiñano et al. 2024b | Spain |  |  | **Single center data** |
| Jagadheesan et al. 2021 a | Australia |  |  | **large mental health network in Melbourne** |
| Jagadheesan et al. 2021 b | Australia |  |  | **mental health service of the Royal Melbourne Hospital** |
| Jahlan et al. 2022 | Saudi-Arabia |  |  | **Single center data** |
| Jones et al. 2023 | USA | **Medicare data at national level, highly representative of older and chronically ill adults** |  |  |
| Jones et al. 2024 | Australia | **routine data of all public EDs in Queensland (state with ~5.2 million inhabitants)** |  |  |
| Joo et al. 2022 | South Korea | **complete survey of the national healthcare database** |  |  |
| Kim et al. 2023a | South Korea | **South Korea's National Health Insurance Claims Database (NHICD), which contains all billing-relevant benefits for the entire country** |  |  |
| Kim et al. 2023b | USA |  |  | **Single centre, psychiatric hospital** |
| Lee et al. 2020 | China |  |  | **psychiatric unit in Hong Kong** |
| Lee et al. 2022 | Korea | complete population-based routine data, covering all 402 nationwide EDs |  |  |
| Lee et al. 2023 | USA |  |  | **two specific clinics at one university** |
| Lee et al. 2024 | USA |  | **several large hospitals and clinics** |  |
| Leonhardt et al. 2024 | Norway | **complete survey at national level via the Norwegian Patient Register** |  |  |
| Li et al. 2023 | China | **Complete survey of all psychiatric outpatient EHR contacts in the Ningbo region** |  |  |
| Lieber et al. 2024 | Sweden | **National population data, complete register coverage** |  |  |
| Lin et al. 2023 | USA |  |  | **Single center data** |
| Ludwig et al. 2022 | Germany | **Drug Prescription Report (prescription data from statutory health insurance)** |  |  |
| Luo et al. 2024 | France, Italy, UK, USA | **the most representative database of the respective country** |  |  |
| Luo et al. 2024 | Germany |  | **Several facilities with good coverage, but not fully national** |  |
| Luo et al. 2024 | South Korea |  |  | **Hospital-based EMRs from two university hospitals** |
| Mangiapane et al. 2022 | Germany | **German trend report based on billing data from 16 Regional Associations of Statutory Health Insurance Physicians (KVs)** |  |  |
| McAndrew et al. 2021 | Ireland |  |  | **large academic teaching hospital** |
| McDowell et al. 2021 | USA |  |  | **in a large tertiary care hospital** |
| McKee et al. 2021 | Canada | **LAIA prescribing practices from Canadian retail pharmacies(72% of national pharmacy prescriptions in Canada)** |  |  |
| Mehrabadi et al. 2024 | USA |  | **five academic health centers** |  |
| Minian et al. 2021 | Canada |  |  | **publicly funded smoking cessation program in Ontario, Canada** |
| Molina et al. 2022 | USA | complete population-based routine data from the San Francisco Department of Public Health |  |  |
| Montalbani et al. 2021 | Italy |  |  | **ED of an Italian hospital** |
| Moreno-Martos et al. 2024 | Norway | **national registries in Norway and Sweden** |  |  |
| Mukadam et al. 2021 | UK |  | **three mental health liaison teams and two mental healthcare centers** |  |
| Muştucu et al. 2023 | Turkey |  |  | **single university hospital** |
| Nejati et al. 2021 | Canada |  |  | **an urban acute care psychiatric centre** |
| Palzes et al. 2022 | USA |  | **Complete routine data from an integrated care system** |  |
| Panariello et al. 2021 | Italy |  |  | **Maggiore” Hospital in Bologna** |
| Patel et al. 2021 | UK | complete population-based routine data from the South London and Maudsley (SLaM) NHS Foundation Trust |  |  |
| Perozziello et al. 2023 | France |  |  | **single university hospital** |
| Pignon et al. 2020 | France |  | **three emergency services** |  |
| Pikkel Igal et al. 2021 | Israel |  |  | **ED in Rambam Health Care Campus** |
| Qamruddin et al. 2022 | United Arab Emirates |  |  | **single centre study** |
| Rachamin et al. 2023 | Switzerland | **nationwide coverage by MedStat** |  |  |
| Ramadan et al. 2022 | Saudi-Arabia |  | **multi center data** |  |
| Raventos et al. 2022 | Spain | **80% of the population in Catalonia** |  |  |
| Rice et al. 2025 | USA | **Department of Veterans Affairs medical records** |  |  |
| Romer et al. 2021 | Denmark | **health records covering 46% of the Danish population (*n* = 2,693,924)** |  |  |
| Ross et al. 2023 | USA |  |  | **Single center data** |
| Rugova et al. 2024 | Kosovo |  |  | **Kosova Medicines Agency** |
| Russolillo et al. 2024 | Canada |  |  | **Single center data** |
| Salamah et al. 2024 | Dubai |  |  | **Single center data** |
| Sanchez-Guarnido et al. 2022 | Spain |  | **in 15 outpatient mental health services** |  |
| Savić et al. 2022 | Kroata |  |  | **Single center data** |
| Seifert et al. 2021 | Germany |  |  | **an academic teaching hospital** |
| Seo et al. 2021 | South Korea |  |  | **in a tertiary hospital** |
| Silva-Valencia et al. 2024 | Argentina, Canada, Norway, Peru, Singapore, Sweden | **Argentina: National health administrative data**  **Canada: National billing system for public healthcare, Norway: Complete provincial data, system-dominant, Peru: National health registry data,**  **Singapore: National health registry data, Sweden: National Patient Register** |  |  |
| Silva-Valencia et al. 2024 | China, USA |  | **China: One province only, not nationwide**  **USA: Not national; privately insured population only** |  |
| Simkin et al. 2022 | United Kingdom |  | **large regional specialized mental health service** |  |
| Simpson et al. 2021 | USA |  | **three psychiatric emergency services** |  |
| Sobetzko et al. 2021 | Germany |  |  | **single center data** |
| Stein et al. 2020 | Italy |  |  | **at a University Hospital** |
| Sweet et al. 2022 | USA |  | **use of tele-mental health services using a large health system database (rural)** |  |
| Villarreal-Zegarra et al. 2023 | Peru | **nationwide data from all functional community mental health centers in Peru** |  |  |
| Visser et al. 2025 | Netherlands | **National register data** |  |  |
| Vukićević et al. 2025 | Croatia |  |  | **Single center data** |
| Vukojevic et al. 2021 | Croatia |  |  | **single center study** |
| Wang et al. 2024 | USA |  | **No national registry study, but large sample across multiple levels of care** |  |
| Warwicker et al. 2023 | Malta |  |  | **Single center data** |
| Wettstein et al. 2022 | South Africa |  | **largest open medical schemes that insured over 700 000 individuals** |  |
| Williams et al. 2020 | UK |  | **47 general practices** |  |
| Wullschleger et al. 2023 | Switzerland |  |  | **A single university psychiatric department** |
| Yalcin et al. 2021 | Turkey |  |  | **mental health epicenter** |
| Yang et al. 2022 | China |  |  | **in a tertiary teaching hospital** |
| Ying et al. 2023 | Canada | **using a centralized registry that captures all pharmacy dispensings in the province, thus providing near-complete population-level data** |  |  |
| Zaki et al. 2022 | Australia |  |  | **two acute psychiatric inpatient units** |
| Zhang et al. 2021 | USA |  |  | **a Veterans Health Administration** |
| Zhang et al. 2022 | USA | **largest integrated mental health treatment provider in the United States** |  |  |
| Zhang et al. 2023 | China |  |  | **Single center data, Shanghai Metal Health Center** |
| Zielasek et al. 2021 | Germany |  | **in 9 psychiatric hospitals** |  |
|  |  |  |  |  |
|  |  |  |  |  |

**Figure S8 a-d -** Supplementary Forest Plots for short-term (cut-off: 8 months) and long-Term. Category of representativeness: C

**a. Inpatient Cases**


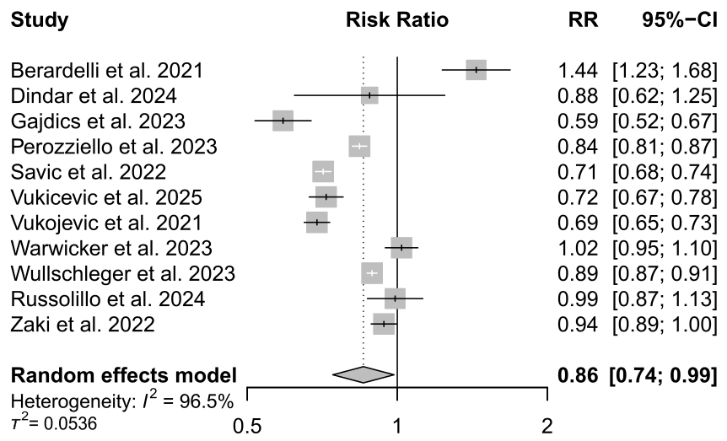
Inpatient Cases **Total**, short-term Inpatient Cases **Total**, long-term


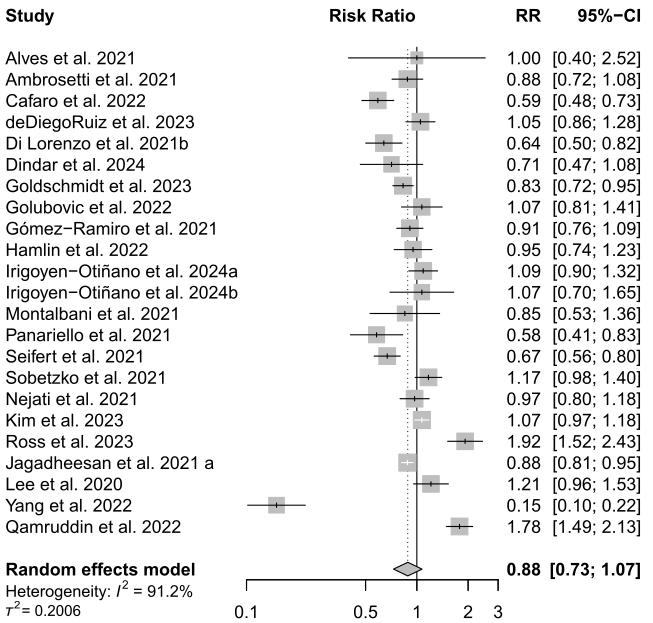


**b. Emergency Department Cases**


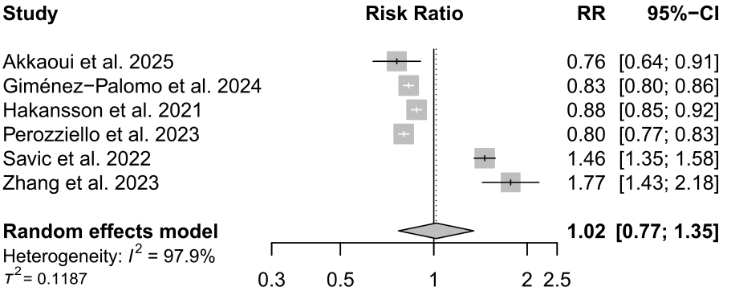

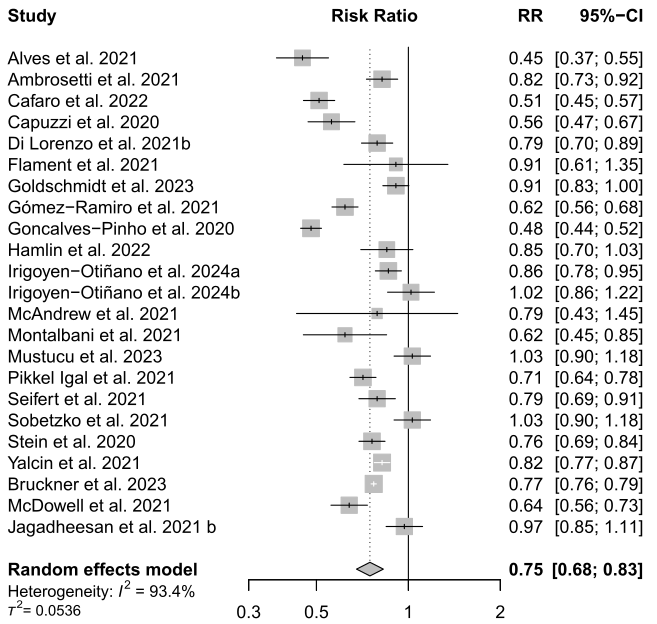
Emergency Department Cases **Total**, short-term Emergency Department Cases **Total**, long-term

**c. Outpatient Cases**


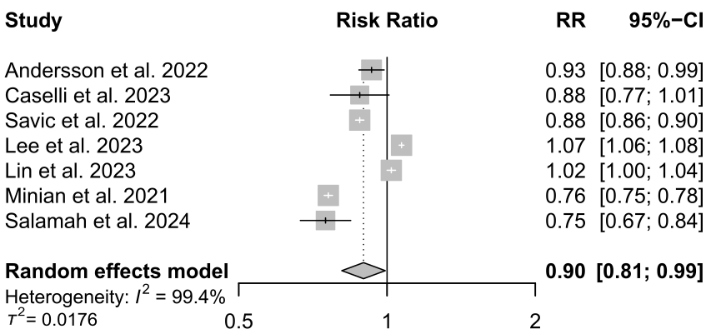
 Outpatient Cases **Total**, short-term Outpatient Cases **Total**, long-term


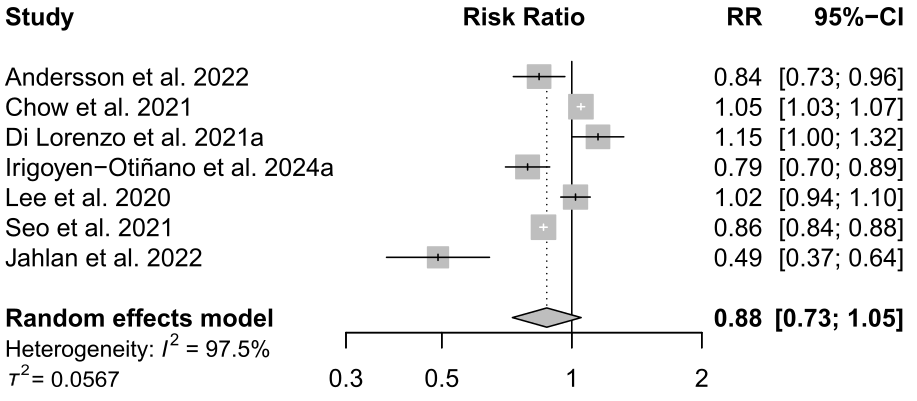


**d. Medication Prescriptions**

Medication Prescriptions **Total**, long-term


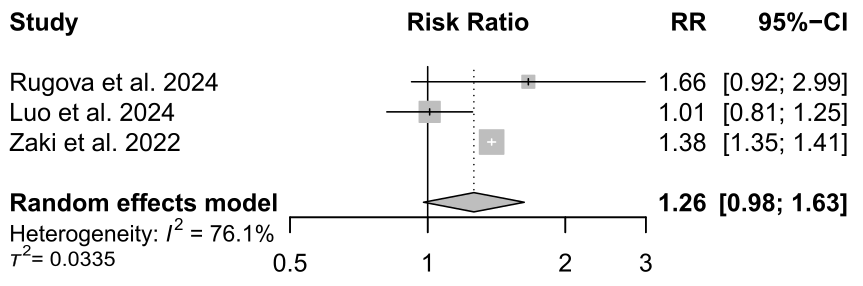


**Figure S9 -** Search strategies for the three databases

**PubMed**

1."mental illness"[Title/Abstract]

2."mental health"[MeSH Terms]

3."psychiatric emergency admission*"[Title/Abstract]

4."psychiatric hospitalization*"[Title/Abstract]

5."inpatient admission*"[Title/Abstract]

6."increased admission*"[Title/Abstract]

7."inpatient psychiatry"[Title/Abstract]

8."mental healthcare"[Title/Abstract]

9."self harm"[Title/Abstract]

10."substance use"[Title/Abstract]

11."outpatient psychiatr*"[Title/Abstract]

12."psychiatric presentation*"[Title/Abstract])

13."emergency presentation*"[Title/Abstract]

14."suicidal behavior"[Title/Abstract])

15. OR/#1-#14

16."psychiatric emergency department"[Title/Abstract]

17."psychiatric admission*"[Title/Abstract]

18."multicenter study"[Title/Abstract]

19."mental health cohort"[Title/Abstract]

20."cross sectional study"[Title/Abstract]

21."interrupted time series"[Title/Abstract]

22."retrospective study"[Title/Abstract]

23."retrospective cohort"[Title/Abstract]

24."electronic health record study"[Title/Abstract]

25."service database"[Title/Abstract]

26."mental healthcare data"[Title/Abstract]

27."routine data"[Title/Abstract]

28."european psychiatric association"[Title/Abstract]

29."psychiatric emergency service"[Title/Abstract]

30."primary care electronic health records"[Title/Abstract]

31."psychosis clinic"[Title/Abstract])

32. OR/#16-#31

33."coronavirus"[Title/Abstract]

34."corona"[Title/Abstract]

35."covid 19"[Title/Abstract]

36."2019 ncov"[Title/Abstract]

37."sars cov 2"[Title/Abstract]

38."covid 19"[MeSH Terms]

39."sars cov 2"[MeSH Terms]

40. OR/#33-#39

42. #15 AND #32 AND #40

**Ovid PsychInfo**

S1 TI (coronavirus) OR AB (coronavirus)

S2 TI (corona) OR AB (corona)

S3 TI (covid-19) OR AB (covid-19)

S4 TI (sars-cov-2) OR AB (sars-cov-2)

S5 MA (covid-19)

S6 MA (sars-cov-2)

S7 S1 OR S2 OR S3 OR S4 OR S5 OR S6

S8 TI (prescri* N3 (mental N1 health)) OR AB (prescri* N3 (mental N1 health))

S9 TI (utilization N2 (mental N1 health)) OR AB (utilization N2 (mental N1 health))

S10 TI (increas* N3 (mental N1 health)) OR AB (increas* N3 (mental N1 health))

S11 TI (decreas* N3 (mental N1 health)) OR AB (decreas* N3 (mental N1 health))

S12 TI (emergenc* N3 (mental N1 health)) OR AB (emergenc* N3 (mental N1 health))

S13 TI (emergenc* N3 (presentation)) OR AB (emergenc* N3 (presentation))

S14 TI (inpatient* N3 (mental N1 health)) OR AB (inpatient* N3 (mental N1 health))

S15 TI (outpatient* N3 (mental N1 health)) OR AB (outpatient* N3 (mental N1 health))

S16 TI (hospitalization N3 (mental N1 health)) OR AB (hospitalization N3 (mental N1 health))

S17 TI psychiatr* OR AB psychiatr*

S18 TI ((substance N1 use) or (substance N1 abuse)) OR AB ((substance N1 use) or (substance N1 abuse))

S19 MA (mental health)

S20 TI (mental N1 health) OR AB (mental N1 health)

S21 S8 OR S9 OR S10 OR S11 OR S12 OR S13 OR S14 OR S15 OR S16 OR S17 OR S18 OR S19 OR S20

S22 TI (longitudinal N2 stud*) OR AB (longitudinal N2 stud*)

S23TI (prospective N1 stud*) OR AB (prospective N1 stud*)

S24 TI (retrospective N1 stud*) OR AB (retrospective N1 stud*)

S25 TI (quantitative N1 stud*) OR AB (quantitative N1 stud*)

S26 TI (data N1 resource) OR AB (data N1 resource)

S27 TI (electronic health records) OR AB (electronic health records)

S28 TI (psychiatric admission*) OR AB (psychiatric admission*)

S29 S22 OR S23 OR S24 OR S25 OR S26 OR S27 OR S28

S30 S7 AND S21 AND S29

**Embase**

1. coronavirus.tw,kf.
2. corona.tw,kf.
3. covid 19.tw,kf.
4. sars cov 2.tw,kf.
5. exp Covid-19/
6. exp sars-CoV-2/
7. (prospective adj2 stud*).tw,kf.
8. (retrospective adj2 stud*).tw,kf.
9. (quantitative adj2 stud*).tw,kf.
10. longitudinal.tw,kf.
11. data resource.tw,kf.
12. Electronic Health Records.tw,kf.
13. (Utilisation adj2 mental health).tw,kf.
14. (Utilization adj2 mental health).tw,kf.
15. increas* adj3 mental health).tw,kf.
16. (decreas* adj3 mental health).tw,kf
17. (emergenc* adj3 mental health).tw,kf.
18. (inpatient* adj2 mental health).tw,kf.
19. (outpatient* adj2 mental health).tw,kf.
20. hospitalization adj3 mental health).tw,kf.
21. exp Mental Health/
22. (Emergenc* adj1 presentation).tw,kf.
23. (prescri* adj3 mental health).tw,kf.
24. (psychiatr* adj3 mental health).tw,kf.
25. (hospitalisation adj3 mental health).tw,kf.
26. 1 or 2 or 3 or 4 or 5 or 6
27. (substance use* adj3 mental health).tw,kf.
28. (inpatient adj2 psychiatr*).tw,kf.
29. (retrospective adj2 cohort).tw,kf.
30. time series study.tw,kf.
31. 7 or 8 or 9 or 10 or 11 or 12 or 29 or 30
32. 13 or 14 or 15 or 16 or 17 or 18 or 19 or 20 or 21 or 22 or 23 or 24 or 25 or 27 or 28 26 and 31 and 32
