# Supplementary material for: Utilization of mental health services during the first year of the COVID-19 pandemic – a systematic review and meta-analysis
Source: Eur Psychiatry. 2026 Jan 13;69(1):e10. doi: 10.1192/j.eurpsy.2025.10119 (PMC12816939; doi:10.1192/j.eurpsy.2025.10119)
Supplement: Glock et al. supplementary material 2 — Glock et al. supplementary material [file S0924933825101193sup002.docx]

|  | **Table 2 (cont.) – Page 1** | **study** | **outcome** | **RR** | **SE(lnRR)** | **95% CI total** | **WHO regions** | **country** | **category**  **representativity** | **mod. NOS** | **Covid-19- period  start** | **Covid-19 -period  end** | **CCHI-Index** | **SD CCHI-Index** | **Min. – Max.**  **(CCHI-Index)** |
| --- | --- | --- | --- | --- | --- | --- | --- | --- | --- | --- | --- | --- | --- | --- | --- |
| **inpatient short term**  **cut-off: 8 months** |  |  |  |  |  |  |  |  |  |  |  |  |  |  |  |
|  |  | Engels et al. 2022 | all psych. inpatient admissions | 0.74 | 0.00 | [0.73 – 0.74] | Europe | Germany | A | 6 | 01.03.2020 | 31.05.2020 | 56.52 | 13.06 | [27.98 -70.83] |
|  |  | Bonello et al. 2021 | all psych. inpatient admissions | 0.66 | 0.10 | [0.54 – 0.80] | Europe | Malta | A | 6 | 07.03.2020 | 04.06.2020 | 70.97 | 14.74 | [16.67 – 80.95] |
|  |  | Davies et al. 2021 | all psych. inpatient admissions | 0.95 | 0.04 | [0.88 – 1.02] | Europe | United Kingdom | A | 6 | 01.03.2020 | 31.08.2020 | 57.36 | 14.80 | [9.25 – 67.56] |
|  |  | Baum et al. 2024 | inpatient mental health care system | 0.76 | 0.10 | [0.63 – 0.91] | Europe | Germany | A | 6 | 01.03.2020 | 31.05.2020 | 56.52 | 13.06 | [27.98 -70.83] |
|  |  | Joo et al. 2022 | admissions to psychiatric hospitals | 0.82 | 0.00 | [0.80 – 0.82] | Western Pacific | South Korea | A | 6 | 01.02.2020 | 31.08.2020 | 53.05 | 10.94 | [14.88 – 73.81] |
|  |  | Moreno-Martos et al. 2024 | hospitalisation mental health population | 0.71 | 0.03 | [0.66 – 0.76] | Europe | Norway | A | 6 | 01.03.2020 | 31.03.2020 | 32.62 | 17.86 | [9.52 – 53.57] |
|  |  | Moreno-Martos et al. 2024 | hospitalisation mental health population | 0.92 | 0.02 | [0.88 – 0.97] | Europe | Sweden | A | 6 | 01.03.2020 | 31.03.2020 | 33.98 | 11.20 | [13.10 – 48.81] |
|  |  | Clerici et al. 2020 | all psych. inpatient admissions | 0.93 | 0.05 | [0.84 – 1.04] | Europe | Italy | B | 6 | 01.01.2020 | 31.03.2020 | 34.39 | 30.06 | [0.00 – 73.81] |
|  |  | Boldrini et al. 2021 | all psych. inpatient admissions | 0.78 | 0.03 | [0.73 – 0.84] | Europe | Italy | B | 6 | 01.03.2020 | 30.06.2020 | 73.25 | 7.00 | [56.85 – 85.42] |
|  |  | Fasshauer et al. 2021a | psych. emergency hospital admissions | 0.70 | 0.02 | [0.67 – 0.73] | Europe | Germany | B | 6 | 13.03.2020 | 21.05.2020 | 61.03 | 8.79 | [33.04 – 70.83] |
|  |  | Zielasek et al. 2021 | all psych. inpatient admissions | 0.75 | 0.01 | [0.73 – 0.77] | Europe | Germany | B | 6 | 18.03.2020 | 31.05.2020 | 62.63 | 5.29 | [41.96 – 70.83] |
|  |  | Bakolis et al. 2021 | all psych. inpatient admissions | 1.03 | 0.02 | [1.02 – 1.04] | Europe | United Kingdom | B | 6 | 23.03.2020 | 09.06.2020 | 60.76 | 1.89 | [56.25 – 66.96] |
|  |  | Wettstein et al. 2022 | all psych. inpatient admissions | 0.38 | 0.03 | [0.36 – 0.40] | Africa | South Africa | B | 6 | 30.03.2020 | 31.05.2020 | 79.08 | 1.86 | [75.60 – 80.95] |
|  |  | Fasshauer et al. 2022 | psych. emergency hospital admissions | 0.88 | 0.01 | [0.87 – 0.90] | Europe | Germany | B | 6 | 01.04.2020 | 30.06.2020 | 62.33 | 3.79 | [57.44 – 70.83] |
|  |  | Pignon et al. 2020 | hospitalization after psychiatric emergency attendance | 0.49 | 0.13 | [0.38 – 0.63] | Europe | France | B | 6 | 17.03.2020 | 13.04.2020 | 71.43 | - | [71.43 – 71.43] |
|  |  | Balestrieri et al. 2021 | hospitalization after psychiatric emergency attendance | 0.60 | 0.06 | [0.53 – 0.67] | Europe | Italy | B | 6 | 08.03.2020 | 14.05.2020 | 77.68 | 5.84 | [65.77 – 85.42] |
|  |  | Mehrabadi et al. 2024 | patients admitted to the hospitals | 1.01 | 0.00 | [0.99 – 1.02] | Americas | USA | B | 6 | 01.03.2020 | 31.10.2020 | 61.25 | 9.42 | [19.05 – 64.88] |
|  | Rand. Effects Model A, B |  |  | **0.75** |  | **[0.67 – 0.85]** |  |  |  |  |  |  |  |  |  |
|  |  | Golubovic et al. 2022 | all psych. inpatient admissions | 1.07 | 0.14 | [0.81 – 1.42] | Europe | Serbia | C | 5 | 01.05.2020 | 31.08.2020 | 48.04 | 11.14 | [27.38 – 78.57] |
|  |  | Panariello et al. 2021 | all psych. inpatient admissions | 0.58 | 0.18 | [0.41 – 0.83] | Europe | Italy | C | 5 | 24.02.2020 | 30.04.2020 | 74.92 | 8.83 | [56.85 – 85.42] |
|  |  | Jagadheesan et al. 2021 a | all psych. inpatient admissions | 0.88 | 0.04 | [0.82 – 0.95] | Western Pacific | Australia | C | 5 | 16.03.2020 | 16.09.2020 | 61.28 | 29.17 | [72.62 – 9.05] |
|  |  | Yang et al. 2022 | all psych. inpatient admissions | 0.15 | 0.20 | [0.10 – 0.21] | Western Pacific | China | C | 5 | 01.01.2020 | 30.04.2020 | 56.89 | 21.47 | [6.85 – 73.21] |
|  |  | Lee et al. 2020 | all psych. inpatient admissions | 1.21 | 0.12 | [0.96 - 1.54] | Western Pacific | China | C | 5 | 01.02.2020 | 30.04.2020 | 67.58 | 5.32 | [59.23 – 73.21] |
|  |  | Nejati et al. 2021 | all psych. inpatient admissions | 0.97 | 0.10 | [0.80 – 1.19] | Americas | Canada | C | 5 | 22.03.2020 | 05.06.2020 | 62.37 | 0.58 | [61.31 – 63.39] |
|  |  | Gómez-Ramiro et al. 2021 | hospitalization after psychiatric emergency attendance | 0.91 | 0.09 | [0.77 – 1.08] | Europe | Spain | C | 5 | 14.03.2020 | 12.06.2020 | 61.00 | 4.79 | [49.11 – 67.86] |
|  |  | Sobetzko et al. 2021 | hospitalization after psychiatric emergency attendance | 1.17 | 0.10 | [0.98 – 1.40] | Europe | Germany | C | 6 | 15.03.2020 | 04.05.2020 | 62.01 | 8.49 | [33.04 – 70.83] |
|  |  | Ambrosetti et al. 2021 | hospitalization after psychiatric emergency attendance | 0.88 | 0.11 | [0.71 – 1.08] | Europe | Switzerland | C | 5 | 01.04.2020 | 15.05.2020 | 59.19 | 1.13 | [57.14 – 60.12] |
|  |  | Alves et al. 2021 | hospitalization after psychiatric emergency attendance | 1.00 | 0.47 | [0.40 – 2.25] | Europe | Portugal | C | 5 | 18.03.2020 | 02.05.2020 | 68.28 | 2.89 | [52.38 – 72.02] |
|  |  | Seifert et al. 2021 | hospitalization after psychiatric emergency attendance | 0.67 | 0.09 | [0.56 – 0.80] | Europe | Germany | C | 5 | 16.03.2020 | 24.05.2020 | 62.07 | 6.56 | [38.99 – 70.83] |
|  |  | Montalbani et al. 2021 | hospitalization after psychiatric emergency attendance | 0.85 | 0.24 | [0.53 – 1.36] | Europe | Italy | C | 5 | 12.03.2020 | 03.05.2020 | 79.37 | 5.69 | [72.02 – 85.42] |
|  |  | Di Lorenzo et al. 2021b | hospitalization after psychiatric emergency attendance | 0.64 | 0.13 | [0.50 – 0.82] | Europe | Italy | C | 5 | 01.03.2020 | 31.08.2020 | 70.76 | 6.71 | [56.85 – 85.42] |
|  |  | Goldschmidt et al. 2023 | hospital admission after ED contact | 0.83 | 0.07 | [0.70 – 0.97] | Europe | Germany | C | 5 | 02.03.2020 | 24.05.2020 | 56.79 | 13.39 | [27.98 – 70.83] |
|  |  | Irigoyen-Otiñano et al. 2024a | hospital admission after ED contact | 1.09 | 0.10 | [0.10 – 1.29] | Europe | Spain | C | 5 | 14.03.2020 | 21.06.2020 | 60.18 | 5.40 | [42.56 – 67.86] |
|  |  | Dindar et al. 2024 | psych. emergency hospital admissions, hospitalization | 0.71 | 0.22 | [0.30 – 1.13] | Europe | Turkey | C | 5 | 10.03.2020 | 10.09.2020 | 58.73 | 8.79 | [23.21 – 67.86] |
|  |  | Kim et al. 2023b | all psychiatric inpatient admissions | 1.07 | 0.05 | [0.96 – 1.17] | Americas | USA | C | 5 | 13.03.2020 | 03.07.2020 | 62.64 | 5.60 | [32.14 – 64.88] |
|  |  | Qamruddin et al. 2022 | inpatient psychiatric care | 1.78 | 0.10 | [1.60 – 1.96] | Eastern Mediterranean | United Arab Emirates | C | 5 | 11.03.2020 | 11.06.2020 | 68.79 | 13.83 | [30.36 – 83.93] |
|  |  | Hamlin et al. 2022 | admission rates at the psychiatric units | 0.95 | 0.13 | [0.68 – 1.21] | Europe | Sweden | C | 5 | 10.03.2020 | 14.06.2020 | 51.50 | 7.53 | [22.02 – 57.14] |
|  |  | Irigoyen-Otiñano et al. 2024b | all hospital admissions | 1.07 | 0.22 | [0.63 – 1.50] | Europe | Spain | C | 5 | 15.03.2020 | 20.06.2020 | 60.48 | 5.02 | [49.11 – 67.86] |
|  |  | Cafaro et al. 2022 | psychiatric hospitalization after ED contact | 0.59 | 0.11 | [0.36 – 0.81] | Europe | Italy | C | 5 | 01.03.2020 | 31.05.2020 | 75.27 | 6.94 | [59.23 – 85.42] |
|  |  | deDiegoRuiz et al. 2023 | all admissions to the acute psychiatry | 1.05 | 0.10 | [0.85 – 1.24] | Europe | Spain | C | 5 | 15.03.2020 | 30.05.2020 | 61.89 | 4.50 | [49.11 – 67.86] |
|  |  | Ross et al. 2023 | psychiatric admissions through the ED | 1.92 | 0.12 | [1.68 – 2.16] | Americas | USA | C | 5 | 01.03.2020 | 31.10.2020 | 61.25 | 9.42 | [19.05 – 64.88] |
|  | Rand. Effects Model C |  |  | **0.88** |  | **[0.73 – 1.07]** |  |  |  |  |  |  |  |  |  |
| inpatient  long term |  | Engels et al. 2022 | all psych. inpatient admissions | 0.86 | 0.00 | [0.86 – 0.87] | Europe | Germany | A | 6 | 01.01.2020 | 31.12.2020 | 50.51 | 19.92 | [0.00 – 73.69] |
|  |  | Romer et al. 2021 | all psych. inpatient admissions | 0.98 | 0.01 | [0.96 – 1.00] | Europe | Denmark | A | 6 | 01.01.2020 | 31.12.2020 | 42.85 | 19.96 | [0.00 – 61.9] |
|  |  | Kim et al. 2023a | inpatient treatment | 0.85 | 0.01 | [0.03 – 0.03] | Western Pacific | South Korea | A | 6 | 01.01.2020 | 31.12.2020 | 50.10 | 17.33 | [0.00 – 73.81] |
|  |  | Lieber et al. 2024 | use of inpatient mental health services | 0.91 | 0.07 | [0.77 - 1.05] | Europe | Sweden | A | 6 | 01.01.2020 | 31.12.2020 | 44.38 | 18.28 | [0.00 – 61.70] |
|  |  | Hansen et al. 2024 | all hospitalizations | 1.00 | 0.00 | [0.97 – 1.01] | Western Pacific | New Zealand | A | 6 | 01.01.2020 | 31.12.2020 | 37.62 | 21.28 | [0.00 – 80.95] |
|  |  | Chu et al. 2024 | all hospitalizations | 1.02 | 0.01 | [0.99 – 1.05] | Americas | Canada | A | 6 | 01.06.2021 | 01.06.2022 | 60.41 | 12.69 | [32.77 – 73.21] |
|  |  | Fellinger et al. 2023 | admissions to a psychiatric hospital | 0.96 | 0.00 | [0.94 – 0.97] | Europe | Austria | A | 6 | 01.01.2020 | 31.12.2020 | 48.10 | 24.64 | [0.00 – 82.62] |
|  |  | Rachamin et al. 2023 | all psychiatric admissions | 1.01 | 0.00 | [1.00 – 1.01] | Europe | Switzerland | A | 6 | 01.01.2020 | 31.12.2020 | 43.81 | 20.13 | [0.00 – 63.33] |
|  |  | Moreno-Martos et al. 2024 | hospitalisation mental health population | 1.03 | 0.00 | [1.02 – 1.04] | Europe | Norway | A | 6 | 01.03.2020 | 31.12.2020 | 42.62 | 9.80 | [9.52 – 57.14] |
| **Table 2 (cont.) – Page 2** |  | Moreno-Martos et al. 2024 | hospitalisation mental health population | 1.00 | 0.00 | [0.99 – 1.00] | Europe | Sweden | A | 6 | 01.03.2020 | 31.12.2020 | 51.81 | 7.33 | [13.10 – 61.79] |
|  |  | Fasshauer et al. 2021b | all psych. inpatient admissions | 0.88 | 0.01 | [0.87 – 0.90] | Europe | Germany | B | 6 | 01.01.2020 | 31.12.2020 | 50.51 | 19.92 | [0.00 – 73.69] |
|  |  | Fasshauer et al. 2022 | psych. emergency hospital admissions | 0.88 | 0.01 | [0.87 – 0.90] | Europe | Germany | B | 6 | 01.01.2020 | 31.12.2020 | 50.51 | 19.92 | [0.00 – 73.69] |
|  |  | Simpson et al. 2021 | hospitalization after psychiatric emergency attendance | 0.95 | 0.02 | [0.91 – 0.98] | Americas | USA | B | 6 | 01.01.2020 | 31.12.2020 | 53.49 | 21.64 | [0 – 69.29] |
|  |  | Abe et al. 2025 | number of hospitalizations | 0.75 | 0.02 | [0.72 – 0.79] | Western Pacific | Japan | B | 6 | 01.04.2020 | 31.12.2020 | 43.17 | 3.98 | [35.12 – 50.00] |
|  | Rand. Effects Model A, B |  |  | **0.93** |  | **[0.89 – 0.98]** |  |  |  |  |  |  |  |  |  |
|  |  | Berardelli et al.  2021 | all psych. inpatient admissions | 1.44 | 0.08 | [1.23 – 1.68] | Europe | Italy | C | 5 | 10.03.2020 | 31.12.2020 | 70.93 | 6.15 | [62.20 – 85.42] |
|  |  | Vukojevic et al. 2021 | psych. emergency hospital admissions | 0.69 | 0.03 | [0.66 – 0.73] | Europe | Croatia | C | 6 | 01.02.2020 | 30.11.2020 | 49.92 | 17.40 | [7.14 – 80.95] |
|  |  | Wullschleger et al. 2023 | all psych. inpatient admissions | 0.89 | 0.01 | [0.86 – 0.91] | Europe | Switzerland | C | 5 | 01.01.2020 | 31.12.2020 | 43.81 | 20.13 | [0.00 – 63.33] |
|  |  | Dindar et al. 2024 | psych. emergency hospital admissions, hospitalization | 0.88 | 0.17 | [0.53 – 1.23] | Europe | Turkey | C | 5 | 10.03.2020 | 10.03.2021 | 65.48 | 9.88 | [23.21 – 80.83] |
|  |  | Perozziello et al. 2023 | number of hospital admissions | 0.84 | 0.01 | [0.81 – 0.87] | Europe | France | C | 5 | 01.01.2020 | 31.12.2020 | 51.67 | 21.14 | [0.00 – 72.32} |
|  |  | Gajdics et al. 2023 | number of inpatient admissions | 0.59 | 0.06 | [0.46 – 0.73] | Europe | Hungary | C | 5 | 11.03.2020 | 31.05.2022 | 50.46 | 14.15 | [22.62 – 76.31] |
|  |  | Savić et al. 2022 | inpatient hospitalizations | 0.71 | 0.01 | [0.67 – 0.74] | Europe | Kroatia | C | 5 | 01.01.2020 | 31.12.2020 | 47.00 | 21.69 | [0.00 – 80.95] |
|  |  | Russolillo et al. 2024 | any psychiatric hospital admission | 0.99 | 0.06 | [0.86 – 1.11] | Americas | Canada | C | 5 | 01.03.2020 | 31.12.2020 | 60.43 | 11.14 | [7.74 – 68.33] |
|  |  | Vukićević et al. 2025 | psychiatric hospital admissions | 0.72 | 0.03 | [0.64 – 0.80] | Europe | Croatia | C | 5 | 01.03.2020 | 01.03.2021 | 54.55 | 13.16 | [18.45 – 80.95] |
|  |  | Warwicker et al. 2023 | psychiatric admissions | 1.02 | 0.03 | [0.94 – 1.09] | Europe | Malta | C | 5 | 01.01.2020 | 31.12.2020 | 49.69 | 22.38 | [0.00 – 80.95] |
|  |  | Zaki et al. 2022 | admissions to acute psychiatric units | 0.94 | 0.03 | [0.87 – 0.99] | Western Pacific | Australia | C | 5 | 01.01.2020 | 31.12.2020 | 52.36 | 21.19 | [0.00 – 72.62] |
|  | Rand. Effects Model C |  |  | **0.86** |  | **[0.74 – 0.99]** |  |  |  |  |  |  |  |  |  |
| **emergency department short term**  **cut-off: 8 months** |  |  |  |  |  |  |  |  |  |  |  |  |  |  |  |
|  |  | Jones et al. 2024 | emergency department presentations | 1.14 | 0.00 | [1.11 – 1.15] | Western Pacific | Australia | A | 6 | 09.03.2020 | 28.06.2020 | 53.55 | 10.61 | [22.02 – 62.50] |
|  |  | Anderson et al. 2022 | emergency department contacts | 0.91 | 0.00 | [0.91 – 0.92] | Americas | USA | A | 6 | 14.02.2021 | 13.03.2021 | 66.64 | 4.61 | [17.26 – 32.14] |
|  |  | Beghi et al. 2022 | emergency department contacts | 1.11 | 0.04 | [1.03 – 1.20] | Europe | Italy | A | 5 | 04.05.2020 | 31.08.2020 | 67.45 | 2.69 | [63.99 – 75.89] |
|  |  | Holland et al. 2021 | emergency department contacts | 0.99 | 0.01 | [0.98 – 1.01] | Americas | USA | A | 6 | 15.03.2020 | 10.10.2020 | 63.58 | 3.11 | [39.29 – 64.88] |
|  |  | Lee et al. 2022 | emergency department contacts | 0.83 | 0.01 | [0.82 – 0.84] | Western Pacific | Republic of  Korea | B | 6 | 27.01.2020 | 29.06.2020 | 50.88 | 15.26 | [2.38 – 73.81] |
|  |  | Balestrieri et al. 2021 | emergency department contacts | 0.65 | 0.04 | [0.60 – 0.70] | Europe | Italy | B | 6 | 08.03.2020 | 14.05.2020 | 77.68 | 5.84 | [65.77 – 85.42] |
|  |  | Pignon et al. 2020 | emergency department contacts | 0.45 | 0.05 | [0.41 – 0.50] | Europe | France | B | 6 | 17.03.2020 | 13.04.2020 | 71.43 | - | [71.43 – 71.43] |
|  |  | Wang et al. 2024 | emergency department utilization | 1.20 | 0.00 | [1.18 – 1.21] | Americas | USA | B | 6 | 02.05.2020 | 31.12.2020 | 64.61 | 2.04 | [61.90 – 69.29] |
|  | Rand. Effects Model A, B |  |  | **0.87** |  | **[0.69 – 1.10]** |  |  |  |  |  |  |  |  |  |
|  |  | Sobetzko et al. 2021 | emergency department contacts | 1.03 | 0.07 | [0.90 – 1.19} | Europe | Germany | C | 6 | 15.03.2020 | 04.05.2020 | 62.01 | 8.49 | [33.04 – 70.83] |
|  |  | Stein et al. 2020 | emergency department contacts | 0.76 | 0.05 | [0.69 – 0.85] | Europe | Italy | C | 6 | 13.01.2020 | 03.05.2020 | 52.45 | 31.41 | [0 – 85.42] |
|  |  | Yalcin et al. 2021 | emergency department contacts | 0.82 | 0.03 | [0.78 – 0.87] | Europe | Turkey | C | 5 | 30.03.2020 | 31.05.2020 | 64.49 | 1.62 | [61.9 – 67.86] |
|  |  | Alves et al. 2021 | emergency department contacts | 0.45 | 0.10 | [0.38 – 0.55] | Europe | Portugal | C | 5 | 18.03.2020 | 02.05.2020 | 68.28 | 2.89 | [52.38 – 72.02] |
|  |  | Ambrosetti et al. 2021 | emergency department contacts | 0.82 | 0.06 | [0.74 – 0.92] | Europe | Switzerland | C | 5 | 01.04.2020 | 15.05.2020 | 59.19 | 1.13 | [57.14 – 60.12] |
|  |  | Capuzzi et al. 2020 | emergency department contacts | 0.56 | 0.09 | [0.47 – 0.67] | Europe | Italy | C | 5 | 21.02.2020 | 03.05.2020 | 70.77 | 6.69 | [59.23 – 85.42] |
|  |  | Di Lorenzo et al. 2021b | emergency department contacts | 0.79 | 0.06 | [0.70 – 0.89] | Europe | Italy | C | 5 | 01.03.2020 | 31.08.2020 | 70.76 | 6.71 | [56.85 – 85.42] |
|  |  | Flament et al. 2021 | emergency department contacts | 0.91 | 0.20 | [0.62 – 1.33] | Europe | Belgium | C | 4 | 01.05.2020 | 31.05.2020 | 67.08 | 1.59 | [64.88 – 69.05] |
|  |  | Gómez-Ramiro et al. 2021 | emergency department contacts | 0.62 | 0.05 | [0.57 – 0.68] | Europe | Spain | C | 5 | 14.03.2020 | 12.06.2020 | 61.00 | 4.79 | [49.11 – 67.86] |
|  |  | Goncalves-Pinho et al. 2020 | emergency department contacts | 0.48 | 0.04 | [0.44 – 0.52] | Europe | Portugal | C | 5 | 19.03.2020 | 02.05.2020 | 68.64 | 1.70 | [66.07 – 72.02] |
|  |  | Jagadheesan et al. 2021 b | emergency department contacts | 0.97 | 0.07 | [0.85 – 1.10] | Western Pacific | Australia | C | 5 | 16.03.2020 | 16.09.2020 | 61.28 | 9.08 | [29.17 – 72.62] |
|  |  | McAndrew et al. 2021 | emergency department contacts | 0.79 | 0.31 | [0.43 – 1.45] | Europe | Ireland | C | 5 | 16.03.2020 | 10.05.2020 | 64.00 | 13.27 | [36.90 – 71.43] |
|  |  | McDowell et al. 2021 | emergency department contacts | 0.64 | 0.07 | [0.56 – 0.73] | Americas | USA | C | 5 | 09.03.2020 | 17.05.2020 | 59.42 | 10.40 | [25.00 – 64.88] |
|  |  | Montalbani et al. 2021 | emergency department contacts | 0.62 | 0.16 | [0.46 – 0.85] | Europe | Italy | C | 5 | 12.03.2020 | 03.05.2020 | 79.37 | 5.69 | [72.02 – 85.42] |
|  |  | Pikkel Igal et al. 2021 | emergency department contacts | 0.71 | 0.05 | [0.64 – 0.79] | Europe | Israel | C | 5 | 01.03.2020 | 30.04.2020 | 64.27 | 21.01 | [14.88 – 82.74] |
|  |  | Seifert et al. 2021 | emergency department contacts | 0.79 | 0.07 | [0.69 – 0.90] | Europe | Germany | C | 5 | 16.03.2020 | 24.05.2020 | 62.07 | 6.56 | [38.99 – 70.83] |
|  |  | Goldschmidt et al. 2023 | emergency psychiatric presentations | 0.91 | 0.05 | [0.81 – 1.00] | Europe | Germany | C | 5 | 02.03.2020 | 24.05.2020 | 56.79 | 13.39 | [27.98 – 70.83] |
|  |  | Irigoyen-Otiñano et al. 2024a | emergency department contacts | 0.86 | 0.05 | [0.76 – 0.96] | Europe | Spain | C | 5 | 14.03.2020 | 21.06.2020 | 60.18 | 5.40 | [42.56 – 67.86] |
|  |  | Muştucu et al. 2023 | admission in emergency department | 1.03 | 0.07 | [0.88 – 1.17] | Europe | Turkey | C | 5 | 11.03.2020 | 01.09.2020 | 59.13 | 8.53 | [23.21 – 67.86] |
|  |  | Hamlin et al. 2022 | psychiatric emergency room visits | 0.85 | 0.10 | [0.64 – 1.04] | Europe | Sweden | C | 5 | 10.03.2020 | 14.06.2020 | 51.50 | 7.53 | [22.02 – 57.14] |
|  |  | Irigoyen-Otiñano et al. 2024b | number of visits to the emergency department | 1.02 | 0.09 | [0.82 – 1.20] | Europe | Spain | C | 5 | 15.03.2020 | 20.06.2020 | 60.48 | 5.02 | [49.11 – 67.86] |
|  |  | Bruckner et al. 2023 | number of psychiatric emergency visits | 0.77 | 0.01 | [0.73 – 0.79] | Americas | USA | C | 5 | 13.03.2020 | 07.05.2020 | 60.73 | 7.47 | [32.14 – 64.88] |
|  |  | Cafaro et al. 2022 | psychiatric emergency department visits | 0.51 | 0.06 | [0.39 – 0.62] | Europe | Italy | C | 5 | 01.03.2020 | 31.05.2020 | 75.27 | 6.94 | [59.23 – 85.42] |
|  | Rand. Effects Model C |  |  | **0.75** |  | **[0.68 – 0.83]** |  |  |  |  |  |  |  |  |  |
| emergency department long term |  |  |  |  |  |  |  |  |  |  |  |  |  |  |  |
|  |  | Chu et al. 2024 | emergency department visits | 1.00 | 0.01 | [0.98 – 1.01] | Americas | Canada | A | 6 | 01.06.2021 | 01.06.2022 | 60.41 | 12.69 | [32.77 – 73.21] |
|  |  | Molina et al. 2022 | emergency department encounters | 0.75 | 0.02 | [0.72 – 0.79] | Americas | USA | A | 5 | 01.01.2020 | 31.12.2020 | 53.49 | 21.67 | [0.00 – 69.29] |
|  |  | Mukadam et al. 2021 | emergency department contacts | 0.86 | 0.02 | [0.83 – 0.88} | Europe | United Kingdom | B | 6 | 01.01.2020 | 31.12.2020 | 51.80 | 22.15 | [0.00 – 76.31] |
|  |  | Simpson et al. 2021 | emergency department contacts | 0.94 | 0.01 | [0.92 – 0.95] | Americas | USA | B | 6 | 01.01.2020 | 31.12.2020 | 53.49 | 21.64 | [0.00 – 69.29] |
|  |  | Ramadan et al. 2022 | emergency department contacts | 3.78 | 0.07 | [3.30 – 4.33] | Eastern Mediterranean | Saudi-Arabia | B | 6 | 01.01.2020 | 31.05.2021 | 57.24 | 20.51 | [0.00 – 83.33] |
|  | Rand. Effects Model A, B |  |  | **1.18** |  | **[0.66 – 2.09]** |  |  |  |  |  |  |  |  |  |
|  |  | Hakansson et al. 2021 | emergency department contacts | 0.88 | 0.02 | [0.85 – 0.92] | Europe | Sweden | C | 5 | 01.03.2020 | 31.12.2020 | 51.81 | 7.31 | [13.1 – 61.79] |
|  |  | Zhang et al. 2023 | psychiatric emergency departments visits | 1.77 | 0.10 | [1.56 – 1.98] | Western Pacific | China | C | 5 | 01.01.2020 | 31.12.2020 | 65.81 | 15.05 | [6.85 – 77.98] |
|  |  | Perozziello et al. 2023 | psychiatric emergency departments visits | 0.80 | 0.02 | [0.76 – 0.83] | Europe | France | C | 5 | 01.01.2020 | 31.12.2020 | 51.67 | 21.14 | [0.00 – 72.32} |
|  |  | Giménez-Palomo et al. 2024 | admission to the psychiatric emergency service | 0.83 | 0.02 | [0.79 – 0.87] | Europe | Spain | C | 5 | 01.01.2020 | 31.12.2020 | 49.24 | 20.52 | [0.00 – 71.31] |
|  |  | Savić et al. 2022 | emergency department visits | 1.46 | 0.04 | [1.37 – 1.54] | Europe | Kroatia | C | 5 | 01.01.2020 | 31.12.2020 | 47.00 | 21.69 | [0.00 – 80.95] |
|  |  | Akkaoui et al. 2025 | psychiatric emergency visits | 0.76 | 0.09 | [0.63 – 0.91] | Europe | France | C | 5 | 01.01.2020 | 31.12.2020 | 51.67 | 21.14 | [0.00 – 72.32] |
|  | Rand. Effects Model C |  |  | **1.02** |  | **[0.77 – 1.35]** |  |  |  |  |  |  |  |  |  |
| **outpatient short term**  **cut-off: 8 months** |  |  |  |  |  |  |  |  |  |  |  |  |  |  |  |
|  |  | Chen et al. 2020a | outpatient contacts to mental health services | 0.70 | 0.11 | [0.56 – 0.87] | Europe | United Kingdom | A | 6 | 23.03.2020 | 19.05.2020 | 60.43 | 1.40 | [56.25 – 61.61] |
|  |  | Mangiapane et al. 2022 | outpatient contacts to mental health services | 0.97 | 0.00 | [0.97 – 0.98] | Europe | Germany | A | 6 | 01.01.2020 | 31.03.2020 | 38.04 | 25.51 | [0.00 – 70.83] |
|  |  | Villarreal-Zegarra et al. 2023 | outpatient contacts to mental health services | 0.88 | 0.01 | [0.87 – 0.90] | Americas | Peru | A | 6 | 01.03.2020 | 31.03.2020 | 46.91 | 30.16 | [2.38 – 73.81] |
|  |  | Ahmedani et al. 2024 | outpatient psychotherapy visits in person | 0.47 | 0.00 | [0.47 – 0.48] | Americas | USA | A | 6 | 14.03.2020 | 31.12.2020 | 63.98 | 3.59 | [35.71 – 69.29] |
|  |  | Baum et al. 2024 | outpatient mental health care system | 1.01 | 0.02 | [0.96 – 1.04] | Europe | Germany | A | 6 | 01.03.2020 | 31.05.2020 | 56.52 | 13.06 | [27.98 – 70.83] |
|  |  | Joo et al. 2022 | number of psychiatric outpatients | 0.97 | 0.00 | [0.97 – 0.97] | Western Pacific | South Korea | A | 6 | 01.02.2020 | 31.08.2020 | 53.05 | 10.94 | [14.88 – 73.81] |
|  |  | Zhang et al. 2022 | mental health outpatient care | 0.89 | 0.00 | [0.88 – 0.89] | Americas | USA | A | 6 | 18.03.2020 | 05.05.2020 | 62.64 | 3.52 | [48.21 – 64.88] |
|  |  | Carr et al. 2021 | primary care data from general practicers | 0.78 | 0.00 | [0.77 – 0.78] | Europe | United Kingdom | A | 6 | 01.03.2020 | 30.09.2020 | 57.85 | 13.84 | [9.52 – 67.56] |
|  |  | Patel et al. 2021 | outpatient contacts to mental health services | 0.96 | 0.00 | [0.95 – 0.96] | Europe | United Kingdom | A | 6 | 01.03.2020 | 30.06.2020 | 54.13 | 17.34 | [9.25 – 67.56] |
|  |  | Raventos et al. 2022 | primary care data from general practicers | 1.06 | 0.01 | [1.04 – 1.07] | Europe | Spain | A | 6 | 01.03.2020 | 30.06.2020 | 54.67 | 13.91 | [13.1 – 67.86] |
|  |  | Sanchez-Guarnido et al. 2022 | outpatient contacts to mental health services | 0.85 | 0.14 | [0.65 – 1.12] | Europe | Spain | B | 6 | 16.03.2020 | 16.05.2020 | 61.75 | 4.50 | [50.3 – 67.86] |
|  |  | Wettstein et al. 2022 | outpatient contacts to mental health services | 0.74 | 0.06 | [0.66 – 0.83] | Africa | South Africa | B | 6 | 30.03.2020 | 31.05.2020 | 79.08 | 1.86 | [75.60 – 80.95] |
|  |  | Williams et al. 2020 | primary care data from general practicers | 0.50 | 0.04 | [0.46 – 0.54] | Europe | United Kingdom | B | 6 | 01.03.2020 | 31.05.2020 | 50.26 | 18.35 | [9.25 – 62.2] |
|  |  | Simkin et al. 2022 | referrals to mental health services | 0.37 | 0.05 | [0.26 – 0.46] | Europe | United Kingdom | B | 6 | 16.03.2020 | 05.07.2020 | 59.96 | 9.80 | [13.10 – 67.56] |
|  |  | Bakolis et al. 2021 | mental health service use | 1.03 | 0.00 | [1.02 – 1.04] | Europe | United Kingdom | B | 6 | 23.03.2020 | 09.06.2020 | 60.76 | 1.89 | [56.25 – 66.96] |
|  | Rand. Effects Model A, B |  |  | **0.78** |  | **[0.66 – 0.92]** |  |  |  |  |  |  |  |  |  |
|  |  | Andersson et al. 2022 | outpatient contacts to mental health services | 0.84 | 0.07 | [0.74 – 0.95] | Europe | Sweden | C | 5 | 01.03.2020 | 31.05.2020 | 47.74 | 11.74 | [13.1 – 54.76] |
|  |  | Chow et al. 2021 | outpatient contacts to mental health services | 1.05 | 0.01 | [1.03 – 1.06] | Europe | Netherlands | C | 5 | 01.04.2020 | 30.06.2020 | 60.58 | 3.11 | [55.59 – 63.69] |
|  |  | Di Lorenzo et al. 2021a | outpatient contacts to mental health services | 1.15 | 0.07 | [1.01 – 1.31] | Europe | Italy | C | 5 | 01.03.2020 | 31.08.2020 | 70.76 | 6.71 | [56.85 – 85.42] |
|  |  | Lee et al. 2020 | outpatient contacts to mental health services | 1.02 | 0.04 | [0.95 – 1.09] | Western Pacific | China | C | 5 | 01.02.2020 | 30.04.2020 | 67.58 | 5.32 | [59.23 – 73.21] |
|  |  | Seo et al. 2021 | outpatient contacts to mental health services | 0.86 | 0.01 | [0.84 – 0.88] | Western Pacific | Republic of Korea | C | 5 | 01.03.2020 | 31.05.2020 | 56.83 | 10.04 | [47.32 – 73.81] |
|  |  | Irigoyen-Otiñano et al. 2024a | referral to outpatient mental health clinic, appointment | 0.79 | 0.06 | [0.67 – 0.91] | Europe | Spain | C | 5 | 14.03.2020 | 21.06.2020 | 60.18 | 5.40 | [42.56 – 67.86] |
|  |  | Jahlan et al. 2022 | psychiatric outpatient department | 0.49 | 0.14 | [0.20 – 0.77] | Eastern Mediterranean | Saudi-Arabia | C | 5 | 22.03.2020 | 21.06.2020 | 76.91 | 2.39 | [63.10 – 83.33] |
|  | Rand. Effects Model C |  |  | **0.88** |  | **[0.73 – 1.05]** |  |  |  |  |  |  |  |  |  |
| outpatient  long term |  | Kim et al. 2023a | outpatient treatment | 1.03 | 0.00 | [0.03 – 0.03] | Western Pacific | South Korea | A | 6 | 01.01.2020 | 31.12.2020 | 50.10 | 17.33 | [0.00 – 73.81] |
|  |  | Lieber et al. 2024 | use of outpatient mental health services | 0.96 | 0.03 | [0.90 – 1.02] | Europe | Sweden | A | 6 | 01.01.2020 | 31.12.2020 | 44.38 | 18.28 | [0.00 – 61.70] |
|  |  | Silva-Valencia et al. 2024 | in person visits | 0.74 | 0.00 | [0.72 – 0.75] | Americas | Argentina | A | 6 | 01.04.2020 | 28.02.2021 | 76.10 | 3.79 | [70.54 – 86.31] |
|  |  | Silva-Valencia et al. 2024 | in person visits | 0.60 | 0.00 | [0.59 – 0.61] | Western Pacific | Australia | A | 6 | 01.04.2020 | 28.02.2021 | 62.51 | 6.92 | [46.73 – 74.40] |
|  |  | Silva-Valencia et al. 2024 | in person visits | 0.16 | 0.01 | [0.14 – 0.17] | Americas | Canada | A | 6 | 01.04.2020 | 28.02.2021 | 64.47 | 3.64 | [59.52 – 71.55] |
|  |  | Silva-Valencia et al. 2024 | in person visits | 0.75 | 0.00 | [0.74 – 0.75] | Europe | Norway | A | 6 | 01.04.2020 | 28.02.2021 | 47.66 | 11.08 | [33.63 – 69.23] |
|  |  | Silva-Valencia et al. 2024 | in person visits | 0.95 | 0.00 | [0.95 – 0.96] | Americas | Peru | A | 6 | 01.04.2020 | 28.02.2021 | 73.42 | 6.24 | [55.36 – 82.74] |
|  |  | Silva-Valencia et al. 2024 | in person visits | 1.11 | 0.01 | [1.08 – 1.14] | Western Pacific | Singapore | A | 6 | 01.04.2020 | 28.02.2021 | 62.77 | 7.50 | [45.83 – 79.17] |
|  |  | Silva-Valencia et al. 2024 | in person visits | 0.81 | 0.00 | [0.80 – 0.82] | Europe | Sweden | A | 6 | 01.04.2020 | 28.02.2021 | 55.55 | 4.32 | [51.19 – 64.42] |
|  |  | Chu et al. 2024 | outpatient visits | 1.05 | 0.00 | [1.04 – 1.06] | Americas | Canada | A | 6 | 01.06.2021 | 01.06.2022 | 60.41 | 12.69 | [32.77 – 73.21] |
|  |  | Flodin et al. 2023 | primary care utilization | 0.99 | 0.01 | [0.96 – 1.02] | Europe | Norway | A | 6 | 01.03.2020 | 31.12.2020 | 42.62 | 9.80 | [9.52 – 57.14] |
|  |  | Flodin et al. 2023 | primary care utilization | 1.20 | 0.02 | [1.14 – 1.26] | Europe | Latvia | A | 6 | 01.03.2020 | 31.12.2020 | 45.50 | 8.75 | [7.74 – 59.76] |
|  |  | Flodin et al. 2023 | primary care utilization | 0.91 | 0.00 | [0.90 – 0.93] | Europe | Sweden | A | 6 | 01.03.2020 | 31.12.2020 | 51.81 | 7.33 | [13.10 – 61.79] |
|  |  | Flodin et al. 2023 | primary care utilization | 0.97 | 0.03 | [0.91 – 1.03] | Europe | Netherlands | A | 6 | 01.03.2020 | 31.12.2020 | 54.77 | 9.82 | [13.10 – 67.26] |
|  |  | Rice et al. 2025 | any mental health visits | 1.11 | 0.00 | [1.10 – 1.11] | Americas | USA | A | 6 | 11.03.2020 | 10.12.2020 | 63.26 | 4.88 | [26.79 – 67.86] |
|  |  | Li et al. 2023 | outpatient department utilization | 1.01 | 0.00 | [1.00 – 1.01] | Western Pacific | China | B | 6 | 01.01.2020 | 31.12.2020 | 65.81 | 15.05 | [6.85 – 77.98] |
|  |  | Rachamin et al. 2023 | all psychotherapy consultations | 0.96 | 0.03 | [0.88 – 1.03] | Europe | Switzerland | B | 6 | 01.01.2020 | 31.12.2020 | 43.81 | 20.13 | [0.00 – 63.33] |
|  |  | Silva-Valencia et al. 2024 | in person visits | 1.26 | 0.01 | [1.23 – 1.28] | Western Pacific | China | B | 6 | 01.04.2020 | 28.02.2021 | 70.34 | 7.34 | [52.68 – 77.98] |
|  |  | Silva-Valencia et al. 2024 | in person visits | 0.42 | 0.01 | [0.40 – 0.43] | Americas | USA | B | 6 | 01.04.2020 | 28.02.2021 | 65.24 | 2.35 | [59.52 – 69.29] |
|  |  | Ettman et al. 2024 | in-person appointments | 0.29 | 0.00 | [0.28 – 0.30] | Americas | USA | B | 6 | 15.03.2020 | 31.10.2022 | 56.94 | 9.28 | [39.29 – 69.29] |
|  |  | Lee et al. 2024 | mental healthcare visits | 1.02 | 0.00 | [1.01 – 1.02] | Americas | USA | B | 6 | 13.03.2020 | 31.12.2020 | 63.87 | 4.03 | [32.14 – 69.29] |
|  |  | Abe et al. 2025 | outpatient cases | 0.94 | 0.00 | [0.93 – 0.95] | Western Pacific | Japan | B | 6 | 01.04.2020 | 31.12.2020 | 43.17 | 3.98 | [35.12 – 50.00] |
|  |  |  |  |  |  |  |  |  |  |  |  |  |  |  |  |

**Table 2 (cont.) – Page 3**

|  |  |  |  |  |  |  |  |  |  |  |  |  |  |  |  |
| --- | --- | --- | --- | --- | --- | --- | --- | --- | --- | --- | --- | --- | --- | --- | --- |
| **Table 2 (cont.) – Page 4** |  | Fuster-Casanovas et al. 2024 | primary care utilization | 0.57 | 0.01 | [0.54 – 0.59] | Europe | Spain | B | 6 | 01.01.2020 | 31.12.2020 | 49.24 | 20.52 | [0.00 – 71.31] |
|  | Rand. Effects Model A, B |  |  | **0.79** |  | **[0.65 – 0.97]** |  |  |  |  |  |  |  |  |  |
|  |  | Andersson et al. 2022 | outpatient contacts to mental health services | 0.93 | 0.03 | [0.87 – 0.99] | Europe | Sweden | C | 5 | 01.01.2020 | 31.12.2020 | 44.38 | 18.25 | [0.00 – 61.79] |
|  |  | Minian et al. 2021 | outpatient contacts to mental health services | 0.76 | 0.01 | [0.74 - 0.78] | Americas | Canada | C | 5 | 17.03.2020 | 07.12.2020 | 62.49 | 2.25 | [42.26 – 65.48] |
|  |  | Lin et al. 2023 | outpatient psychiatry program | 1.02 | 0.01 | [0.99 – 1.05] | Americas | USA | C | 5 | 01.03.2020 | 28.02.2021 | 63.27 | 8.33 | [19.05 – 69.29] |
|  |  | Caselli et al. 2023 | consultations in psychiatric outpatient clinics | 0.88 | 0.07 | [0.73 – 1.02] | Europe | Italy | C | 5 | 01.03.2020 | 31.05.2021 | 73.60 | 6.64 | [59.23 – 85.42] |
|  |  | Savić et al. 2022 | outpatient consultations | 0.88 | 0.01 | [0.85 – 0.91] | Europe | Kroatia | C | 5 | 01.01.2020 | 31.12.2020 | 47.00 | 21.69 | [0.00 – 80.95] |
|  |  | Lee et al. 2023 | outpatient psychiatric services | 1.07 | 0.00 | [1.06 – 1.08] | Americas | USA | C | 5 | 01.01.2020 | 31.12.2020 | 53.49 | 21.67 | [0.00 – 69.29] |
|  |  | Salamah et al. 2024 | psychiatry outpatient clinic | 0.75 | 0.06 | [0.62 – 0.86] | Eastern Mediterranean | United Arab Emirates | C | 5 | 01.01.2020 | 31.12.2020 | 49.72 | 23.05 | [0.00 – 83.93] |
|  | Rand. Effects Model C |  |  | **0.90** |  | **[0.81 – 0.99]** |  |  |  |  |  |  |  |  |  |
| **telemedicine short term**  **cut-off: 8 months** |  | Ahmedani et al. 2024 | psychotherapy visits virtual | 15.6 | 0.01 | [2.73 – 2.77] | Americas | USA | A | 6 | 14.03.2020 | 31.12.2020 | 63.98 | 3.59 | [35.71 – 69.29] |
|  |  | Zhang et al. 2022 | tele-mental health | 5.42 | 0.00 | [5.40 – 5.42] | Americas | USA | A | 6 | 18.03.2020 | 05.05.2020 | 62.64 | 3.52 | [48.21 – 64.88] |
|  |  | Patel et al. 2021 | telemedicine (e.g. video- or telephone calls) | 3.44 | 0.01 | [3.40 – 3.48] | Europe | United Kingdom | A | 6 | 01.03.2020 | 30.06.2020 | 54.13 | 17.30 | [9.25 – 67.56] |
|  |  | Sanchez-Guarnido 2022 total | telemedicine (e.g. video- or telephone calls) | 13.6 | 0.49 | [5.18 – 35.87] | Europe | Spain | B | 6 | 16.03.2020 | 16.05.2020 | 61.75 | 4.50 | [50.3 – 67.86] |
|  | Rand. Effects Model A, B |  |  | **7.57** |  | **[3.63 – 15.77]** |  |  |  |  |  |  |  |  |  |
|  |  | Chow et al. 2021 | telemedicine (e.g. video- or telephone calls) | 3229 | 0.32 | [1716 – 6037] | Europe | Netherlands | C | 5 | 01.04.2020 | 30.06.2020 | 60.58 | 3.11 | [55.59 – 63.69] |
|  |  | Di Lorenzo et al. 2021a | telemedicine (e.g. video- or telephone calls) | 5.00 | 0.22 | [3.26 – 7.68] | Europe | Italy | C | 5 | 01.03.2020 | 31.08.2020 | 70.76 | 6.71 | [56.85 – 85.42] |
|  |  | Lee et al. 2020 | telemedicine (e.g. video- or telephone calls) | 2.29 | 0.08 | [1.95 – 2.70] | Western Pacific | China | C | 5 | 01.02.2020 | 30.04.2020 | 67.58 | 5.30 | [59.23 – 73.21] |
|  | Rand. Effects Model C |  |  | **33.0** |  | **[0.37 – 2969.6]** |  |  |  |  |  |  |  |  |  |
| telemedicine longterm |  |  |  |  |  |  |  |  |  |  |  |  |  |  |  |
|  |  | Jones et al. 2023 | telemedicine contacts | 23.4 | 0.04 | [23.32 – 23.48] | Americas | USA | A | 6 | 01.09.2019 | 28.02.2021 | 55.55 | 20.75 | [0.00 – 69.29] |
|  |  | Silva-Valencia et al. 2024 | virtual visits | 3743 | 0.13 | [3743 – 3744] | Americas | Canada | A | 6 | 01.04.2020 | 28.02.2021 | 64.47 | 3.64 | [59.52 – 71.55] |
|  |  | Silva-Valencia et al. 2024 | virtual visits | 12.4 | 0.00 | [12.45 – 12.47] | Europe | Norway | A | 6 | 01.04.2020 | 28.02.2021 | 47.66 | 11.08 | [33.63 – 69.23] |
|  |  | Silva-Valencia et al. 2024 | virtual visits | 8.88 | 0.00 | [8.87 – 8.88] | Americas | Peru | A | 6 | 01.04.2020 | 28.02.2021 | 73.42 | 6.24 | [55.36 – 82.74] |
|  |  | Silva-Valencia et al. 2024 | virtual visits | 7.98 | 0.02 | [7.93 – 8.02] | Europe | Sweden | A | 6 | 01.04.2020 | 28.02.2021 | 55.55 | 4.32 | [51.19 – 64.42] |
|  |  | Rice et al. 2025 | phone and video mental health | 2.73 | 0.00 | [2.72 – 2.73] | Americas | USA | A | 6 | 11.03.2020 | 10.12.2020 | 63.26 | 4.88 | [26.79 – 67.86] |
|  |  | Sweet et al. 2022 | telemedicine (e.g. video- or telephone calls) | 1.17 | 0.00 | [1.17 – 1.17] | Americas | USA | B | 5 | 01.03.2020 | 01.02.2021 | 62.96 | 8.57 | [19.05 – 69.29] |
|  |  | Ettman et al. 2024 | appointments via video or telephone | 1816 | 0.11 | [1816 – 1816] | Americas | USA | B | 6 | 15.03.2020 | 31.10.2022 | 56.94 | 9.28 | [39.29 – 69.29] |
|  |  | Bhagavathula et al. 2024 | telehealth services (via video or telephone) | 46.8 | 0.00 | [46.79 – 46.81] | Americas | USA | B | 6 | 01.01.2020 | 31.12.2020 | 53.49 | 21.67 | [0.00 – 69.29] |
|  |  | Rachamin et al. 2023 | all psychotherapy teleconsultations | 0.62 | 0.11 | [0.39 – 0.83] | Europe | Switzerland | B | 6 | 01.01.2020 | 31.12.2020 | 43.81 | 20.13 | [0.00 – 63.33] |
|  |  | Fuster-Casanovas et al. 2024 | primary care utilization, telemedicine | 6.33 | 0.02 | [6.28 – 6.38] | Europe | Spain | B | 6 | 01.01.2020 | 31.12.2020 | 49.24 | 20.52 | [0.00 – 71.31] |
|  | Rand. Effects Model A, B |  |  | **18.3** |  | **[3.63 – 93.08]** |  |  |  |  |  |  |  |  |  |
| **medication short term**  **cut-off: 8 months** |  | Engels et al. 2022 | medication prescriptions | 1.02 | 0.00 | [1.02 – 1.02] | Europe | Germany | A | 6 | 01.03.2020 | 31.05.2020 | 56.52 | 13.06 | [27.98 – 70.83] |
|  |  | Ying et al. 2023 | medication dispensing | 1.09 | 0.00 | [1.09 – 1.09] | Americas | Canada | A | 6 | 01.02.2020 | 31.03.2020 | 22.79 | 22.57 | [7.74 – 62.20] |
|  |  | Moreno-Martos et al. 2024 | dispensed medications | 0.92 | 0.01 | [0.90 – 0.94] | Europe | Norway | A | 6 | 01.03.2020 | 31.03.2020 | 32.52 | 17.86 | [9.52 – 53.57] |
|  |  | Moreno-Martos et al. 2024 | dispensed medications | 0.96 | 0.01 | [0.93 – 0.98] | Europe | Sweden | A | 6 | 01.03.2020 | 31.03.2020 | 33.98 | 11.20 | [13.10 – 48.81] |
|  |  | Carr et al. 2021 | medication prescriptions | 0.68 | 0.01 | [0.67 – 0.69] | Europe | United Kingdom | A | 6 | 01.04.2020 | 01.05.2020 | 60.82 | 0.30 | [60.71 – 61.61] |
|  |  | McKee et al. 2021 | medication prescriptions | 1.05 | 0.02 | [1.01 – 1.09] | Americas | Canada | A | 6 | 01.03.2020 | 31.05.2020 | 53.85 | 18.31 | [7.74 – 63.39] |
|  |  | Williams et al. 2020 | medication prescriptions | 0.61 | 0.06 | [0.54 – 0.69] | Europe | United Kingdom | B | 6 | 01.03.2020 | 31.05.2020 | 50.26 | 18.35 | [9.25 – 62.2] |
|  | Rand. Effects Model A, B |  |  | **0.90** |  | **[0.77 – 1.05]** |  |  |  |  |  |  |  |  |  |
|  |  | Fstkchian et al. 2023 | medications prescriptions | 1.14 | 0.51 | [0.13 – 2.16] | Americas | USA | C | 5 | 01.03.2020 | 30.04.2020 | 53.00 | 16.24 | [19.05 – 64.88] |
|  |  | Fu et al. 2024 | average monthly number of prescriptions | 1.46 | 1.33 | [-1-14 – 4.07] | Europe | United Kingdom | C | 5 | 23.03.2020 | 03.07.2020 | 62.20 | 3.12 | [56.25 – 67.56] |
|  |  | Zhang et al. 2022 | medications prescriptions | 0.98 | 0.00 | [0.98 – 0.98] | Americas | USA | C | 5 | 18.03.2020 | 05.05.2020 | 62.64 | 3.52 | [48.21 – 64.88] |
|  | Rand. Effects Model C |  |  | **0.98** |  | **[0.98 – 0.98]** |  |  |  |  |  |  |  |  |  |
| medication  long term |  | Engels et al. 2022 | medication prescriptions | 0.94 | 0.00 | [0.94 – 0.94] | Europe | Germany | A | 6 | 01.01.2020 | 31.12.2020 | 50.51 | 19.92 | [0.00 – 73.69] |
|  |  | Ludwig et al. 2022 | medication prescriptions | 1.03 | 0.00 | [1.03 – 1.03] | Europe | Germany | A | 6 | 01.01.2020 | 31.12.2020 | 50.51 | 19.92 | [0.00 – 73.69] |
|  |  | Di Valerio et al. 2024 | medication dispensing | 0.98 | 0.00 | [0.97 – 0.98] | Europe | Italy | A | 6 | 01.01.2020 | 31.12.2020 | 61.73 | 22.44 | [0.00 – 85.42] |
|  |  | Jones et al. 2023 | receipt of medications | 0.78 | 0.01 | [0.75 – 0.80] | Americas | USA | A | 6 | 01.09.2019 | 28.02.2021 | 55.55 | 20.75 | [0.00 – 69.29] |
|  |  | Lieber et al. 2024 | medication prescriptions | 0.99 | 0.00 | [0.97 – 1.00] | Europe | Sweden | A | 6 | 01.01.2020 | 31.12.2020 | 44.38 | 18.28 | [0.00 – 61.70] |
|  |  | Luo et al. 2024 | psychotropic drug prescribing | 1.03 | 0.16 | [0.70 – 1.35] | Americas | USA | A | 6 | 01.04.2020 | 31.12.2020 | 64.59 | 2.00 | [59.52 – 69.29] |
|  |  | Chu et al. 2024 | mental health and addiction prescription claim | 1.01 | 0.00 | [1.00 – 1.02] | Americas | Canada | A | 6 | 01.06.2021 | 01.06.2022 | 60.41 | 12.69 | [32.77 – 73.21] |
|  |  | Visser et al. 2025 | numbers of dispensed defined daily doses | 1.06 | 0.00 | [1.05 – 1.05] | Europe | Netherlands | A | 6 | 09.03.2020 | 13.03.2022 | 59.20 | 8.63 | [19.05 – 76.19] |
|  |  | Moreno-Martos et al. 2024 | dispensed medications | 1.01 | 0.00 | [1.00 – 1.01] | Europe | Norway | A | 6 | 01.03.2020 | 31.12.2020 | 42.62 | 9.80 | [9.52 – 57.14] |
|  |  | Moreno-Martos et al. 2024 | dispensed medications | 1.00 | 0.00 | [1.00 – 1.01] | Europe | Sweden | A | 6 | 01.03.2020 | 31.12.2020 | 51.81 | 7.33 | [13.10 – 61.79] |
|  |  | Rice et al. 2025 | medication prescriptions | 1.24 | 0.00 | [1.23 – 1.24] | Americas | USA | A | 6 | 11.03.2020 | 10.12.2020 | 63.26 | 4.88 | [26.79 – 67.86] |
|  |  | Luo et al. 2024 | psychotropic drug prescribing | 0.96 | 0.20 | [0.57 – 1.33] | Europe | Germany | B | 6 | 01.04.2020 | 31.12.2020 | 60.62 | 4.53 | [54.46 – 73.69] |
|  |  | Luo et al. 2024 | psychotropic drug prescribing | 1.01 | 0.13 | [0.75 – 1.27] | Europe | France | B | 6 | 01.04.2020 | 31.12.2020 | 60.41 | 9.02 | [46.43 – 72.32] |
|  |  | Luo et al. 2024 | psychotropic drug prescribing | 1.00 | 0.13 | [0.73 – 1.27] | Europe | Italy | B | 6 | 01.04.2020 | 31.12.2020 | 70.77 | 6.37 | [62.20 – 85.42] |
|  |  | Luo et al. 2024 | psychotropic drug prescribing | 1.03 | 0.15 | [0.73 – 1.33] | Europe | United Kingdom | B | 6 | 01.04.2020 | 31.12.2020 | 63.47 | 3.89 | [56.25 – 76.31] |
|  |  | Rachamin et al. 2023 | psychotropic mediation claims | 0.21 | 0.04 | [0.12 – 0.31] | Europe | Switzerland | B | 6 | 01.01.2020 | 31.12.2020 | 43.81 | 20.13 | [0.00 – 63.33] |
|  | Rand. Effects Model A, B |  |  | **0.91** |  | **[0.74 – 1.11]** |  |  |  |  |  |  |  |  |  |
|  |  | Luo et al. 2024 | psychotropic drug prescribing | 1.01 | 0.11 | [0.78 – 1.24] | Western Pacific | South Korea | C | 5 | 01.04.2020 | 31.12.2020 | 56.50 | 5.87 | [47.32 – 73.81] |
|  |  | Rugova et al. 2024 | daily doses marketed per 1000 inhabitants | 1.66 | 0.30 | [1.05 – 2.26] | Europe | Kosovo | C | 5 | 01.01.2020 | 31.12.2020 | 49.77 | 25.43 | [0.00 – 72.02] |
|  |  | Zaki et al. 2022 | admissions to acute psychiatric units | 1.38 | 0.01 | [1.36 – 1.40] | Western Pacific | Australia | C | 5 | 01.01.2020 | 31.12.2020 | 52.36 | 21.19 | [0.00 – 72.62] |
|  | Rand. Effects Model C |  |  | **1.26** |  | **[0.98 – 1.63]** |  |  |  |  |  |  |  |  |  |

**Table 2 (cont.) – Page 5**
